# Bioactive Bianthraquinones and Meroterpenoids from a Marine-Derived *Stemphylium* sp. Fungus

**DOI:** 10.3390/md18090436

**Published:** 2020-08-21

**Authors:** Ji-Yeon Hwang, Sung Chul Park, Woong Sub Byun, Dong-Chan Oh, Sang Kook Lee, Ki-Bong Oh, Jongheon Shin

**Affiliations:** 1 Natural Products Research Institute, College of Pharmacy, Seoul National University, San 56-1, Sillim, Gwanak, Seoul 151-742, Korea; yahyah7@snu.ac.kr (J.-Y.H.); sungchulpark@snu.ac.kr (S.C.P.); sky_magic@naver.com (W.S.B.); dongchanoh@snu.ac.kr (D.-C.O.); sklee61@snu.ac.kr (S.K.L.); 2 Department of Agricultural Biotechnology, College of Agriculture and Life Science, Seoul National University, San 56-1, Sillim, Gwanak, Seoul 151-921, Korea

**Keywords:** bianthraquinones, meroterpenoids, marine-derived fungus, anti-inflammatory activity, *Stemphylium* sp.

## Abstract

Three new bianthraquinones, alterporriol Z1–Z3 (**1**–**3**), along with three known compounds of the same structural class, were isolated from the culture broth of a marine-derived *Stemphylium* sp. fungus. Based upon the results of spectroscopic analyses and ECD measurements, the structures of new compounds were determined to be the 6-6′- (**1** and **2**) and 1-5′- (**3**) C–C connected *pseudo*-dimeric anthraquinones, respectively. Three new meroterpenoids, tricycloalterfurenes E–G (**7**–**9**), isolated together with the bianthraquinones from the same fungal culture broth, were structurally elucidated by combined spectroscopic methods. The relative and absolute configurations of these meroterpenoids were determined by modified Mosher’s, phenylglycine methyl ester (PGME), and computational methods. The bianthraquinones significantly inhibited nitric oxide (NO) production and suppressed inducible nitric oxide synthase (iNOS) and cyclooxygenase-2 (COX-2) expression in LPS-stimulated RAW 264.7 cells.

## 1. Introduction

Polyketides are a representative class of fungal metabolites [1,2]. These compounds possess a vast range of structural diversity resulting in several subgroups such as anthraquinones, naphthoquinones, benzophenones, xanthones, flavonoids, macrolides, and tropolones [3,4]. Among these, anthraquinones are biosynthetically derived from an octaketide chain formed by the incorporation of one acetyl-CoA and seven malonyl-CoA units [5,6,7]. Although widely distributed in fungi, anthraquinone derivatives occur far frequently in the genera of *Alternaria*, *Aspergillus*, *Fusarium*, *Stemphylium*, and *Trichoderma* [8]. Anthraquinones typically contain various substituents (methyl, hydroxyl, methoxyl, or more complex substituents), often attributed to characteristic colors by their properties and positions at the aromatic core moiety [9,10].

A frequently encountered structural variation of fungal anthraquinones is the dimerization through a C–C bond formation between two similar units [3]. The dimerization patterns for these bianthraquinones are also diversified through both homo- and hetero- bond formation, providing additional structural variation to the anthraquinones. To date, a vast number of fungal examples were reported including alterporriols, icterinoidin, rubellin, and skyrin [3,11]. In addition to their monomeric precursors, bianthraquinones often co-occur with biosynthetically related compounds of the polyketide pathway [4,12]. Wide structural diversity, in conjunction with significant bioactivities such as antibacterial, anti-inflammatory, antituberculosis, and cytotoxic activities, designates bianthraquinones as an important group of fungal polyketides [3,8,11,13].

Meanwhile, tricycloalternarenes (TCAs) are a structural class of fungal meroterpenoids [14]. Structurally, these are closely related to the ACTG toxins with differences at the isoprenoid side chain and the substitution pattern at the C-ring of TCAs [15]. Biosynthetically, these meroterpenoids are proposed to be generated from a hybrid shikimate–isoprenoid route [16]. Exclusively found in *Alternaria* and *Guignardia*, tricycloalternarene-type meroterpenoids are regarded as one of the key chemical characteristics of these fungal genera [17,18]. These compounds were also reported to exhibit diverse bioactivities, including antimicrobial, cytotoxic, and NF-κB-inhibitory [19,20].

**Figure 1:**
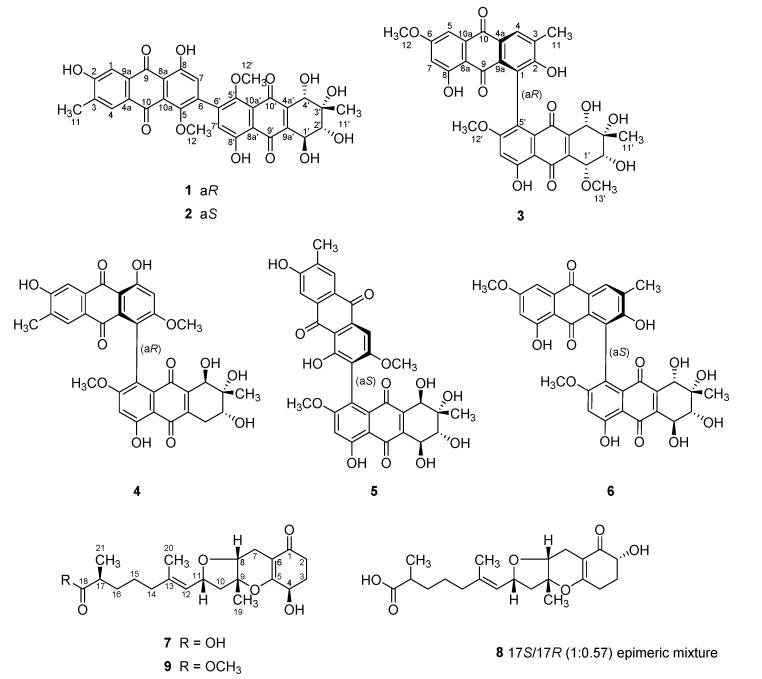
Structures of **1**–**9**.

During the course of a search for bioactive compounds from marine-derived fungi, we isolated a strain (strain number FJJ006) of *Stemphylium* sp. from an unidentified sponge specimen collected off the coast of the island of Jeju-do, Korea. LC-ESIMS and LC-UV analyses of the culture broth of this strain showed the presence of several compounds with various profiles, prompting extensive chemical investigation. The large-scale cultivation, sequentially followed by extraction, solvent-partitioning, and chromatographic separations, afforded six new and three known compounds (Figure 1). Here, we report the structures of bianthraquinone alterporriols Z1–Z3 (**1**–**3**) and meroterpenoid tricycloalterfurenes E–G (**7**–**9**). This is first time isolating not only the tricycloalternarene-type meroterpenoids but also their co-occurrence with polyketide-derived anthracenes from the fungus *Stemphylium.* Compounds **1** and **2** exhibited moderate anti-inflammatory activity in LPS-stimulated RAW 264.7 cells.

## 2. Results and Discussion

Compound **1** was an orange amorphous powder which was analyzed to have the molecular formula of C_32_H_26_O_13_, with 20 unsaturation degrees, by HRFABMS analysis ([M + H]^+^ *m*/*z* 619.1449, calcd C_32_H_27_O_13_, 619.1446). The ^13^C NMR data of this compound showed signals of four carbonyl (δc 190.5, 188.7, 185.7, 183.5) and twenty deshielded methine and quaternary carbons (δc 166.9–104.6). Aided by the HSQC data, the chemical shifts of corresponding methine protons at δ_H_ 7.65, 7.51, 6.81, and 6.78 in the ^1^H NMR data revealed the presence of aromatic moieties (Table 1). Since these NMR features were accounted for 14 unsaturation degrees, **1** must be a hexacyclic compound.

A combination of ^13^C and ^1^H NMR and HSQC data diagnosed the remaining NMR signals as an oxy-quaternary carbon (δc 74.6) and three oxymethine (δ_C_/δ_H_ 75.2/3.79, 70.6/4.73, and 70.1/4.26), two oxymethyl (δ_C_/δ_H_ 57.0/3.70 and 56.9/3.69), and two methyl (δ_C_/δ_H_ 22.3/1.33 and 16.6/2.23) groups. Aided by the literature study, the overall spectroscopic features suggested **1** to be a bianthraquinone consisting of each one unit of anthraquinone and tetrahydroanthraquinone.

**Figure 2:**
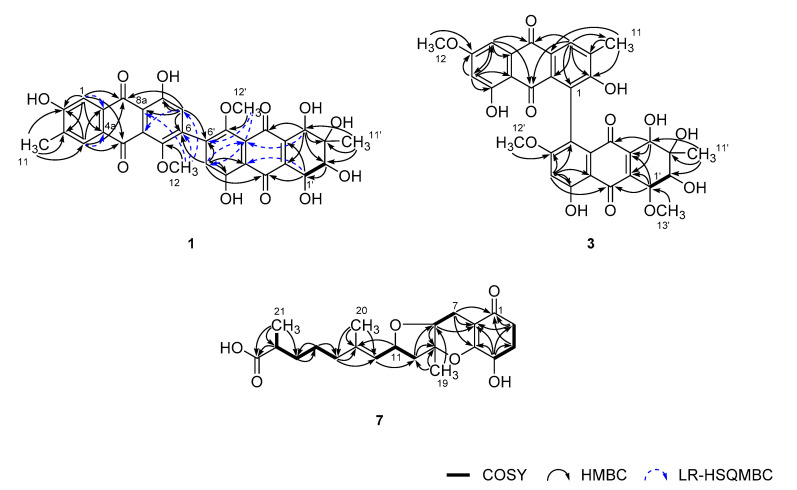
Key correlations of COSY (bold), HMBC (arrow), and LR-HSQMBC (*J*_CH_ = 2 Hz, dashed arrow) experiments for compounds **1**, **3**, and **4**.

Having the information above, the structure determination of **1** was mostly accomplished by extensive H–C long-range analyses for this proton-deficient compound (Figure 2). Firstly, long-range correlations of two aromatic protons at δ_H_ 7.51 (H-1) and 7.65 (H-4) and a benzylic methyl proton at δ_H_ 2.23 (H_3_-11) with neighboring carbons not only constructed a 2-hydroxy-3-methylbenzene moiety (C-1-C-4, C-4a, and C-9a) but also placed two carbonyls at δ_C_ 188.7 (C-9) and 183.5 (C-10) *ortho*-substituted to the benzene. Similarly, combined HMBC and LR-HSQMBC correlations of the protons at δ_H_ 6.78 (H-7) and 3.69 (H_3_-12) with neighboring carbons revealed the presence of an 8-hydroxy-5-methoxybenzene moiety (C-5-C-8, C-8a, and C-10a). The linkage between C-8a and C-9 was also secured by crucial H-7/C-9 correlation. Although it was not evidenced by HMBC data, the carbon chemical shifts of C-10 (δ_C_ 183.5) and C-10a (δ_C_ 133.4), in conjunction with the MS-derived unsaturation degree, directly linked these carbons, thus, constructing an anthraquinone moiety for **1**.

Meanwhile, ^1^H–^1^H COSY data showed direct spin coupling (*J* = 7.5 Hz) between oxymethine protons at δ_H_ 4.73 (H-1′) and 3.79 (H-2′) (Figure 2). Subsequently, HMBC correlations of these protons and an isolated oxymethine and a methyl proton at δ_H_ 4.26 (H-4′) and 1.33 (H-11′), respectively, with the olefinic and hydroxyl-bearing carbons, defined a 3-methyl-2,3,4,5-tetrahydroxy cyclohexene-type moiety (C-1′–C-4′, C-4a’, and C-9a’). The attachment of two carbonyl carbons at δ_C_ 190.5 (C-9′) and 185.7 (C-10′) at the cyclohexene unit was also accomplished by their HMBC correlations with H-4′ and H-1′, respectively.

The HMBC correlations of an aromatic and a methoxy proton at δ_H_ 6.81 (H-7′) and 3.70 (H_3_-12′), respectively, with aromatic carbons, revealed an 8′-hydroxy-5′-methoxybenzene (C-5′–C-8′, C-8a’, and C-10a’), analogous to the C-5–C-10a unit. Subsequently, further connection to the diketo-bearing cyclohexene unit constructing a tetrahydroanthraquinone moiety was also accomplished by the HMBC and LR-HSQMBC correlations between these. Since the anthraquinone and tetrahydroanthraquinone moieties had open ends at C-6 and C-6′, respectively, C–C linkage between these carbons was anticipated. The crucial evidence was provided by the HMBC data, in which long-range correlations were found at H-7/C-6′ and H-7′/C-6. Thus, the planar structure of **1** was determined as a bianthraquinone analogous to alterporriols [3]. Although significant numbers of bianthraquinones were reported from diverse fungi, those having a 6-6′ C–C linkage are rather rare. To the best of our knowledge, alterporriol K–M are the only previous examples having the same type of C–C linkage [21].

**Figure 3:**
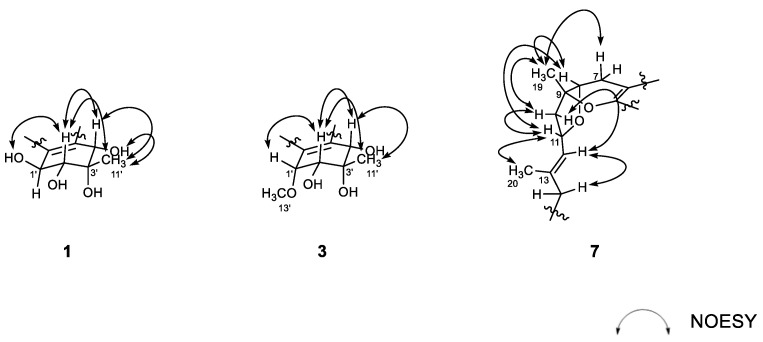
Key correlations of NOESY (arrow) experiments for compounds **1**, **3**, and **7**.

The structure of **1**, designated as alterporriol Z1, possessed four stereogenic centers at C-1′–C-4′ at the aliphatic ring and a chiral axis at C-6/C-6′, requiring configurational determination. Firstly, relative configurations at the aliphatic ring were assigned by proton–proton coupling constants and NOESY analyses (Figure 3). The large coupling (*J* = 7.5 Hz) between H-1′ and H-2′ oriented both protons axial to the cyclohexene ring that was supported by the NOESY cross peak at H-2′/1′-OH. Then, the neighboring 11′-CH_3_ group was equatorially oriented by the cross peak H-2′/H_3_-11′. Although both H-4′ and 4′-OH protons showed spatial proximity with H_3_-11′, the NOESY cross peak at H-2′/H-4′ was crucial to the axial orientation of H-4′ (Supplementary Materials, Figure S9 and Table S2). Thus, the overall relative configurations were assigned as 1′S*, 2′R*, 3′S*, and 4′S*. The absolute configurations at these centers were approached by computational methods and are described later.

**Figure 4:**
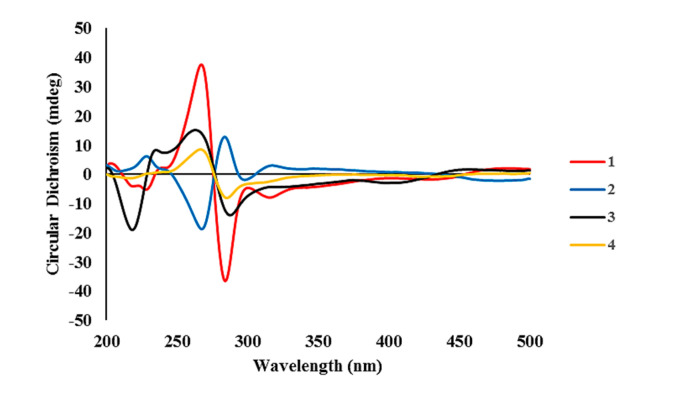
Experimental ECD spectra of compounds **1**–**4**.

**Figure 5:**
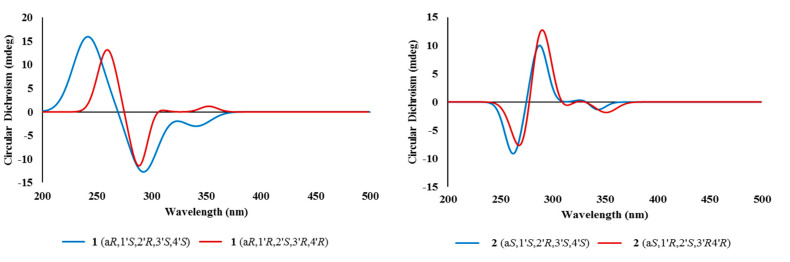
Calculated ECD spectra of compounds **1** and **2**.

The configuration at the C-6/C-6′ chiral axis was assigned by ECD measurements. As shown in Figure 4, the measured ECD profile of **1** showed significant Cotton effects at 269 (Δε 35.79) and 285 (Δε −36.06) nm, assigning an a*R* configuration at the C-6/C-6′ axis. Although this interpretation was supported by the same configuration at structurally related alterporriols A and L [21,22], a question would arise from the possible contribution of ECD by the C-1′–C-4′ stereogenic centers. That is, the absolute configurations at these centers would remarkably influence overall ECD profiles. Conversely, depending on the results, it would be also possible to deduce the absolute configurations of stereogenic centers at the cyclohexene moiety. To clarify this, ECD data were calculated for the a*R* atropisomeric contribution with two possible absolute configurations at the stereogenic centers. The results were that these profiles were very similar to each other at both wavelengths and intensity of Cotton effects, regardless of the configurations at the cyclohexene moiety (Figure 5). Thus, the a*R* configuration was unambiguously assigned for the chiral axis.

Having fixed axial chirality, the absolute configurations at C-1′–C-4′ were subsequently approached by DP4 calculations. However, two models derived from the opposite configurations expected very similar ^13^C and ^1^H NMR data from each other (Supplementary Materials, Figure S52 and Table S1), which was consistent with the results of ECD analysis. Thus, the absolute configurations of the cyclohexene moiety remained unassigned [23]. Overall, the structure of alterporriol Z1 (**1**) was determined to be a bianthraquinone of the alterporriol class.

The molecular formula of alterporriol Z2 (**2**) was established to be C_32_H_26_O_13_, identical to **1**, by HRFABMS analysis ([M + H]^+^ *m*/*z* 619.1454, calcd for C_32_H_27_O_13_, 619.1446). A comparison of the ^13^C and ^1^H NMR data of this compound with those of **1** revealed a very close structural similarity between them (Table 1). Subsequently, the extensive 1D and 2D NMR analyses of **2** deduced the same planar structure and relative configurations of the aliphatic ring as those of 1. Accordingly, the structural difference was traced to the chiral axis at C-6–C-6′, in which the measured ECD spectrum of **2** showed a quasi-mirror profile from **1** (Figure 4). The noticeably reduced intensities of Cotton effects would be attributed from the stereogenic center-bearing cyclohexene moiety. However, the calculated ECD data of **2** were virtually identical to each other, regardless of absolute configurations at the cyclohexene stereogenic centers, eradicating the possibility of the reversal of measured ECD by these factors (Figure 5). Thus, the structure of alterporriol Z2 (**2**) was defined as an atropisomer of alterporriol Z1 (1).

A minor constituent of alterporriol Z3 (**3**) was isolated as a dark red amorphous solid that was analyzed for the molecular formula of C_33_H_28_O_13_ by HRFABMS analysis ([M + Na]^+^ *m*/*z* 655.1430, calcd for C_33_H_28_O_13_Na, 655.1422). The ^13^C and ^1^H NMR data of this compound were similar to those of **1** and **2**, indicating the same bianthraquinone nature (Table 1). An extensive examination of these data, in conjunction with a literature and database search, revealed that **3** had close structural similarity with a congener alterporriol N (**6**) [23]. The most conspicuous difference in NMR data was the presence of an additional methoxy group at δ_C_/δ_H_ 62.8/3.77 in **3**.

Given this information, the planar structure of **3** was determined by HMBC analyses. Despite the limited amount of obtained materials, almost all carbons and protons except for unprotonated C-10a’ were adequately assigned by HSQC and HMBC correlations (Figure 2). In this way, **3** was defined as consisting of an anthraquinone and a tetrahydroanthraquinone moiety, as with other compounds. The newly appeared methoxy group (C-13′) was also attached at C-1′ by COSY correlation at H-1′/H-2′ and HMBC correlation at H_3_-13′/C-1′. Due to the lack of neighboring protons, the C–C linkage was not directly evidenced by HMBC data. However, the diagnostic chemical shifts of the unprotonated C-1 and C-5′ carbons at δ_C_ 129.0 and 130.7, respectively, were indicative of the linkage between the anthraquinone and tetrahydroanthraquinone moiety at these carbons.

The configurations at cyclohexene and C-1/C-6′ chiral axis of **3** were assigned using the same methods as in **1** and **2**. Firstly, the small coupling constants (*J* = 4.7 Hz) between the vicinal H-1′ and H-2′ indicated the orientations of these to be either axial–equatorial or equatorial–equatorial to the cyclohexene ring. Then, the NOESY data showed mutual cross peaks among H-2′, H-4′, and H_3_-11′, placing these at the same phase of the cyclohexene ring (Figure 3). Therefore, the former two oxymethine protons were axially oriented, while the C-11′ methyl group was equatorially oriented to the ring system. Aided with the additional cross peak with H-2′, the H-1′ oxymethine proton was also equatorially oriented. Thus, the overall relative configurations were assigned as 1′R*, 2′R*, 3′S*, and 4′S*. The absolute configuration of the C-1/C-6′ chiral axis of **3** was also assigned by ECD measurement. As shown in Figure 4, the ECD data of this compound were similar to 1, assigning the same a*R* configuration. Thus, the structure of alteroporriol Z3 (**3**) was defined as a new bianthraquinone of the alterporriol class.

In addition to **1**–**3**, the crude extract of *Stemphylium* sp. contained three known bianthraquinones (**4**–**6**). Based upon the results of combined spectroscopic analyses, these were identified as alterporriols F (**4**) [24], G (**5**) [25], and N (**6**) [23], respectively. The spectroscopic data of these compounds were in good accordance with those in the literature. Among these, the configuration of C-5/C-5′ chiral axis of **4**, previously unassigned, was determined to be a*R* by the ECD measurement in this work (Figure 4).

In addition to bianthraquinones, the culture broth of *Stemphylium* sp. contained three new meroterpenoids. The molecular formula of compound **7**, a brown amorphous solid, was deduced to be C_21_H_30_O_6_ with seven unsaturation degrees, by HRFABMS analysis ([M + H]^+^ *m*/*z* 379.2118, calcd for C_21_H_31_O_6_, 379.2115). The ^13^C NMR data of this compound showed signals of three significantly deshielded carbons (δ_C_ 200.4, 180.9, and 170.4) and three olefinic carbons (δ_C_ 139.7, 127.5, and 107.5). The odd numbers of the latter carbons were indicative of a highly differentiated double bond possibly consisting of a deshielded carbon (δ_C_ 170.4) and a shielded carbon (δ_C_ 107.5). Between two carbonyl carbons, the conspicuous one (δ_C_ 200.4) was readily assigned as a ketone, while the other one (δ_C_ 180.9) was thought to be either a carboxylic acid or an ester group by a strong absorption band at 1755 cm^−1^ in the IR data. Aided by the ^1^H NMR and HSQC data, the remaining 15 carbons in the ^13^C NMR data were diagnosed to be one oxy-quaternary (δ_C_ 85.8), three oxymethine (δ_C_ 78.1, 74.5, and 66.8), one methine (δ_C_ 40.8), seven methylene (δ_C_ 46.8, 40.2, 34.5, 33.6, 30.5, 26.3, and 19.8), and three methyl (δ_C_ 20.1, 17.7, and 16.4) carbons (Table 2). Overall, preliminary examination of the spectroscopic data suggested **7** to be a tricyclic compound possessing two carbonyl groups and two double bonds.

The planar structure of **7** was determined by combined ^1^H–^1^H COSY and HMBC analyses (Figure 2). That is, COSY data revealed a linear assembly of an oxymethine and two methylene groups (δ_C_/δ_H_ 33.6/2.62 and 2.32, 30.5/2.18 and 1.97, and 66.8/4.30, C-2–C-4). Then, the HMBC correlations of these protons with neighboring carbons not only placed a ketone (δ_C_ 200.4, C-1) and a tetra-substituted double bond (δ_C_ 170.4 and 107.5, C-6 and C-5) at the adjacent locations, but also constructed a 4-oxygenated cyclohexanone moiety (C-1–C-6, ring A): H-4/C-2, C-3, C-5, and C-6, H_2_-3/C-1, and H_2_-2/C-1 and C-6. The COSY data also found a direct linkage between methylene and oxymethine (δ_C_/δ_H_ 19.8/2.59 and 2.22 and 78.1/3.95, C-7 and C-8). Subsequently, their attachment at C-6 of ring A was secured by the HMBC of H_2_-7 with ring carbons: H_2_-7/C-1, C-5, and C-6. Similarly, a quaternary carbon (δ_C_ 85.8, C-9) and a methyl group (δ_C_/δ_H_ 20.1/1.35, C-19) were linearly connected at C-8 by a number of key HMBC correlations: H-8/C-9 and H_3_-19/C-8 and C-9.

The COSY data revealed the presence of another linear spin system consisting of each one of methylene, oxymethine, and olefinic methine (δ_C_/δ_H_ 46.8/2.42 and 2.07, 74.5/4.80, and 127.5/5.17, C-10–C-12; Figure 2). The accomplishment of a full olefin (with δ_C_ 139.7, C-13) as well as the attachment of a vinyl methyl group (δ_C_/δ_H_ 16.4/1.65, C-20) was made by HMBC correlations: H-11/C-13 and H_3_-20/C-12 and C-13. The linkage of this moiety at the C-9 quaternary carbon was also secured by HMBC data: H_3_-19/C-10 and H_2_-10/C-8 and C-9.

Based on the COSY data, the remaining proton signals were found to form a linear assembly of three methylenes, a methine, and a methyl group (δ_C_/δ_H_ 40.2/1.99 (2H); 26.3/1.42 (2H); 34.5/1.59 and 1.38, 40.8/2.37; and 17.7/1.11, C-14-C-17, and C-21). Subsequently, the attachments of this moiety at C-13 and a carbonyl group (δ_C_ 180.9, C-18) were secured by a series of HMBC correlations: H_3_-20/C-14; H_2_-14/C-12; and C-13, H-17/C-18, and H_3_-21/C-18. Thus, the framework of **7** was constructed as a C_21_ meroterpenoid of the tricycloalterfurene class.

The 2D NMR-based structure elucidation of **7** assigned, in addition to the C-1 ketone, six oxygenated positions at C-4, C-5, C-8, C-9, C-11, and C-18, accounting for two rings inherent from the mass data. A literature study suggested cyclic ether linkages at C-5/C-9 and C-8/C-11 constructing a dihydropyran (ring B) and a tetrahydrofuran (ring C), respectively, while the remaining carbons were functionalized as the 4-hydroxy and 21-carboxylic acid group. The comparison of ^13^C and ^1^H NMR data of **7** were in good accordance with tricycloalterfurene A [15], supporting this interpretation. Crucial evidence was provided in the process for relative and absolute configurations and described later.

The configurations at the stereogenic centers of **7** were initially approached by NOESY data (Figure 3). The 13*E* configuration was assigned by cross peaks at H-11/H_3_-20 and H-12/H_2_-14 as well as the diagnostic carbon chemical shifts of C-20 (δ_C_ 16.4) and C-14 (δ_C_ 40.2). The NOESY data also placed H-7β (δ_H_ 2.22), H-8, H_3_-19, H-10β (δ_H_ 2.42), and H-11 at one phase of the B/C ring plane by a series of cross peaks: H-7β/H_3_-19, H-8/H_3_-19, H-8/H-11, H_3_-19/H-10β, and H-10β/H-11. To have these cross peaks, the B/C ring juncture must have a *cis* orientation. Overall, relative configurations at the stereogenic centers were assigned as 8R*, 9R*, and 11R*.

**Figure 6:**
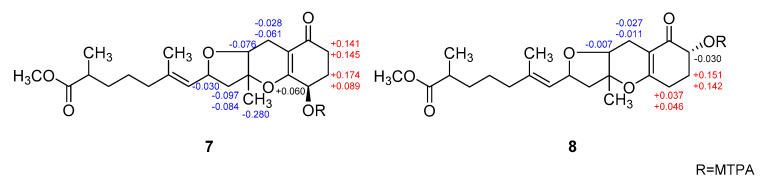
Δδ values [Δδ = δ*_S_* − δ*_R_*] obtained for (*S*)- and (*R*)-MTPA esters of **7** and **8**.

**Figure 7:**
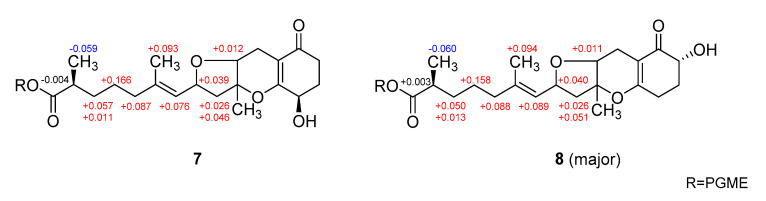
Δδ values [Δδ = δ*_S_* − δ*_R_*] obtained for (*S*)- and (*R*)-PGME amide derivatives of **7** and **8**.

The configurations at the remotely placed C-4 and C-17 stereogenic centers were independently assigned by modified Mosher’s and phenylglycine methyl ester (PGME) methods, respectively. That is, the treatments of **7** with (*R*)- and (*S*)-*α*-methoxy-*α*-(trifluoromethyl)-phenylacetyl chloride (MTPA-Cl) produced corresponding (*S*)- and (*R*)-MTPA esters, **7**-4*S* and **7**-4*R*, respectively. The resulting Δδ (δ*_S_* − δ*_R_*) values between these esters assigned the 4*R* absolute configuration (Figure 6). Similarly, **7** was also converted to (*S*)- and (*R*)-PGME amides, **7**-18*S* and **7**-18*R* by treatments with (*S*)- and (*R*)-PGME, respectively. Subsequently, the Δδ (δ*_S_* − δ*_R_*) values between the amides clearly assigned the 17*S* configuration (Figure 7). As described earlier, the productions of 4-esters and 17-amides by these reactions unambiguously confirmed the presence of 4-hydroxy and 18-carboxylic acid groups.

Finally, given the absolute configurations at the remote stereogenic centers, those at rings B and C were determined by computational methods. As shown in Figure 7, the measured ECD profile of **7** matched well with the calculated one, with 8*R*, 9*R*, and 11*R* configurations in both wavelength and intensity of the absorption maximum at 258 nm. Overall, the absolute configurations of **7** were determined to be 4*R*, 8*R*, 9*R*, 11*R*, and 17*S* by combined NOESY, chemical derivatization, and computational methods. Thus, the structure of **7**, designated as tricycloalterfurene E, was determined to be a tricyclic meroterpenoid.

The molecular formula of compound **8** was deduced to be C_21_H_30_O_6_, identical to **7**, by HRFABMS analysis ([M + H]^+^ *m*/*z* 379.2120, calcd for C_21_H_31_O_6_, 379.2115). The spectroscopic data of this compound were also very similar to those of **7**, suggesting the same tricyclic meroterpenoid nature. However, detailed examination of its ^13^C and ^1^H NMR and HSQC data revealed noticeable differences in the signals of carbons and protons at the hydroxyl-bearing cyclohexanone moiety (ring A) (Table 2). Although ^1^H-^1^H COSY data showed the presence of the same linear assembly of a hydroxyl-methine and two methylene protons as **7**, the HMBC data revealed grossly different proton−carbon correlations. That is, the C-1 ketone (δ_C_ 199.9) was correlated with hydroxyl-methine (δ_H_ 4.07) and methylene (δ_H_ 2.24 and 1.83) protons. The latter methylene protons were additionally correlated to the C-6 (δ_C_ 105.6) and C-5 (δ_C_ 170.8) olefinic carbons. Therefore, **8** was structurally defined to be a regioisomer of **7** bearing a hydroxyl group at C-2.

**Figure 8:**
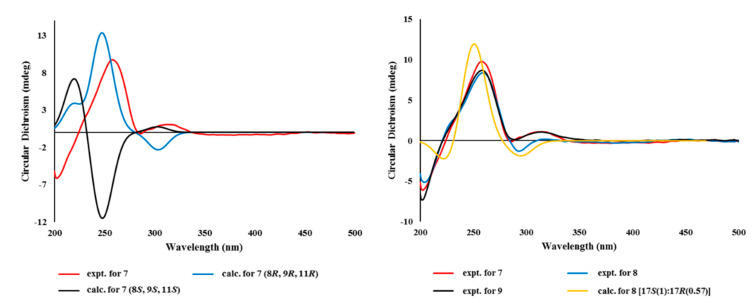
Experimental and calculated ECD spectra of compounds **7**–**9**.

The relative and absolute configurations of **8** were pursued stepwise as for **7**. First, the NOESY data of this compound showed identical proton–proton spatial proximities to **7**, revealing the same relative configurations at rings B and C. Then, the absolute configuration at C-2 was also assigned as *R* by modified Mosher’s method (Figure 6). Strikingly, however, the ^1^H NMR spectra of PGME-amides of **8** from treatments with (*S*)- and (*R*)-PGME were virtually identical to each other and consisted of pairing proton signals. A detailed examination indeed revealed that **5** was a mixture of two 17-epimers with a ratio of 1:0.57. Based upon the proton intensity, the PGME analysis also concluded the 17*S* and 17*R* configuration for the major and minor constituents, respectively. The analytical and spectroscopic behaviors of 8 as not an epimeric mixture but a single compound would be attributed from the far remote location of C-17 from other stereogenic centers. Having this, the absolute configurations of **8** were also assigned by ECD measurements. As shown in Figure 8, the measured ECD profile of **8** was very similar to that of **7**, defining the same absolute configurations for the ring portion between these compounds. This interpretation was firmly supported by the calculated ECD data of **8** with fixed absolute configurations and epimeric ratio (1:0.57), which were almost identical to the measured one. Thus, the structure of **8**, designated as tricycloalterfurene F, was determined to be an epimeric mixture of tricyclic meroterpene carboxylic acids.

The molecular formula of tricycloalterfurene G (**9**) was established as C_22_H_32_O_6_ by HRFABMS analysis ([M + Na]^+^ *m*/*z* 415.2104, calcd for C_22_H_32_O_6_Na, 415.2091). The ^13^C and ^1^H NMR data of this compound were very similar to those of **7**, with appearance of a methoxy group (δ_C_/δ_H_ 52.1/3.65) as the most noticeable difference (Table 2). This methyl group was placed at the C-18 carboxylic group by combined 2D NMR data, including crucial HMBC correlation between the methoxy proton and carboxylic carbon (δ_C_ 178.9). After deducing the same NOESY-based relative configurations as **7**, the experimental ECD profiles of **9** were also very similar to those of **7,** assigning the same absolute configurations between these (Figure 8). Thus, the structure of tricycloalterfurene G (**9**) was defined to be an ester derivative of tricycloalterfurene E (**7**).

The obtained bianthraquinones (**1**–**6**) and meroterpenoids (**7**–**9**) were assayed for their cytotoxicity. However, all of these were inactive (IC_50_ » 20 μM) against a number of human cancer cell-lines: A549 (lung cancer), HCT116 (colon cancer), MDA-MB-231 (breast cancer), PC3 (prostate cancer), SK-Hep1 (liver cancer), and SNU638 (stomach cancer). In addition, these compounds were inactive (MIC > 128 μg/mL) against diverse human pathogenic bacterial strains *Enterococcus faecalis* (ATCC19433), *Enterococcus faecium* (ATCC19434), *Klebsiella pneumoniae* (ATCC10031), *Proteus hauseri* (NBRC3851), *Salmonella enterica* (ATCC14028), and *Staphylococcus arueus* (ATCC6538p). These compounds were also inactive (IC_50_ > 145 μM) against microbial key enzymes sortase A (SrtA) and isocitrate lyase (ICL).

**Figure 9:**
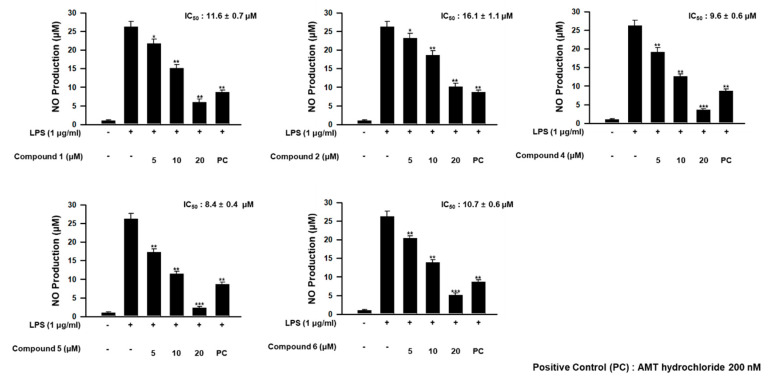
Concentration-dependent effect of **1**, **2**, and **4**–**6** on lipopolysaccharide (LPS)-stimulated NO production in RAW 264.7 cells. * *p* < 0.05, ** *p* < 0.01, and *** *p* < 0.001 indicate significant differences relative to the vehicle-treated control group.

**Figure 10:**
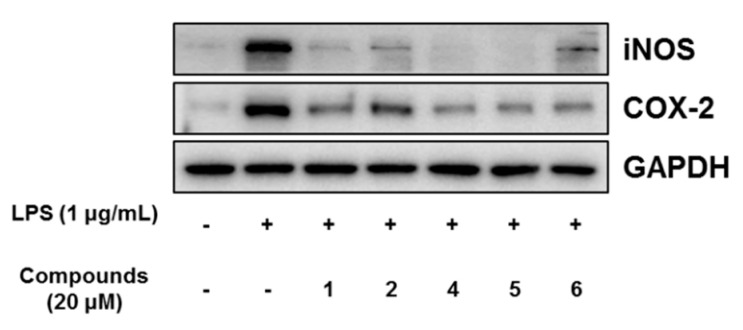
Effect of compounds **1**, **2**, and **4**–**6** on LPS-induced protein expressions of iNOS and COX-2 in RAW 264.7 cells. The cells were treated with 20 μM of compounds for 30 min prior to LPS (1 μg/mL) treatment and incubated for 18 h. Protein expression levels were determined by Western blotting analysis. Glyceraldehyde-3-phosphate dehydrogenase (GAPDH) was used as the internal standard.

In our further assay using bianthraquinones, the anti-inflammatory activities were indirectly evaluated by ability to suppress lipopolysaccharide (LPS)-induced nitric oxide (NO) production in RAW 264.7 mouse macrophages. As shown in Figure 9, compounds **1**, **2**, and **4**–**6** showed moderate anti-inflammatory activity with IC_50_ values of 11.6 ± 0.7, 16.1 ± 1.1, 9.6 ± 1.6, 8.4 ± 0.4, and 10.7 ± 0.6 μM, respectively, while 3 was inactive. No measurable cytotoxicity was observed against mouse macrophages at these concentrations (Supplementary Materials, Figure S55). To further elucidate the inhibitory mechanism on NO production, the effect of compounds on the protein expression levels of iNOS and COX-2, the key inflammatory mediators, was evaluated. As shown in Figure 10, protein levels of iNOS and COX-2 in RAW 264.7 cells incubated with LPS for 18 h increased in comparison to quiescent cells, but this was significantly down-regulated by treatment of the compounds (20 μM) 30 min prior to LPS exposure. These findings showed that the bianthraquinones would be potential candidates for the anti-inflammatory related study.

## 3. Materials and Methods

### 3.1. General Experimental Procedures

Optical rotations were measured on a JASCO P1020 polarimeter (Jasco, Tokyo, Japan) using a 1 cm cell. UV spectra were acquired with a Hitachi U-3010 spectrophotometer (Hitachi High-Technologies, Tokyo, Japan). ECD spectra were recorded on an Applied Photophysics Chirascan plus CD spectrometer. IR spectra were recorded on a JASCO 4200 FT-IR spectrometer (Jasco, Tokyo, Japan) using a ZnSe cell. ^1^H and ^13^C NMR spectra were measured in CD_3_OD and THF-*d_8_* solutions on Bruker Avance -500, -800 instruments (Billerica, MA, USA) and JEOL -400, -600 instruments (Peabody, MA, USA). High resolution FAB mass spectrometric data were obtained at the Korea Basic Science Institute (Daegu, Korea), and were acquired using a JEOL JMS 700 mass spectrometer (Jeol, Tokyo, Japan) with *meta*-nitrobenzyl alcohol (NBA) as the matrix. Semi-preparative HPLC separations were performed on a Spectrasystem p2000 equipped with a Spectrasystem RI-150 refractive index detector. All solvents used were spectroscopic grade or distilled from glass prior to use.

### 3.2. Fungal Material

The fungal strain *Stemphylium* sp. (strain number FJJ006) was isolated from an unidentified sponge collected using SCUBA at a depth of 30 m off the coast of Jeju-do (Island), Korea, on 29 September 2014. The sponge specimen was macerated and diluted using sterile seawater. One milliliter of the diluted sample was processed utilizing the spread plate method in YPG agar media (5 g of yeast extract, 5 g of peptone, 10 g of glucose, 0.15 g of penicillin G, 0.15 g of streptomycin sulfate, 24.8 g of Instant Ocean, and 16 g of agar in 1 L of distilled water) plates. The plates were incubated at 28 °C for 5 days. The strain was identified using standard molecular biology protocols by DNA amplification and sequencing of the ITS region. Genomic DNA extraction was performed using Intron’s i-genomic BYF DNA Extraction Mini Kit according to the manufacturer’s protocol. The nucleotide sequence of FJJ006 was deposited in the GenBank database under accession number KU519425. The 18S rDNA sequence of this strain exhibited 99% identity (577/579) with that of *Stemphylium* sp. PNYZ13070801 (GenBank accession number KJ481209).

### 3.3. Fermentation

The fungal strain was cultured on a solid YPG medium (5 g of yeast extract, 5 g of peptone, 10 g of glucose, 24.8 g of Instant Ocean, and 16 g of agar in 1 L of distilled water) for 7 days. An agar plug (1 × 1 cm) was inoculated for 7 days in a 250 mL flask that contained 100 mL of YPG medium. Then, 10 mL of each culture was transferred to a 2.8 L Fernbach flask containing semi-solid rice medium (200 g of rice, 0.5 g of yeast extract, 0.5 g of peptone, and 12.4 g of Instant Ocean in 500 mL of distilled water). In total, 2000 g of rice media were prepared and cultivated for 35 days at 28 °C, agitating once every week.

### 3.4. Extraction and Isolation

The entire culture was macerated and extracted with MeOH (1 L × 3 for each flask). The solvent was evaporated in vacuo to afford a brown organic extract (5.8 g). The extract was separated by C_18_ reversed-phase vacuum flash chromatography using sequential mixtures of H_2_O and MeOH (seven fractions of H_2_O-MeOH, gradient from 60:40 to 0:100), acetone, and finally, EtOAc as the eluents. Based on the results of ^1^H NMR analysis, the fractions eluted with H_2_O-MeOH 50:50 (550 mg), 40:60 (300 mg), and 20:80 (670 mg) were chosen for further separation. The fraction that eluted with H_2_O-MeOH (50:50) was separated by semi-preparative reversed-phase HPLC (YMC-ODS-A column, 250 × 10 mm, 5 μm; gradient from H_2_O-MeCN (90:10) to (50:50), 1.8 mL/min), to yield compound **9** (*t*_R_ = 52.6 min). The H_2_O-MeOH (40:60) fraction from vacuum flash chromatography was separated by semi-preparative reversed-phase HPLC (H_2_O-MeCN, (80:20) to (30:70), 2.0 mL/min) to afford, in the order of elution, compounds **1** (*t*_R_ = 44.8 min), **2** (*t*_R_ = 45.4 min), **4** (*t*_R_ = 50.8 min), **6** (*t*_R_ = 51.4 min), **7** (*t*_R_ = 59.4 min), and **8** (*t*_R_ = 61.3 min). The H_2_O-MeOH (20:80) fraction from vacuum flash chromatography was separated by semi-preparative reversed-phase HPLC (H_2_O-MeCN, (65:35) to (25:75), 2.0 mL/min), affording compounds **3** (*t*_R_ = 51.2 min) and **5** (*t*_R_ = 40.6 min). The overall isolated amounts of 1–9 were 7.0, 3.0, 0.7, 3.0, 3.2, 3.2, 6.5, 8.2, and 2.8 mg, respectively.

Alterporriol Z1 (**1**): orange amorphous solid, ${\left[\alpha \right]}_{25}^{D}$ +1.8 (*c* 0.10, MeOH); UV (MeOH) *λ*_max_ (log ε) 223 (3.97), 275 (3.86), 430 (3.36) nm; ECD (*c* 1.62 × 10^−4^ M, MeOH) *λ*_max_ (Δ*ε*), 220 (−4.02), 229 (−5.06), 269 (35.79), 285 (−36.06), 317 (−7.91) nm; IR (ZnSe) *ν*_max_ 3544, 2970, 1622, 1593, 1372 cm^−1^; ^1^H and ^13^C NMR data, see Table 1; HRFABMS *m*/*z* 619.1449 [M + H]^+^ (calcd for C_32_H_27_O_13_, 619.1446).

Alterporriol Z2 (**2**): orange amorphous solid, ${\left[\alpha \right]}_{25}^{D}$ −2.5 (*c* 0.10, MeOH); UV (MeOH) *λ*_max_ (log ε) 227 (3.79), 274 (3.68), 432 (3.09) nm; ECD (*c* 1.62 × 10^−4^ M, MeOH) *λ*_max_ (Δ*ε*), 228 (6.18), 267 (−18.63), 284 (12.77), 317 (3.02) nm; IR (ZnSe) *ν*_max_ 3412, 2973, 2938, 1638, 1580, 1397, 1293 cm^−1^; ^1^H and ^13^C NMR data, see Table 1; HRFABMS *m*/*z* 619.1454 [M + H]^+^ (calcd for C_32_H_27_O_13_, 619.1446).

Alterporriol Z3 (**3**): dark red amorphous solid, ${\left[\alpha \right]}_{25}^{D}$ +13.5 (*c* 0.20, MeOH); UV (MeOH) *λ*_max_ (log ε) 212 (3.83), 278 (3.70), 420 (3.27) nm; ECD (*c* 1.58 × 10^−4^ M, MeOH) *λ*_max_ (Δ*ε*), 218 (−18.94), 235 (8.44), 263 (15.31), 287 (−13.9) nm; IR (ZnSe) *ν*_max_ 3416, 3120, 2973, 2931, 1638, 1605, 1322 cm^−1^; ^1^H and ^13^C NMR data, see Table 1; HRFABMS *m*/*z* 655.1430 [M + Na]^+^ (calcd for C_33_H_28_O_13_Na, 655.1422).

Tricycloalterfurene E (**7**): brown amorphous solid, ${\left[\alpha \right]}_{25}^{D}$ +88.2 (*c* 0.20, MeOH); UV (MeOH) *λ*_max_ (log ε) 220 (3.53), 262 (3.48) nm; ECD (*c* 2.65 × 10^−4^ M, MeOH) *λ*_max_ (Δ*ε*), 258 (9.73), 313 (1.06) nm; IR (ZnSe) *ν*_max_ 3546, 3347, 2930, 2854, 1748, 1610, 1538, 1371 cm^−1^; ^1^H and ^13^C NMR data, see Table 2; HRFABMS *m*/*z* 379.2118 [M + H]^+^ (calcd for C_21_H_31_O_6_, 379.2115).

Tricycloalterfurene F (**8**): brown amorphous solid, ${\left[\alpha \right]}_{25}^{D}$ +74.0 (*c* 0.20, MeOH); UV (MeOH) *λ*_max_ (log ε) 225 (3.42), 258 (3.35) nm; ECD (*c* 2.65 × 10^−4^ M, MeOH) *λ*_max_ (Δ*ε*), 259 (8.39), 315 (0.17) nm; IR (ZnSe) *ν*_max_ 3593, 3350, 2928, 2860, 1757, 1674, 1617, 1514, 1221 cm^−1^; ^1^H and ^13^C NMR data, see Table 2; HRFABMS *m*/*z* 379.2120 [M + H]^+^ (calcd for C_21_H_31_O_6_, 379.2115).

Tricycloalterfurene G (**9**): brown amorphous solid, ${\left[\alpha \right]}_{25}^{D}$ +58.4 (*c* 0.20, MeOH); UV (MeOH) *λ*_max_ (log ε) 211 (3.49) nm; ECD (*c* 2.55 × 10^−4^ M, MeOH) *λ*_max_ (Δ*ε*), 258 (8.67), 315 (1.07) nm; IR (ZnSe) *ν*_max_ 3603, 3356, 2933, 2860, 1742, 1602, 1536, 1371 cm^−1^; ^1^H and ^13^C NMR data, see Table 2; HRFABMS *m*/*z* 415.2104 [M + Na]^+^ (calcd for C_22_H_32_O_6_Na, 415.2091).

### 3.5. Preparations of the (S)- and (R)-MTPA Esters of Compounds **7** and **8**

To a solution of compound **7** (0.6 mg, 1 μM) in dry pyridine (500 μL), (*S*)-MTPA chloride (10 μL, 5.2 μM) and DMAP (0.5 mg) were successively added. After stirring for 3 h at 40 °C under N_2_, the reaction mixture was concentrated under reduced pressure, and the residue was purified by reversed-phase HPLC (YMC-ODS column, 4.6 × 250 mm; H_2_O-MeCN, 73:27) to give **7**-4*R* (0.3 mg), the (*R*)-MTPA ester of **7**. Compound **7**-4*S* (0.3 mg), the (*S*)-MTPA ester of **7**, was prepared from (*R*)-MTPA chloride in a similar fashion. Compounds **8**-2*S* and **8**-2*R* (0.3 mg each), the (*S*)- and (*R*)-MTPA esters of **5**, respectively, were also prepared using this method.

#### 3.5.1. (*S*)-MTPA Ester of **7** (**7**-4*S*)

White amorphous solid; ^1^H NMR (CD_3_OD, 800 MHz) *δ*_H_ 7.574–7.566 (2H, m, MTPA-Ar), 7.457–7.435 (3H, m, MTPA-Ar), 5.864 (1H, dd, *J* = 6.5, 5.0 Hz, H-4), 5.227 (1H, dq, *J* = 8.7, 1.1 Hz, H-12), 4.789 (1H, td, *J* = 9.1, 3.8 Hz, H-11), 3.903 (1H, dd, *J* = 4.6, 1.5 Hz, H-8), 3.633 (3H, s, H-22), 3.574 (3H, s, MTPA-OMe), 2.591 (1H, d, *J* = 18.2 Hz, H-7*α*), 2.512 (1H, dd, *J* = 8.2, 5.2 Hz, H-2*α*), 2.488 (1H, m, H-2*β*), 2.474 (1H, m, H-3*α*), 2.371 (1H, m, H-17), 2.346 (1H, m, H-10*α*), 2.238 (1H, m, H-3*β*), 2.192 (1H, ddd, *J* = 18.1, 4.5, 1.0 Hz, H-7*β*), 2.022 (2H, m, H-14), 1.945 (1H, dd, *J* = 13.7, 3.7 Hz, H-10*β*), 1.654 (3H, d, *J* = 1.1 Hz, H-20), 1.612 (1H, m, H-16*α*), 1.428 (2H, m, H-15), 1.411 (1H, m, H-16*β*), 1.122 (3H, d, *J* = 7.0 Hz, H-21), 1.015 (3H, s, H-19); LRESIMS *m*/*z* 609.2 [M + H]^+^.

#### 3.5.2. (*R*)-MTPA Ester of **7** (**7**-4*R*)

White amorphous solid; ^1^H NMR (CD_3_OD, 800 MHz) *δ*_H_ 7.575–7.567 (2H, m, MTPA-Ar), 7.459–7.431 (3H, m, MTPA-Ar), 5.804 (1H, t, *J* = 4.9 Hz, H-4), 5.231 (1H, dq, *J* = 8.7, 1.0 Hz, H-12), 4.819 (1H, m, H-11), 3.979 (1H, dd, *J* = 4.5, 1.4 Hz, H-8), 3.633 (3H, s, H-22), 3.579 (3H, s, MTPA-OMe), 2.619 (1H, d, *J* = 18.3 Hz, H-7*α*), 2.463 (1H, m, H-17), 2.443 (1H, m, H-10*α*), 2.371 (1H, dd, *J* = 6.2, 5.0 Hz, H-2*α*), 2.343 (1H, dd, *J* = 8.9, 4.9 Hz, H-2*β*), 2.300 (1H, m, H-3*α*), 2.253 (1H, dd, *J* = 18.6, 4.3 Hz, H-7*β*), 2.149 (1H, m, H-3*β*), 2.029 (1H, dd, *J* = 13.7, 3.9 Hz, H-10*β*), 2.003 (2H, m, H-14), 1.654 (3H, d, *J* = 1.0 Hz, H-20), 1.603 (1H, m, H-16*α*), 1.414 (2H, m, H-15), 1.402 (1H, m, H-16*β*), 1.295 (3H, s, H-19), 1.109 (3H, d, *J* = 7.0 Hz, H-21); LRESIMS *m*/*z* 609.2 [M + H]^+^.

#### 3.5.3. (*S*)-MTPA Ester of **8** (**8**-2*S*)

White amorphous solid; ^1^H NMR (CD_3_OD, 800 MHz) *δ*_H_ 7.680–7.668 (2H, m, MTPA-Ar), 7.428–7.416 (3H, m, MTPA-Ar), 5.581 (1H, dd, *J* = 13.1, 5.3 Hz, H-2), 5.149 (1H, dq, *J* = 8.6, 1.1 Hz, H-12), 4.798 (1H, td, *J* = 9.0, 3.9 Hz, H-11), 3.966 (1H, dd, *J* = 4.6, 1.3 Hz, H-8), 3.615 (3H, s, H-22), 3.551 (3H, s, MTPA-OMe), 2.742 (1H, t, *J* = 14.8 Hz, H-4*α*), 2.655 (1H, dd, *J* = 17.8, 1.1 Hz, H-7*α*), 2.526 (1H, dd, *J* = 17.2, 4.1 Hz, H-4*β*), 2.441 (1H, m, H-17), 2.413 (1H, m, H-10*α*), 2.316 (1H, m, H-3*α*), 2.232 (1H, m, H-7*β*), 2.209 (1H, m, H-3*β*), 2.023 (1H, dd, *J* = 13.7, 3.9 Hz, H-10*β*), 1.971 (2H, m, H-14), 1.636 (3H, s, H-20), 1.571 (1H, m, H-16*α*), 1.381 (2H, m, H-15), 1.372 (1H, m, H-16*β*), 1.339 (3H, s, H-19), 1.095 (3H, d, *J* = 7.0 Hz, H-21); LRESIMS *m*/*z* 609.2 [M + H]^+^.

#### 3.5.4. (*R*)-MTPA Ester of **8** (**8**-2*R*)

White amorphous solid; ^1^H NMR (CD_3_OD, 800 MHz) *δ*_H_ 7.671–7.659 (2H, m, MTPA-Ar), 7.422–7.408 (3H, m, MTPA-Ar), 5.611 (1H, dd, *J* = 12.8, 5.3 Hz, H-2), 5.133 (1H, br d, *J* = 8.6 Hz, H-12), 4.800 (1H, td, *J* = 9.0, 3.9 Hz, H-11), 3.973 (1H, d, *J* = 4.5 Hz, H-8), 3.655 (3H, s, MTPA-OMe), 3.603 (3H, s, H-22), 2.705 (1H, m, H-4*α*), 2.682 (1H, dd, *J* = 17.9, 1.2 Hz, H-7*α*), 2.480 (1H, d, *J* = 17.2 Hz, H-4*β*), 2.428 (1H, m, H-17), 2.409 (1H, m, H-10*α*), 2.243 (1H, m, H-7*β*), 2.165 (1H, m, H-3*α*), 2.067 (1H, dd, *J* = 12.4, 5.3 Hz, H-3*β*), 2.017 (1H, dd, *J* = 13.7, 3.9 Hz, H-10*β*), 1.956 (2H, m, H-14), 1.634 (3H, s, H-20), 1.555 (1H, m, H-16*α*), 1.368 (2H, m, H-15), 1.359 (1H, m, H-16*β*), 1.340 (3H, s, H-19), 1.082 (3H, d, *J* = 7.0 Hz, H-21); LRESIMS *m*/*z* 609.2 [M + H]^+^.

### 3.6. Preparations of the (S)- and (R)-PGME Amides of Compounds ***7*** and ***8***

To a dry DMF solution (500 μL) of compound **7** (0.6 mg, 1.6 μM) and (*S*)-PGME (1.4 mg, 7.4 μM), PyBOP (3.8mg, 7.4 μM), HOBT (1.0mg, 7.4 μM), and *N*-methylmorpholine (100 μL) were added. After stirring the mixture for 3 h at room temp, a 5% HCl solution and EtOAc were added to the reaction mixture. The EtOAc layer was subsequently washed with saturated NaHCO_3_ solution and brine. The organic layer was dried over anhydrous Na_2_SO_4_. After removing the solvent under vacuum, the residue was purified by reversed-phase HPLC (YMC-ODS column, 4.6 × 250 mm; H_2_O-MeOH, 35:65) to give (*S*)-PGME amide **7**-18*S* (0.3 mg). Compound **7**-18*R* (0.4 mg), the (*R*)-PGME amide of **7**, was prepared from (*R*)-PGME in a similar fashion. Compounds **8**-18***S*** and **8**-18***R*** (0.3 mg each), the (*S*)- and (*R*)-PGME amides of 5, respectively, were also prepared using this method.

#### 3.6.1. (*S*)-PGME Amide of **7** (**7**-18*S*)

Brown amorphous solid; ^1^H NMR (CD_3_OD, 800 MHz) *δ*_H_ 7.371–7.329 (5H, m, PGME-Ar), 5.427 (1H, s, PGME-H-1), 5.202 (1H, dq, *J* = 8.6, 1.1 Hz, H-12), 4.802 (1H, td, *J* = 9.0, 4.1 Hz, H-11), 4.299 (1H, t, *J* = 5.1 Hz, H-4), 3.945 (1H, dd, *J* = 4.6, 1.6 Hz, H-8), 3.681 (3H, s, PGME-OMe), 2.612 (1H, m, H-2*α*), 2.592 (1H, d, *J* = 17.8 Hz H-7*α*), 2.456 (1H, m, H-17), 2.419 (1H, dd, *J* = 13.7, 9.4 Hz, H-10*α*), 2.309 (1H, ddd, *J* = 16.9, 6.9, 4.8 Hz, H-2*β*), 2.230 (1H, dd, *J* = 18.0, 4.6 Hz, H-7*β*), 2.169 (1H, m, H-3*α*), 2.090 (1H, dd, *J* = 13.7, 4.1 Hz, H-10*β*), 2.005 (2H, m, H-14), 1.964 (1H, m, H-3*β*), 1.669 (3H, d, *J* = 1.2 Hz, H-20), 1.589 (1H, m, H-16*α*), 1.454 (2H, m, H-15), 1.354 (3H, s, H-19), 1.332 (1H, m, H-16*β*), 1.049 (3H, d, *J* = 6.9 Hz, H-21); LRESIMS *m*/*z* 526.3 [M + H]^+^.

#### 3.6.2. (*R*)-PGME Amide of **7** (**7**-18*R*)

Brown amorphous solid; ^1^H NMR (CD_3_OD, 800 MHz) *δ*_H_ 7.361–7.318 (5 H, m, PGME-Ar), 5.466 (1H, s, PGME-H-1), 5.126 (1H, dq, *J* = 8.6, 1.1 Hz, H-12), 4.763 (1H, td, *J* = 9.1, 4.2 Hz, H-11), 4.253 (1H, t, *J* = 5.1 Hz, H-4), 3.933 (1H, dd, *J* = 4.6, 1.6 Hz, H-8), 3.697 (3H, s, PGME-OMe), 2.603 (1H, m, H-2*α*), 2.587 (1H, d, *J* = 17.6 Hz H-7*α*), 2.460 (1H, m, H-17), 2.393 (1H, dd, *J* = 13.7, 9.4 Hz, H-10*α*), 2.284 (1H, ddd, *J* = 16.9, 6.9, 4.9 Hz, H-2*β*), 2.224 (1H, dd, *J* = 18.0, 4.7 Hz, H-7*β*), 2.151 (1H, m, H-3*α*), 2.044 (1H, dd, *J* = 13.7, 4.1 Hz, H-10*β*), 1.957 (1H, m, H-3*β*), 1.918 (2H, m, H-14), 1.576 (3H, d, *J* = 1.2 Hz, H-20), 1.532 (1H, m, H-16*α*), 1.345 (3H, s, H-19), 1.321 (1H, m, H-16*β*), 1.288 (2H, m, H-15), 1.108 (3H, d, *J* = 6.9 Hz, H-21); LRESIMS *m*/*z* 526.3 [M + H]^+^.

#### 3.6.3. (*S*)-PGME Amide of **8** (**8**-18*S*)

Major isomer; brown amorphous solid; ^1^H NMR (CD_3_OD, 800 MHz) *δ*_H_ 7.373–7.331 (5H, m, PGME-Ar), 5.429 (1H, s, PGME-H-1), 5.200 (1H, br d, *J* = 8.4 Hz, H-12), 4.799 (1H, td, *J* = 9.0, 4.1 Hz, H-11), 4.067 (1H, dd, *J* = 12.7, 5.2 Hz, H-2), 3.944 (1H, dd, *J* = 4.8, 1.3 Hz, H-8), 3.683 (3H, s, PGME-OMe), 2.674 (1H, d, *J* = 17.8 Hz H-7*α*), 2.572 (1H, m, H-4*α*), 2.465 (1H, m, H-17), 2.446 (1H, m, H-4*β*), 2.396 (1H, m, H-10*α*), 2.238 (1H, m, H-3*α*), 2.207 (1H, m, H-7*β*), 2.026 (1H, m, H-10*β*), 2.007 (2H, m, H-14), 1.823 (1H, m, H-3*β*), 1.668 (3H, d, *J* = 0.9 Hz, H-20), 1.596 (1H, m, H-16*α*), 1.462 (2H, m, H-15), 1.341 (1H, m, H-16*β*), 1.308 (3H, s, H-19), 1.048 (3H, d, *J* = 6.8 Hz, H-21); LRESIMS *m*/*z* 526.3 [M + H]^+^.

#### 3.6.4. (*R*)-PGME Amide of **8** (**8**-18*R*)

Major isomer; brown amorphous solid; ^1^H NMR (CD_3_OD, 800 MHz) *δ*_H_ 7.367–7.324 (5H, m, PGME-Ar), 5.473 (1H, s, PGME-H-1), 5.111 (1H, br d, *J* = 8.7 Hz, H-12), 4.759 (1H, td, *J* = 9.0, 4.0 Hz, H-11), 4.060 (1H, dd, *J* = 12.4, 5.2 Hz, H-2), 3.933 (1H, dd, *J* = 4.8, 1.0 Hz, H-8), 3.695 (3H, s, PGME-OMe), 2.667 (1H, d, *J* = 18.0 Hz H-7*α*), 2.566 (1H, m, H-4*α*), 2.462 (1H, m, H-17), 2.445 (1H, m, H-4*β*), 2.370 (1H, m, H-10*α*), 2.232 (1H, m, H-3*α*), 2.206 (1H, m, H-7*β*), 1.975 (1H, m, H-10*β*), 1.919 (2H, m, H-14), 1.815 (1H, m, H-3*β*), 1.574 (3H, d, *J* = 0.5 Hz, H-20), 1.546 (1H, m, H-16*α*), 1.328 (1H, m, H-16*β*), 1.304 (2H, m, H-15), 1.300 (3H, s, H-19), 1.108 (3H, d, *J* = 6.8 Hz, H-21); LRESIMS *m*/*z* 526.3 [M + H]^+^.

### 3.7. ECD Calculations

The ground-state geometries were optimized with density functional theory (DFT) calculations, using Turbomole with the basis set def-SVP for all atoms and the functional B3-LYP. The ground states were further confirmed by the harmonic frequency calculation. The calculated ECD data corresponding to the optimized structures were obtained with the time-dependent density-functional theory (TD-DFT) at the B3-LYP functional. The ECD spectra were simulated by overlapping Gaussian functions for each transition, where *σ* is the width of the band at 1/*e* height. Values Δ*E_i_* and *R_i_* were the excitation energies and rotatory strengths, respectively, for transition *i*. In the current work, the value was 0.10 eV.

(1) $$∆\in \left(E\right)=\frac{1}{2.297\times 10^{-39}}\frac{1}{\sqrt{2\pi \sigma }}\overset{A}{\underset{i}{\sum }}∆E_{i}R_{i}e^{[-{\left(E-∆E_{i}\right)}^{2}/\left(2\sigma {)}^{2}\right]}$$

### 3.8. DP4 Analysis

Conformational searches were performed using MacroModel software (Version 9.9, Schrödinger LLC, New York, NY, USA) interfaced in Maestro (Version 9.9, Schrödinger LLC) with a mixed torsional/low-mode sampling method. Conformers within 10 kJ/mol found in the MMFF force field were regarded and the geometry of the conformers was optimized at the B3-LYP/6-31G++ level in the gas phase. Ground state geometry optimization of each conformer was carried out by density functional theory (DFT) modeling with TurbomoleX 4.3.2 software. The basis set for the calculation was def-SVP for all atoms, and the level of theory was B3-LYP at the functional level in the gas phase. The calculated chemical shift values were averaged by the Boltzmann populations and utilized for DP4 analysis.

### 3.9. Cytotoxic, Antibacterial, and Enzyme-Inhibitory Activities Assays

Cytotoxicity assay was performed in accordance with the published protocols [26]. The antimicrobial and SrtA and ICL-inhibitory assays were performed according to previously described methods [27,28,29].

### 3.10. RAW 264.7 Cell Culture

A RAW 264.7 murine macrophage cell line was purchased from American Type Cell Culture (Rockville, MD, USA). Cells were cultured in DMEM with 10% fetal bovine serum and antibiotic−antimycotics (i.e., penicillin G sodium, 100 units/mL; streptomycin, 100 μg/mL; amphotericin B, 250 ng/mL) at 37 °C in a humidified incubator with 5% carbon dioxide. All reagents used for cell culture were purchased from Gibco^®^ Invitrogen Corp. (Grand Island, NY, USA).

### 3.11. Nitrite Production Measurement

The concentration of nitrite in the cultured media was used as a measure of NO production. The assay was performed as previously described [30]. RAW 264.7 cells were plated at a density of 5 × 10^5^ per well in a 24-well culture plate and incubated in a 37 °C humidified incubator with 5% CO_2_ in air for 18 h. The incubated cells were pretreated with phenol-red-free medium containing various concentrations of tested compounds for 30 min, followed by 1 μg/mL of LPS treatment for 18 h more. Aliquots of the supernatant from each well (100 μL) were transferred to a 96-well plate, and nitrite concentration was measured using Griess reagent. After the Griess reaction, MTT solution (final concentration of 500 μg/mL) was added to each well and further incubated for 4 h at 37 °C. Each medium was discarded, and dimethyl sulfoxide was added to each well to dissolve generated formazan. The absorbance was measured at 570 nm, and percent survival was determined by comparison with LPS treated control group. All reagents used in the nitrite production measurement were purchased from Sigma-Aldrich (St. Louis, MO, USA).

### 3.12. Western Blotting Analysis

The Western blotting analysis was performed as previously described [31]. Briefly, the samples with cell lysates were boiled for 10 min at 100 °C. The concentrations of proteins in the cell lysates were quantified using the bicinchoninic acid method. Equal amounts of protein were subjected to 8%–10% sodium dodecyl sulfate-polyacrylamide gel electrophoresis and transferred to polyvinylidene fluoride membranes (Millipore, Bedford, MA, USA) activated with 100% methanol. The membranes were blocked using 5% BSA in a mixture of Tris-buffered saline and Tween 20 (1×), and subsequently probed with the following antibodies: anti-iNOS, COX-2, and GAPDH (Cell Signaling Technology, Beverly, MA, USA). The blots were visualized using an enhanced chemiluminescence detection kit (Intron Biotechnology, Sungnam, Korea) and an ImageQuant LAS-4000 Imager (Fujifilm Corp., Tokyo, Japan).

## 4. Conclusions

Three new bianthraquinones, alterporriol Z1–Z3 (1–3), along with three known compounds (4–6) of the same structural class, were isolated from the culture broth of a marine-derived *Stemphylium* sp. fungus. Based upon the results of combined spectroscopic analyses and ECD measurements, the structures of new compounds were determined to be the 6-6′- (1 and 2) and 1-5′- (3) C-C connected *pseudo*-dimeric anthraquinones, respectively. The absolute configuration of the chiral axis of 4 was also assigned similarly. In addition, three new meroterpenoids, tricycloalterfurenes E–G (7–9), isolated from the same fungal culture broth, were structurally elucidated by combined spectroscopic methods. The relative and absolute configurations of these meroterpenoids were determined by modified Mosher’s, PGME, and computational methods. Although none of these compounds were active against cytotoxic and antibacterial assays, the bianthraquinones (except 3) exhibited significantly inhibited nitric oxide (NO) production and suppressed the expression of iNOS and COX-2 in LPS-stimulated RAW 264.7 cells.

## Figures and Tables

**Table 1:** ^13^C and ^1^H NMR data of compounds **1**–**3** in CD_3_OD.

No.	1 *^a^*		2 *^b^*		3 *^c^*	
	*δ*_C_, Type	*δ*_H_ (*J* in Hz)	*δ*_C_, Type	*δ*_H_ (*J* in Hz)	*δ*_C_, Type	*δ*_H_ (*J* in Hz)
1	111.7, CH	7.51, d (0.5)	111.8, CH	7.51, d (0.5)	129.0, C	
2	164.0, C		163.7, C		165.5, C *^d^*	
3	133.8, C		133.8, C		134.5, C	
4	131.2, CH	7.65, d (0.5)	131.3, CH	7.65, d (0.5)	130.5, CH	8.00, s
4a	126.8, C		127.1, C		131.2, C	
5	165.8, C		166.2, C		106.9, CH	7.20, d (2.5)
6	125.3, C		125.7, C		167.1, C	
7	104.6, CH	6.78, s	104.3, CH	6.80, s	105.9, CH	6.52, d (2.5)
8	166.9, C		167.1, C		166.0, C	
8a	111.9, C		111.7, C		112.1, C	
9	188.7, C		188.6, C		191.0, C	
9a	134.7, C		134.6, C		132.8, C	
10	183.5, C		183.5, C		182.3, C	
10a	133.4, C		132.8, C		137.8, C	
11	16.6, CH_3_	2.23, s	16.6, CH_3_	2.23, s	17.9, CH_3_	2.21, s
12	56.9, CH_3_	3.69, s	56.9, CH_3_	3.70, s	56.3, CH_3_	3.89, s
1′	70.6, CH	4.73, d (7.5)	70.6, CH	4.73, d (7.4)	75.2, CH	4.58, d (4.7)
2′	75.2, CH	3.79, d (7.5)	75.2, CH	3.76, d (7.4)	70.7, CH	3.93, d (4.7)
3′	74.6, C		74.7, C		75.4, C	
4′	70.1, CH	4.26, s	70.2, CH	4.26, s	70.6, CH	4.39, s
4a’	143.8, C		143.9, C		144.7, C	
5′	166.3, C		166.5, C		130.7, C	
6′	123.4, C		123.9, C		166.8, C	
7′	104.6, CH	6.81, s	104.6, CH	6.82, s	104.5, CH	6.79, s
8′	166.1, C		166.4, C		165.9, C	
8a’	111.0, C		111.0, C		110.9, C	
9′	190.5, C		190.6, C		189.6, C	
9a’	143.9, C		143.7, C		141.0, C	
10′	185.7, C		185.5, C		185.7, C	
10a’	130.8, C		130.7, C		n.d. *^e^*	
11′	22.3, CH_3_	1.33, s	22.3, CH_3_	1.32, s	21.6, CH_3_	1.33, s
12′	57.0, CH_3_	3.70, s	57.0, CH_3_	3.71, s	56.8, CH_3_	3.71, s
13′					62.8, CH_3_	3.77, s

*^a^*^–*c*^ Measured at 150/600, 100/400, and 200/800 MHz for ^13^C/^1^H NMR, respectively. *^d^* Assigned by HMBC data. *^e^* Not detected.

**Table 2:** ^13^C and ^1^H NMR Data of compounds **7**–**9** in CD_3_OD.

No.	7 *^a^*		8 *^a^*		9 *^b^*	
	δ_C_, type	δ_H_ (*J* in Hz)	δ_C_, type	δ_H_ (*J* in Hz)	δ_C_, type	δ_H_ (*J* in Hz)
1	200.4, C		199.9, C		200.4, C	
2	33.6, CH_2_	2.62, m 2.32, ddd (16.9, 6.9, 4.9)	72.4, CH	4.07, dd (12.6, 4.9)	33.6, CH_2_	2.62, m 2.32, ddd (17.0, 6.8, 5.1)
3	30.5, CH_2_	2.18, m 1.97, m	30.7, CH_2_	2.24, m1.83, m	30.5, CH_2_	2.19, m 1.97, m
4	66.8, CH	4.30, t (5.1)	28.8, CH_2_	2.58, m2.44, m	66.8, CH	4.29, t (5.3)
5	170.4, C		170.8, C		170.4, C	
6	107.5, C		105.6, C		107.5, C	
7	19.8, CH_2_	2.59, d (17.8) 2.22, dd (17.7, 4.2)	19.8, CH_2_	2.68, d (17.8) 2.20, dd (17.7, 4.2)	19.8, CH_2_	2.59, d (17.8) 2.22, dd (17.7, 4.2)
8	78.1, CH	3.95, dd (4.3, 1.2)	77.9, CH	3.95, dd (4.3, 1.2)	78.1, CH	3.95, dd (4.5, 1.3)
9	85.8, C		85.7, C		85.8, C	
10	46.8, CH_2_	2.42, dd (13.6, 9.4) 2.07, dd (13.6, 3.9)	46.9, CH_2_	2.39, m 2.00, dd (13.7, 3.7)	46.8, CH_2_	2.42, dd (13.7, 9.5) 2.07, dd (13.6, 3.9)
11	74.5, CH	4.80, td (9.0, 3.9)	74.5, CH	4.80, td (9.0, 3.9)	74.4, CH	4.79, m
12	127.5, CH	5.17, br d (8.8)	127.5, CH	5.17, br d (8.8)	127.7, CH	5.17, br d (8.5)
13	139.7, C		139.7, C		139.6, C	
14	40.2, CH_2_	1.99, m	40.3, CH_2_	1.98, m	40.1, CH_2_	1.98, m
15	26.3, CH_2_	1.42, m	26.5, CH_2_	1.42, m	26.1, CH_2_	1.39, m
16	34.5, CH_2_	1.59, m 1.38, m	34.9, CH_2_	1.58, m 1.35, m	34.4, CH_2_	1.58, m 1.38, m
17	40.8, CH	2.37, dq (13.8, 7.0)	41.6, CH	2.37, m	40.5, CH	2.46, m
18	180.9, C		182.1, C		178.9, C	
19	20.1, CH_3_	1.35, s	19.9, CH_3_	1.30, s	20.1, CH_3_	1.35, s
20	16.4, CH_3_	1.65, s	16.4, CH_3_	1.64, s	16.3, CH_3_	1.65, s
21	17.7, CH_3_	1.11, d (7.0)	18.0, CH_3_	1.11, d (7.0)	17.5, CH_3_	1.11, d (7.0)
22					52.1, OCH_3_	3.65, s

*^a^*^, *b*^ Measured at 100/800 and 100/500 MHz for ^13^C/^1^H, respectively.

## Supplementary Materials

The following are available online at https://www.mdpi.com/1660-3397/18/9/436/s1. Figures S1–S45: HRFABMS, 1D and 2D NMR spectra of 1–3 and 7–9, Figures S46–S49: ^1^H NMR spectrum of (*S*), (*R*)-MTPA Ester of 7–8, Figures S50-S53: ^1^H NMR spectrum of (*S*), (*R*)-PGME Amide of 7–8, Figure S54: The results of DP4 probability analysis of 1, Figure S55: The viability of RAW 264.7 cells was measured using the MTT assay, Table S1: Experimental (Exp.) and calculated (Cal.) chemical shift values of enantiomers A and B on aliphatic ring part of 1, Table S2: ^1^H NMR Data of 1 in THF-*d_8_*.
