# Supplementary material for: Bioactive Bianthraquinones and Meroterpenoids from a Marine-Derived Stemphylium sp. Fungus
Source: Mar Drugs. 2020 Aug 21;18(9):436. doi: 10.3390/md18090436 (PMC7551059; doi:10.3390/md18090436)
Supplement: Supplementary file 1 [file marinedrugs-18-00436-s001.pdf]

Supporting Information

Bioactive Bianthraquinones and Meroterpenoids from a Marine Sponge-Derived *Stemphylium* sp. Fungus

Ji-Yeon Hwang,[1] Sung Chul Park,[1] Woong Sub Byun,[1] Dong-Chan Oh,[1] Sang Kook Lee,[1] Ki-Bong Oh,[2,*] and Jongheon Shin[1,*]

[1]*Natural Products Research Institute, College of Pharmacy, Seoul National University, San 56-1, Sillim, Gwanak, Seoul 151-742, Korea*

[2]*Department of Agricultural Biotechnology, College of Agricultural and Life Science, Seoul National University, San 56-1, Sillim, Gwanak, Seoul 151-921, Korea*

List of Supporting Information

Figure S1 The ^{1}H NMR spectrum of Alterporriol Z1 (**1**) (600MHz, CD_3OD) S4
Figure S2 The ^{13}C NMR spectrum of Alterporriol Z1 (**1**) (150MHz, CD_3OD) S4
Figure S3 The HSQC spectrum of Alterporriol Z1 (**1**) (600MHz, CD_3OD) S5
Figure S4 The COSY spectrum of Alterporriol Z1 (**1**) (600MHz, CD_3OD) S5
Figure S5 The HMBC spectrum of Alterporriol Z1 (**1**) (600MHz, CD_3OD) S6
Figure S6 The LR-HSQMBC spectrum of Alterporriol Z1 (**1**) (800MHz, CD_3OD) S6
Figure S7 The NOESY spectrum of Alterporriol Z1 (**1**) (400MHz, CD_3OD) S7
Figure S8 The ^{1}H NMR spectrum of Alterporriol Z1 (**1**) (800MHz, THF-d_8) S7
Figure S9 The NOESY spectrum of Alterporriol Z1 (**1**) (800MHz, THF-d_8) S8
Figure S10 The HRFABMS data of Alterporriol Z1 (**1**) S8
Figure S11 The ^{1}H NMR spectrum of Alterporriol Z2 (**2**) (400MHz, CD_3OD) S9
Figure S12 The ^{13}C NMR spectrum of Alterporriol Z2 (**2**) (100MHz, CD_3OD) S9
Figure S13 The HSQC spectrum of Alterporriol Z2 (**2**) (400MHz, CD_3OD) S10
Figure S14 The COSY spectrum of Alterporriol Z2 (**2**) (400MHz, CD_3OD) S10
Figure S15 The HMBC spectrum of Alterporriol Z2 (**2**) (400MHz, CD_3OD) S11
Figure S16 The NOESY spectrum of Alterporriol Z2 (**2**) (400MHz, CD_3OD) S11
Figure S17 The HRFABMS data of Alterporriol Z2 (**2**) S12
Figure S18 The ^{1}H NMR spectrum of Alterporriol Z3 (**3**) (800MHz, CD_3OD) S13
Figure S19 The ^{13}C NMR spectrum of Alterporriol Z3 (**3**) (200MHz, CD_3OD) S13
Figure S20 The HSQC spectrum of Alterporriol Z3 (**3**) (500MHz, CD_3OD) S14
Figure S21 The COSY spectrum of Alterporriol Z3 (**3**) (500MHz, CD_3OD) S14
Figure S22 The HMBC spectrum of Alterporriol Z3 (**3**) (800MHz, CD_3OD) S15
Figure S23 The NOESY spectrum of Alterporriol Z3 (**3**) (500MHz, CD_3OD) S15
Figure S24 The HRFABMS data of Alterporriol Z3 (**3**) S16
Figure S25 The ^{1}H NMR spectrum of Tricycloalterfurene E (**7**) (800MHz, CD_3OD) S17
Figure S26 The ^{13}C NMR spectrum of Tricycloalterfurene E (**7**) (100MHz, CD_3OD) S17
Figure S27 The HSQC spectrum of Tricycloalterfurene E (**7**) (400MHz, CD_3OD) S18
Figure S28 The COSY spectrum of Tricycloalterfurene E (**7**) (400MHz, CD_3OD) S18
Figure S29 The HMBC spectrum of Tricycloalterfurene E (**7**) (800MHz, CD_3OD) S19
Figure S30 The NOESY spectrum of Tricycloalterfurene E (**7**) (600MHz, CD_3OD) S19
Figure S31 The HRFABMS data of Tricycloalterfurene E (**7**) S20
Figure S32 The ^{1}H NMR spectrum of Tricycloalterfurene F (**8**) (800MHz, CD_3OD) S21
Figure S33 The ^{13}C NMR spectrum of Tricycloalterfurene F (**8**) (100MHz, CD_3OD) S21
Figure S34 The HSQC spectrum of Tricycloalterfurene F (**8**) (800MHz, CD_3OD) S22
Figure S35 The COSY spectrum of Tricycloalterfurene F (**8**) (800MHz, CD_3OD) S22
Figure S36 The HMBC spectrum of Tricycloalterfurene F (**8**) (800MHz, CD_3OD) S23
Figure S37 The NOESY spectrum of Tricycloalterfurene F (**8**) (800MHz, CD_3OD) S23
Figure S38 The HRFABMS data of Tricycloalterfurene F (**8**) S24
Figure S39 The ^{1}H NMR spectrum of Tricycloalterfurene G (**9**) (500MHz, CD_3OD) S25
Figure S40 The ^{13}C NMR spectrum of Tricycloalterfurene G (**9**) (100MHz, CD_3OD) S25
Figure S41 The HSQC spectrum of Tricycloalterfurene G (**9**) (500MHz, CD_3OD) S26
Figure S42 The COSY spectrum of Tricycloalterfurene G (**9**) (400MHz, CD_3OD) S26
Figure S43 The HMBC spectrum of Tricycloalterfurene G (**9**) (400MHz, CD_3OD) S27
Figure S44 The NOESY spectrum of Tricycloalterfurene G (**9**) (400MHz, CD_3OD) S27
Figure S45 The HRFABMS data of Tricycloalterfurene G (**9**) S28
Figure S46 The ^{1}H NMR spectrum of (*S*)-MTPA Ester of **7** (800MHz, CD_3OD) S29
Figure S47 The ^{1}H NMR spectrum of (*R*)-MTPA Ester of **7** (800MHz, CD_3OD) S29

Figure S48 The ^{1}H NMR spectrum of (*S*)-MTPA Ester of **8** (800MHz, CD_3OD) S30
Figure S49 The ^{1}H NMR spectrum of (*R*)-MTPA Ester of **8** (800MHz, CD_3OD) S30
Figure S50 The ^{1}H NMR spectrum of (*S*)-PGME Amide of **7** (800MHz, CD_3OD) S31
Figure S51 The ^{1}H NMR spectrum of (*R*)-PGME Amide of **7** (800MHz, CD_3OD) S31
Figure S52 The ^{1}H NMR spectrum of (*S*)-PGME Amide of **8** (800MHz, CD_3OD) S32
Figure S53 The ^{1}H NMR spectrum of (*R*)-PGME Amide of **8** (800MHz, CD_3OD) S32
Figure S54 The results of DP4 analyses of Alterporriol Z1 (**1**) S33
Figure S55 The viability of RAW 264.7 cells was measured using the MTT assay S34
Table S1 Experimental (Exp.) and calculated (Cal.) chemical shift values of enantiomers A and B on aliphatic ring part of Alterporriol Z1 (**1**) S35
Table S2 The ^{1}H NMR Data of Alterporriol Z1 (**1**) in THF-d_8 S36

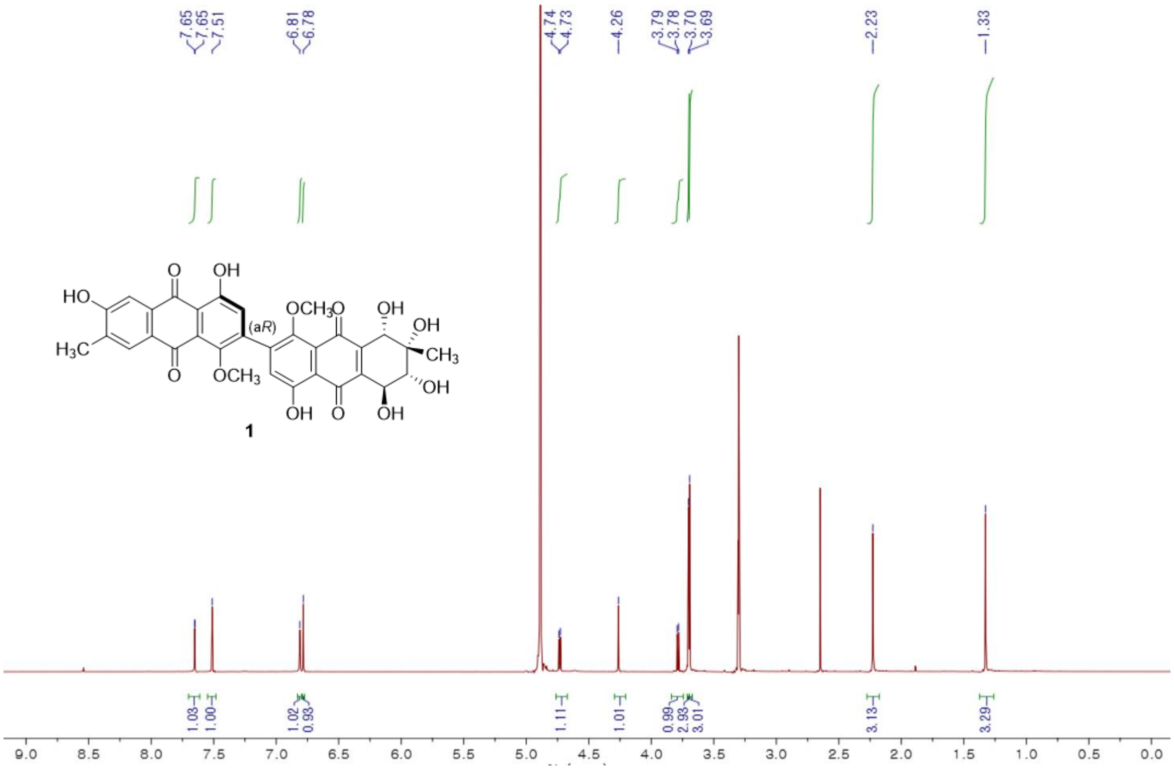

Figure S1. The ^{1}H NMR spectrum of Alterporriol Z1 (**1**) (600MHz, CD_3OD)

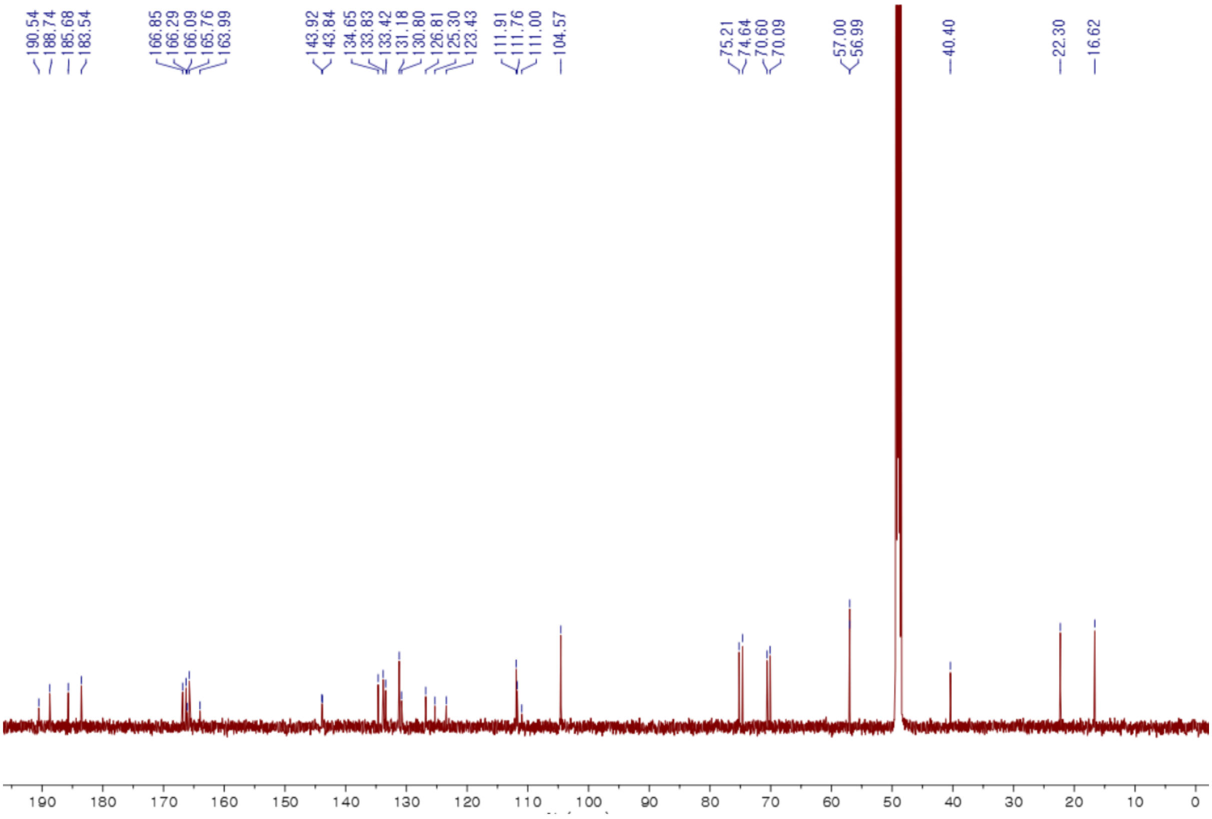

Figure S2. The ^{13}C NMR spectrum of Alterporriol Z1 (**1**) (150MHz, CD_3OD)

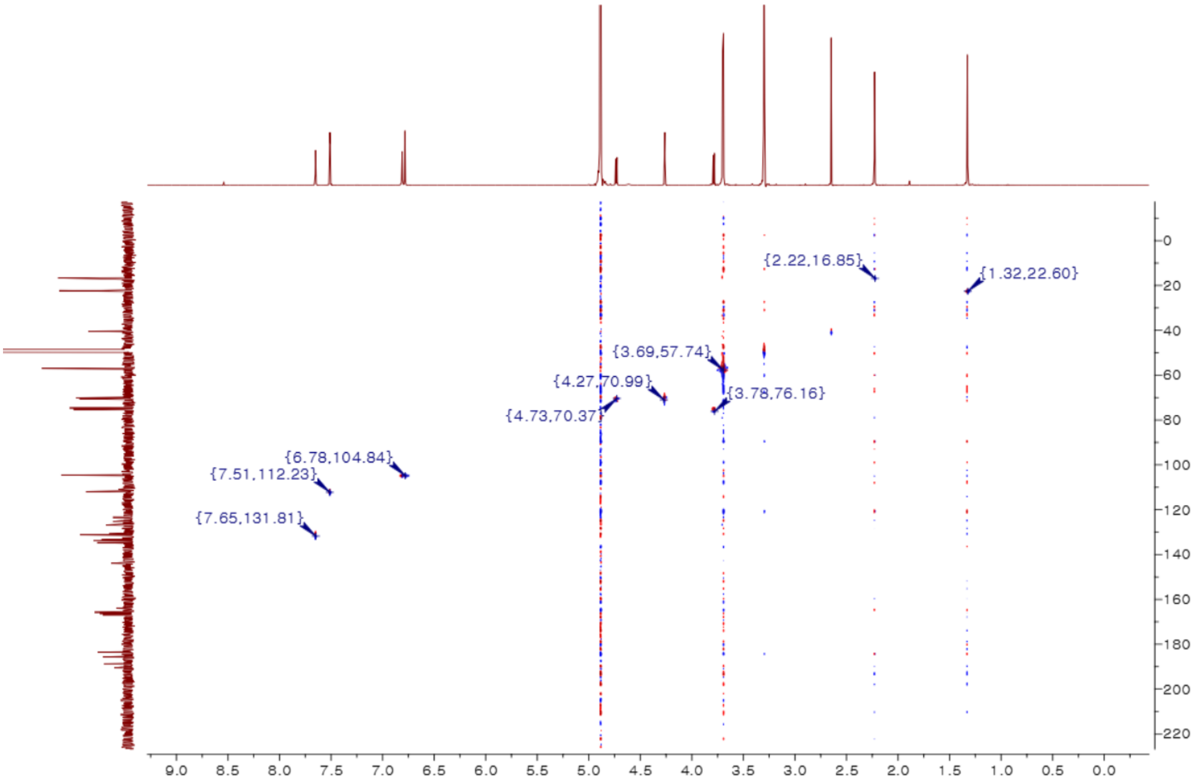

Figure S3. The HSQC spectrum of Alterporriol Z1 (**1**) (600MHz, CD_3OD)

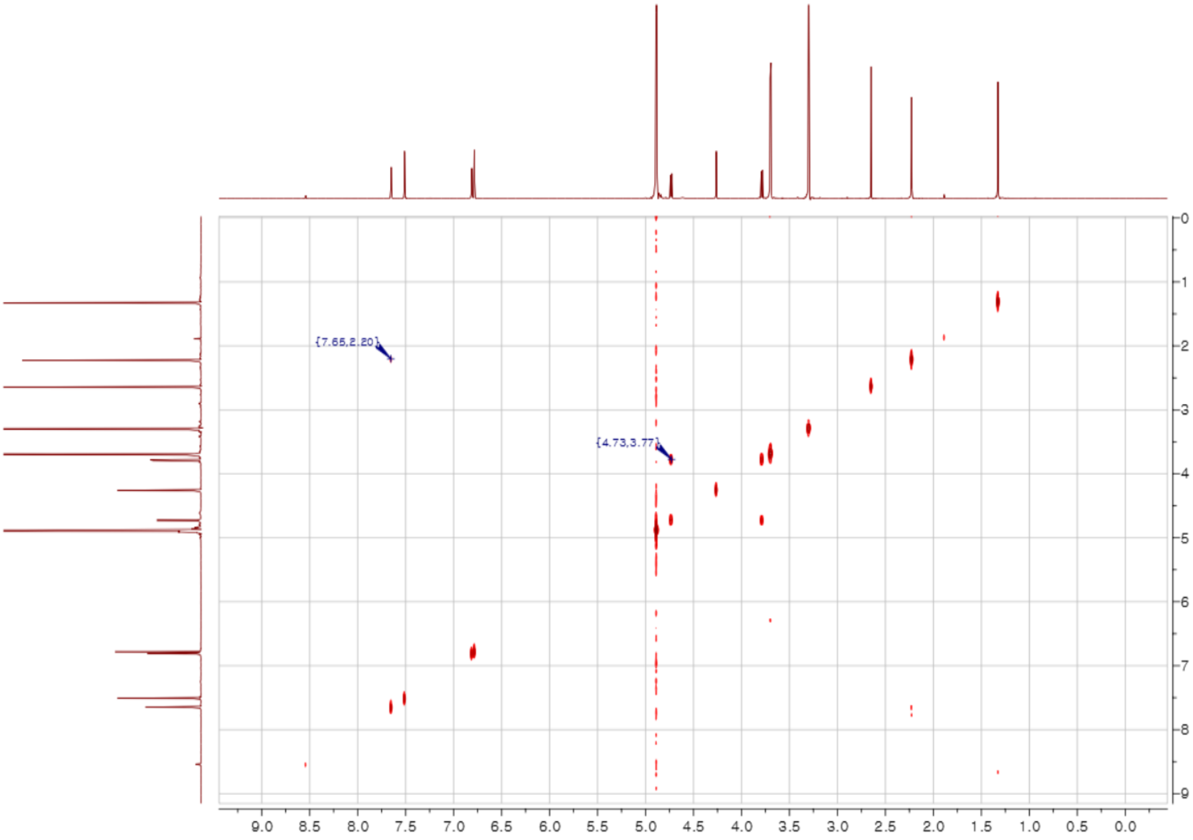

Figure S4. The COSY spectrum of Alterporriol Z1 (**1**) (600MHz, CD_3OD)

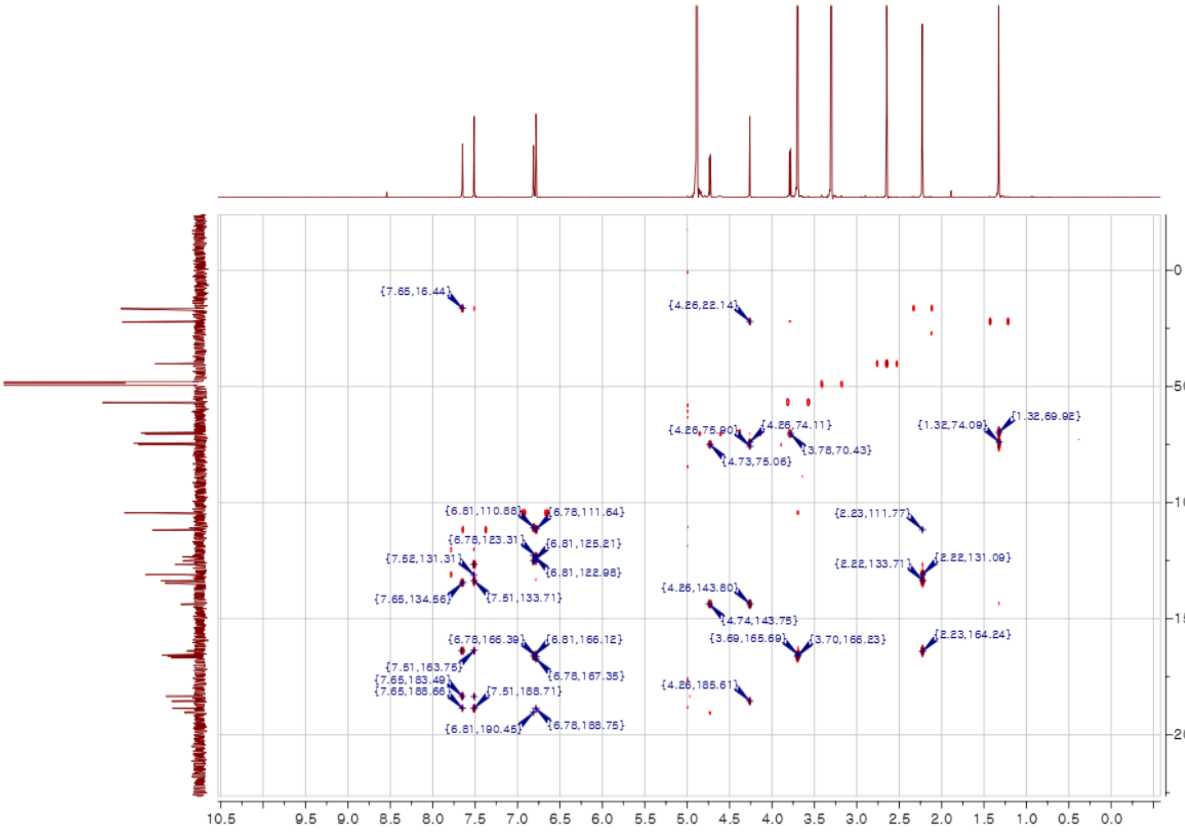

Figure S5. The HMBC spectrum of Alterporriol Z1 (**1**) (600MHz, CD_3OD)

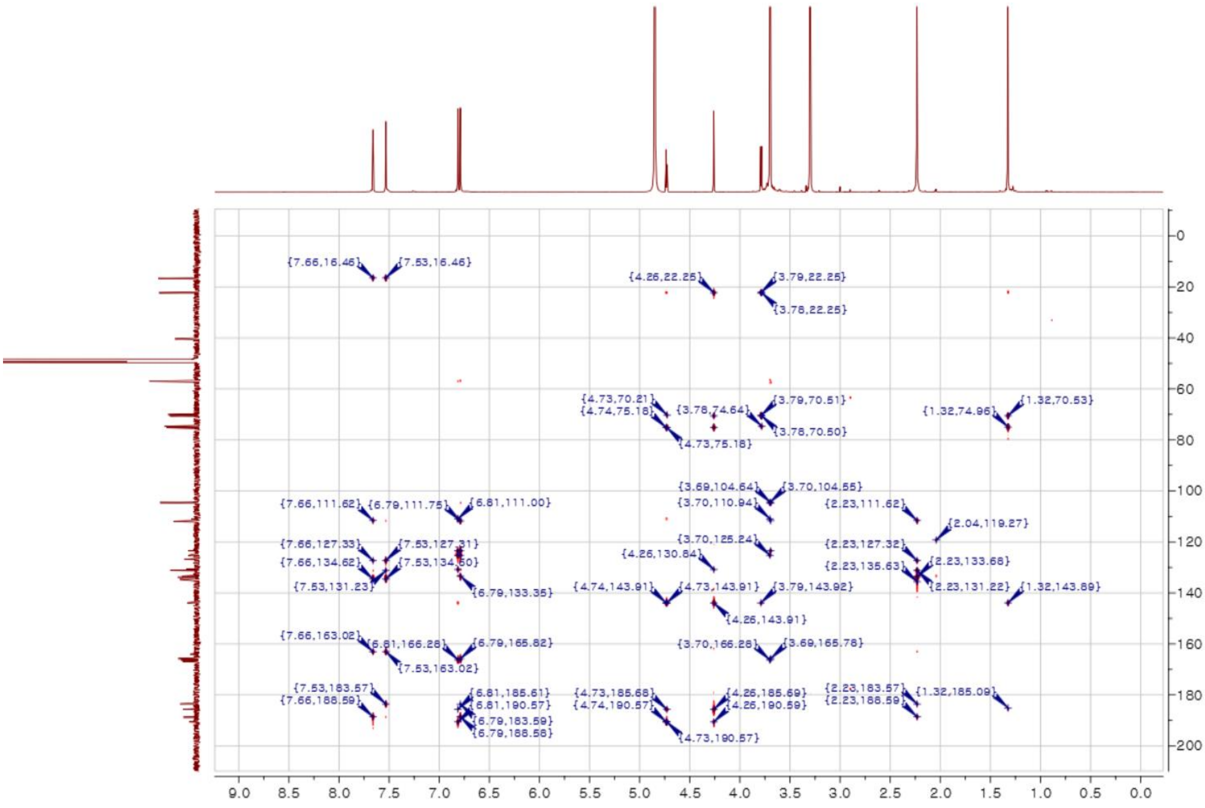

Figure S6. The LR-HSQMBC spectrum of Alterporriol Z1 (**1**) (800MHz, CD_3OD)

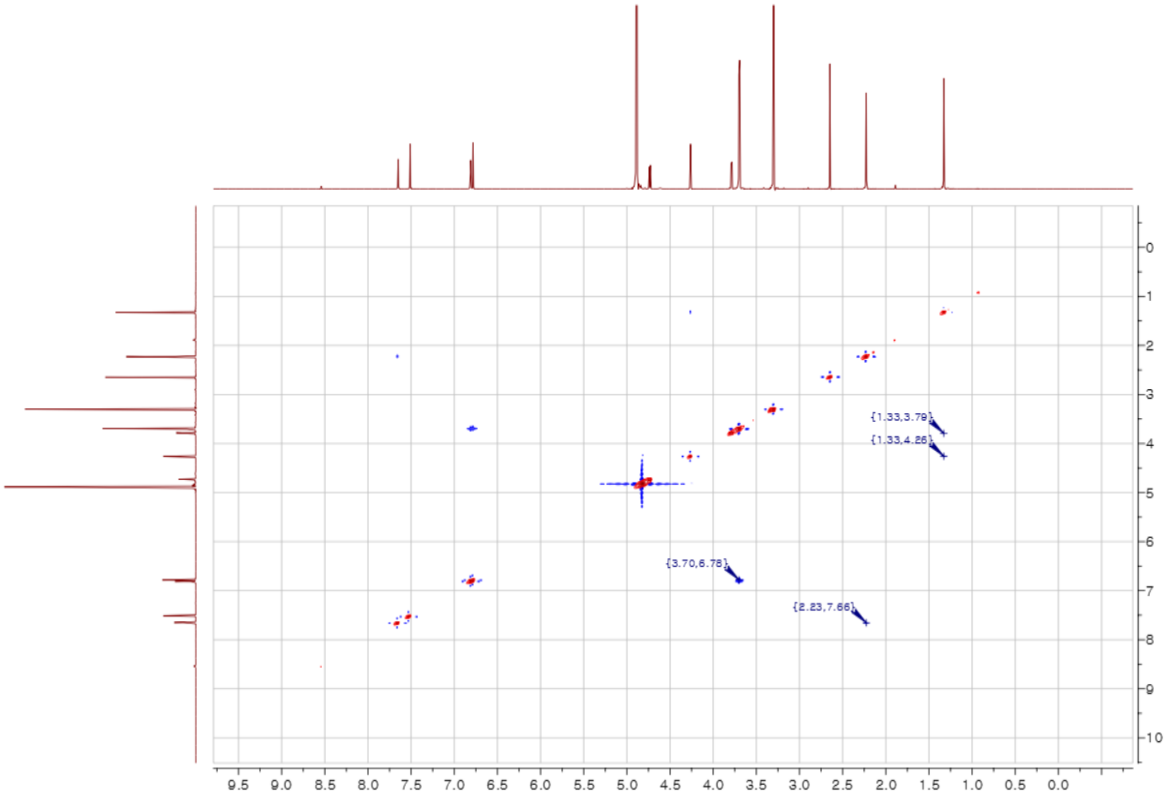

Figure S7. The NOESY spectrum of Alterporriol Z1 (**1**) (400MHz, CD_3OD)

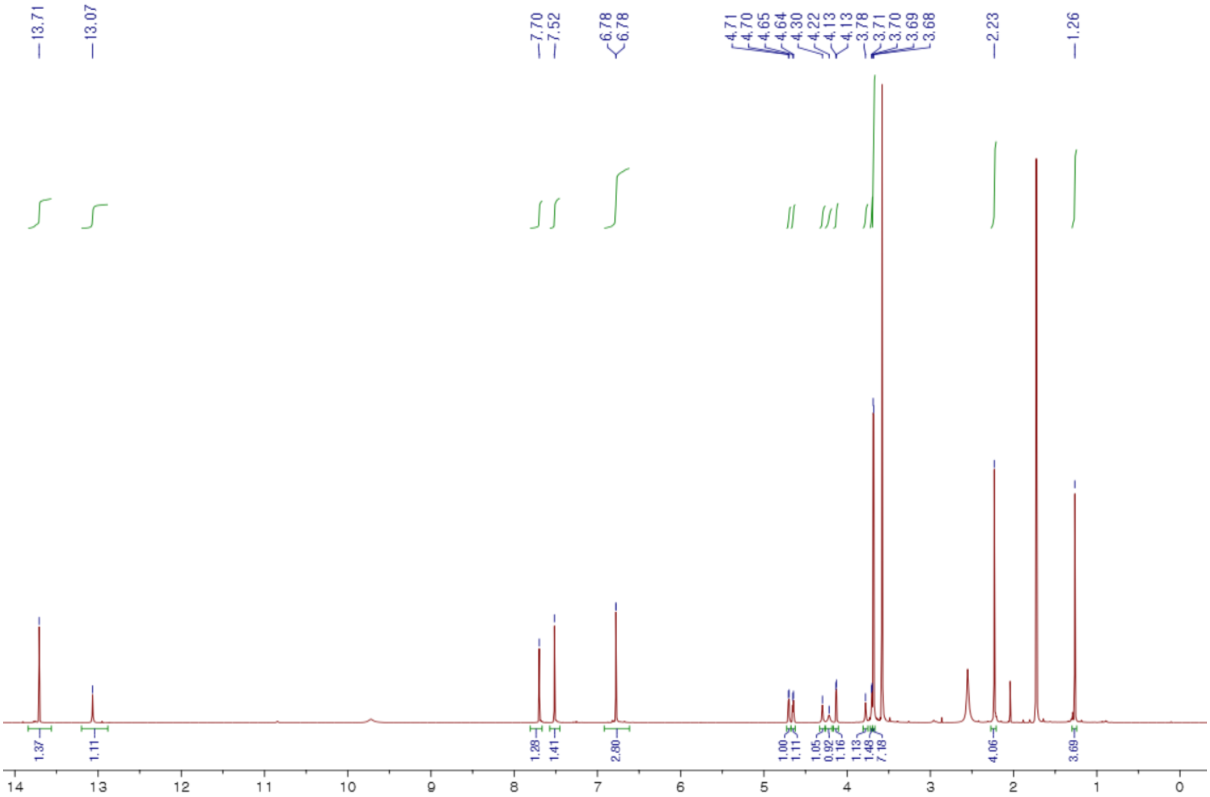

Figure S8. The ^1H NMR spectrum of Alterporriol Z1 (**1**) (800MHz, THF-d_8)

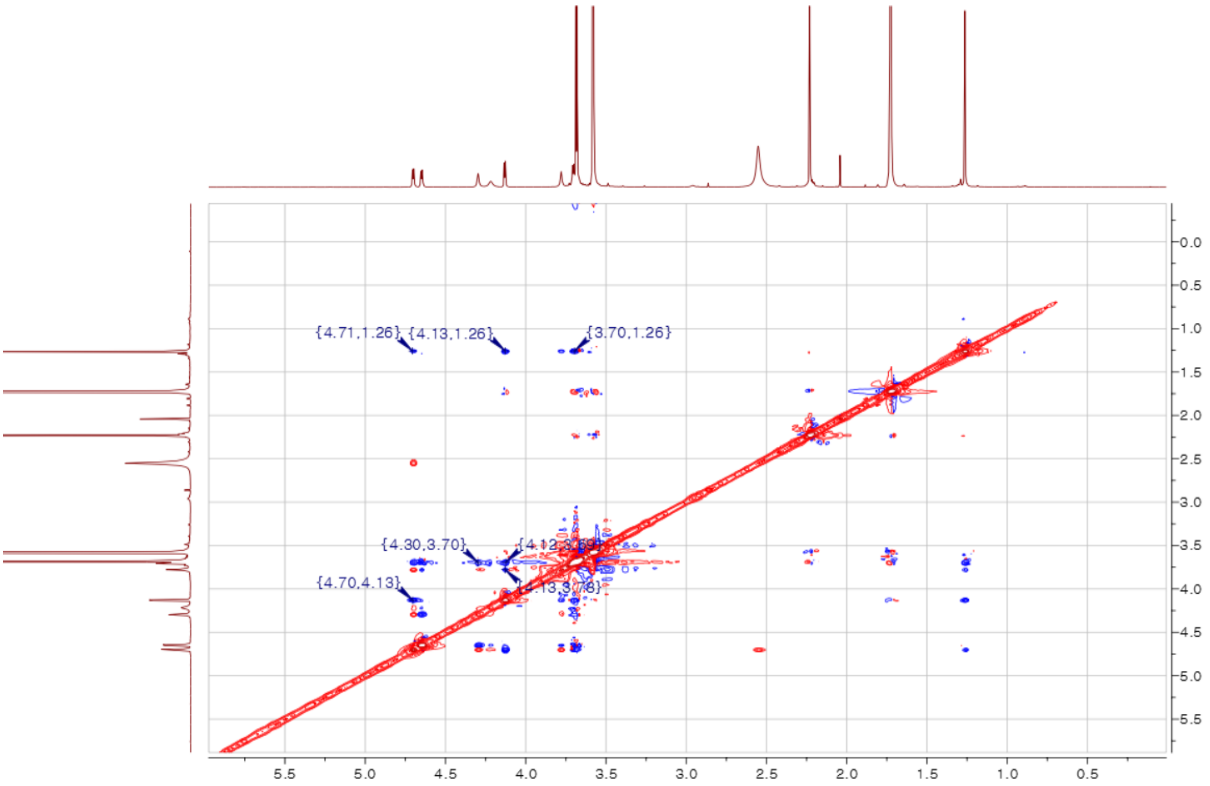

Figure S9. The NOESY spectrum of Alterporriol Z1 (**1**) (800MHz, THF-*d*8)

```
[ Elemental Composition ]                                        Page: 1
Data   : HJH-1-C32H26O13             Date : 24-Oct-2016 15:29
Sample: -
Note : -
Inlet : Direct                       Ion Mode : FAB+
RT : 0.43 min                        Scan#: (9,10)
Elements : C 32/0, H 27/0, O 13/0, Na 1/0
Mass Tolerance        : 1mmu
Unsaturation (U.S.) : -0.5 - 100.0

Observed m/z  Int%   Err[ppm / mmu]    U.S.  Composition
  581.3657    76.2

  618.1377    36.4     +0.6 /  +0.4    20.0  C 32 H 26 O 13

  619.1449   100.0     -0.4 /  -0.3    19.5  C 32 H 27 O 13

  620.1522    52.6

  621.1547    15.2

  625.3936    69.7

  641.1275    62.5     +0.6 /  +0.4    19.5  C 32 H 26 O 13 Na

  642.1318    29.7

  670.4144    15.6
```

```
[ Theoretical Ion Distribution ]                                 Page: 1
Molecular Formula : C32 H27 O13
                       (m/z   619.1452, MW   619.5586, U.S. 19.5)
Base Peak :    619.1452, Averaged MW :   619.5554(a),   619.5562(w)

    m/z        INT.
  619.1452 100.0000 *****************************************************************
  620.1485  36.0867 ***********************
  621.1512   8.9194 *****
  622.1539   1.6527 *
  623.1564   0.2540
  624.1589   0.0335
  625.1614   0.0039
  626.1639   0.0004
```

Figure S10. The HRFABMS data of Alterporriol Z1 (**1**)

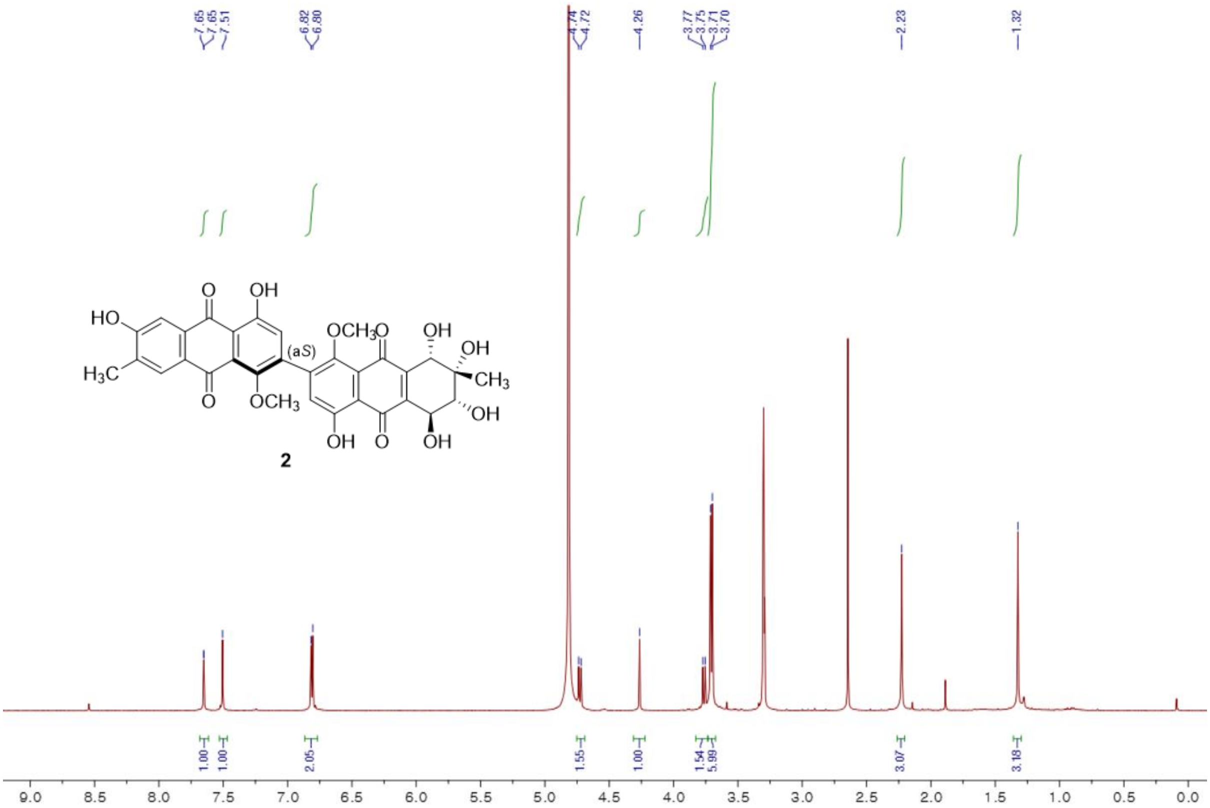

Figure S11. The ^{1}H NMR spectrum of Alterporriol Z2 (**2**) (400MHz, CD_3OD)

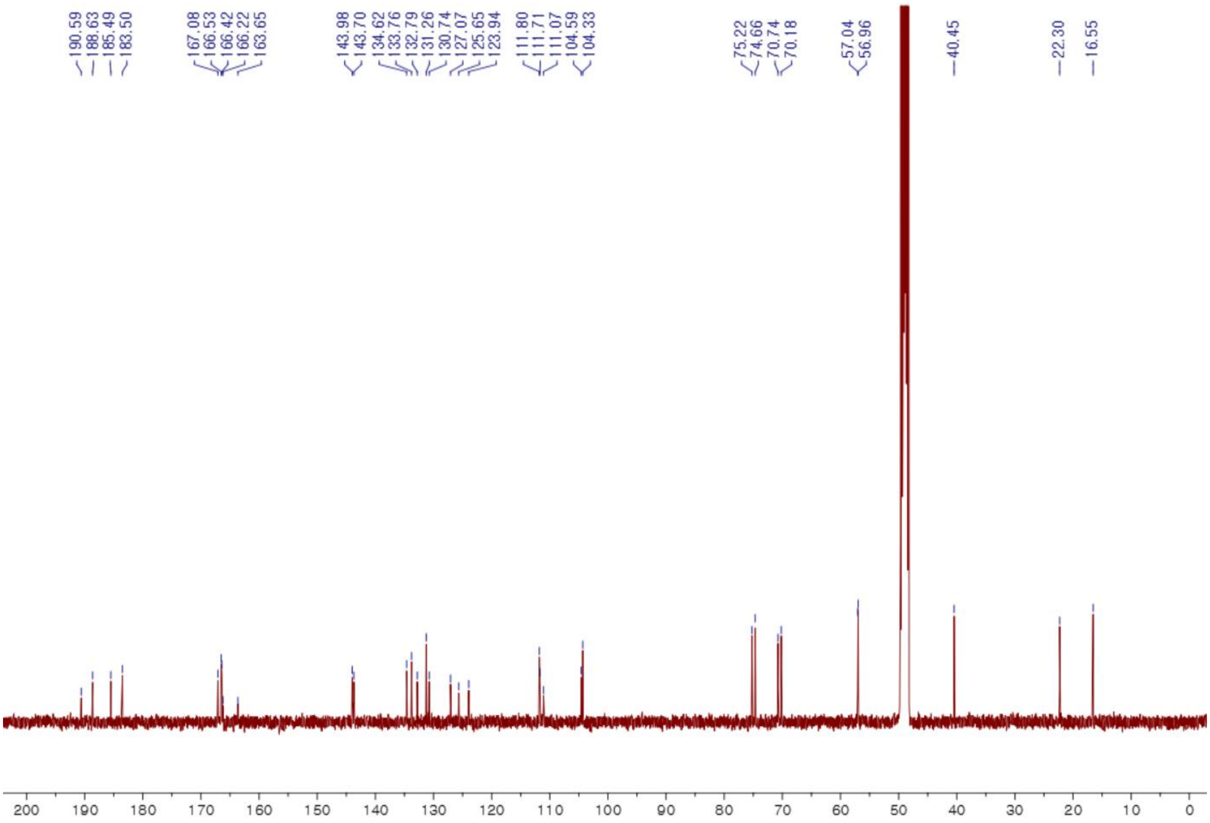

Figure S12. The ^{13}C NMR spectrum of Alterporriol Z2 (**2**) (100MHz, CD_3OD)

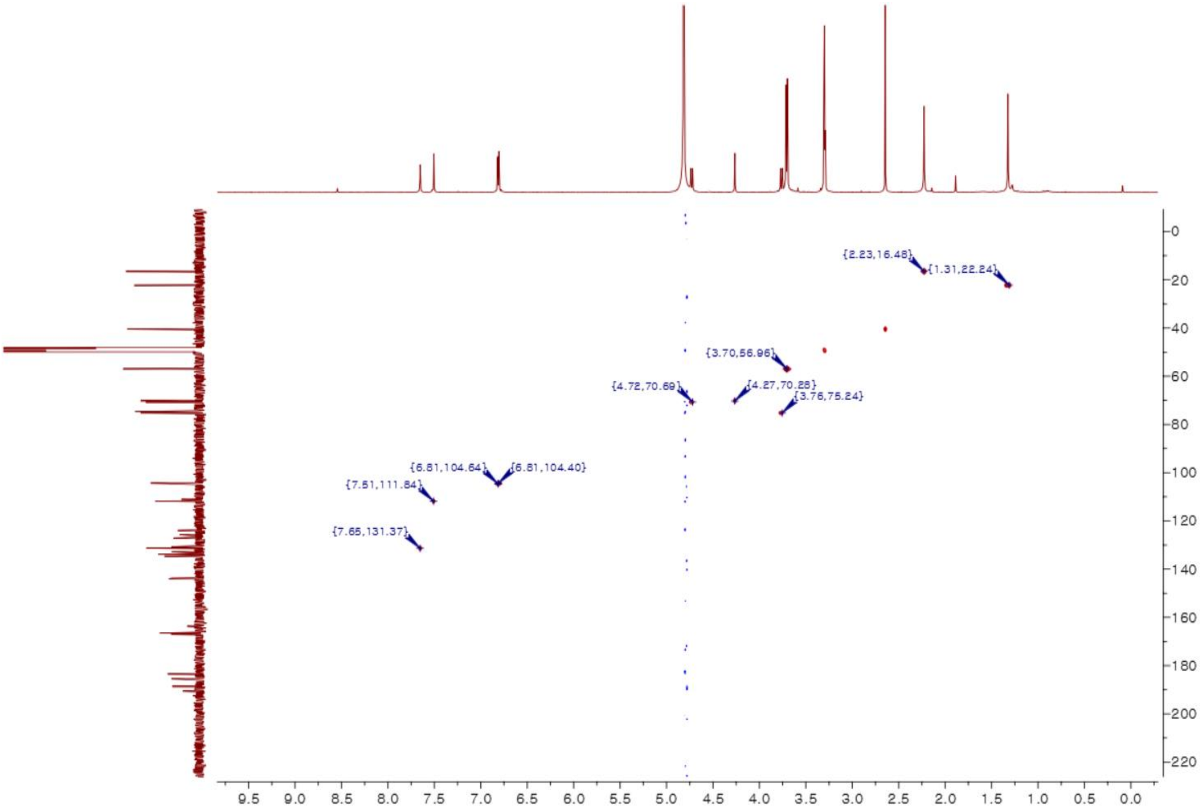

Figure S13. The HSQC spectrum of Alterporriol Z2 (**2**) (400MHz, CD_3OD)

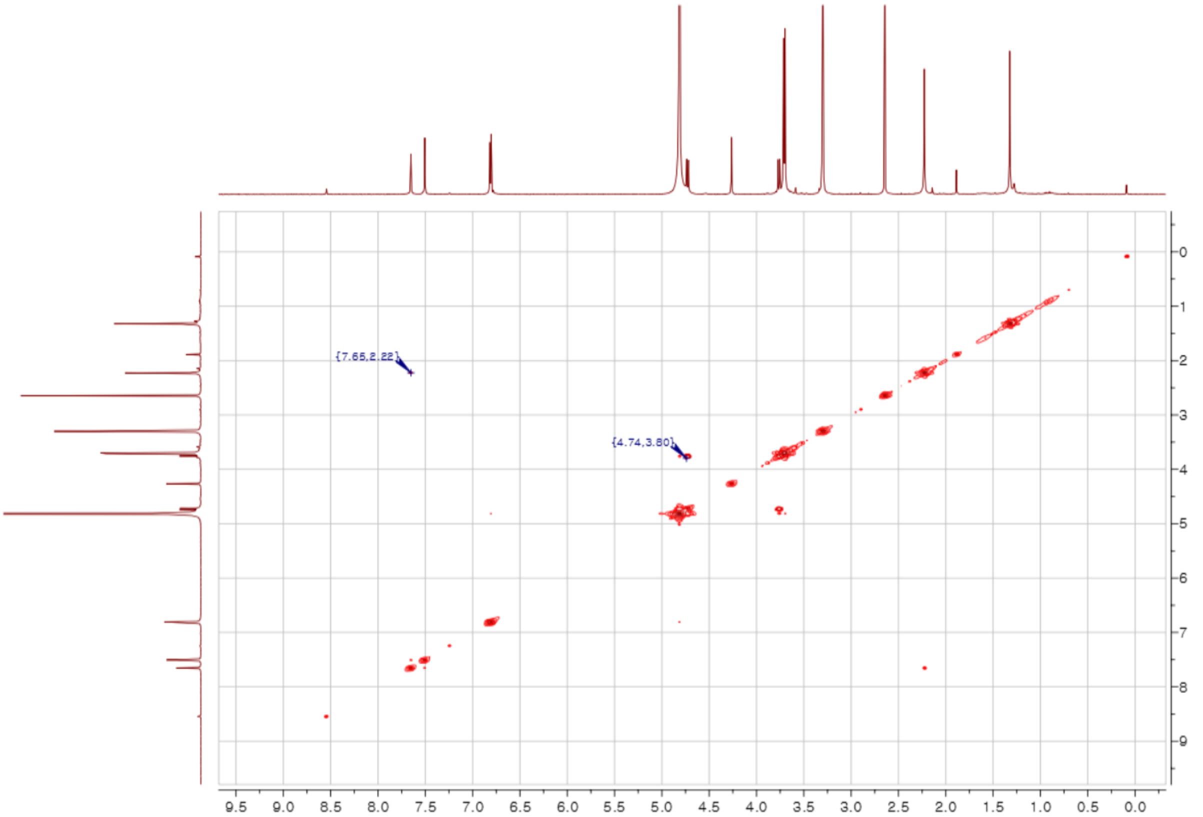

Figure S14. The COSY spectrum of Alterporriol Z2 (**2**) (400MHz, CD_3OD)

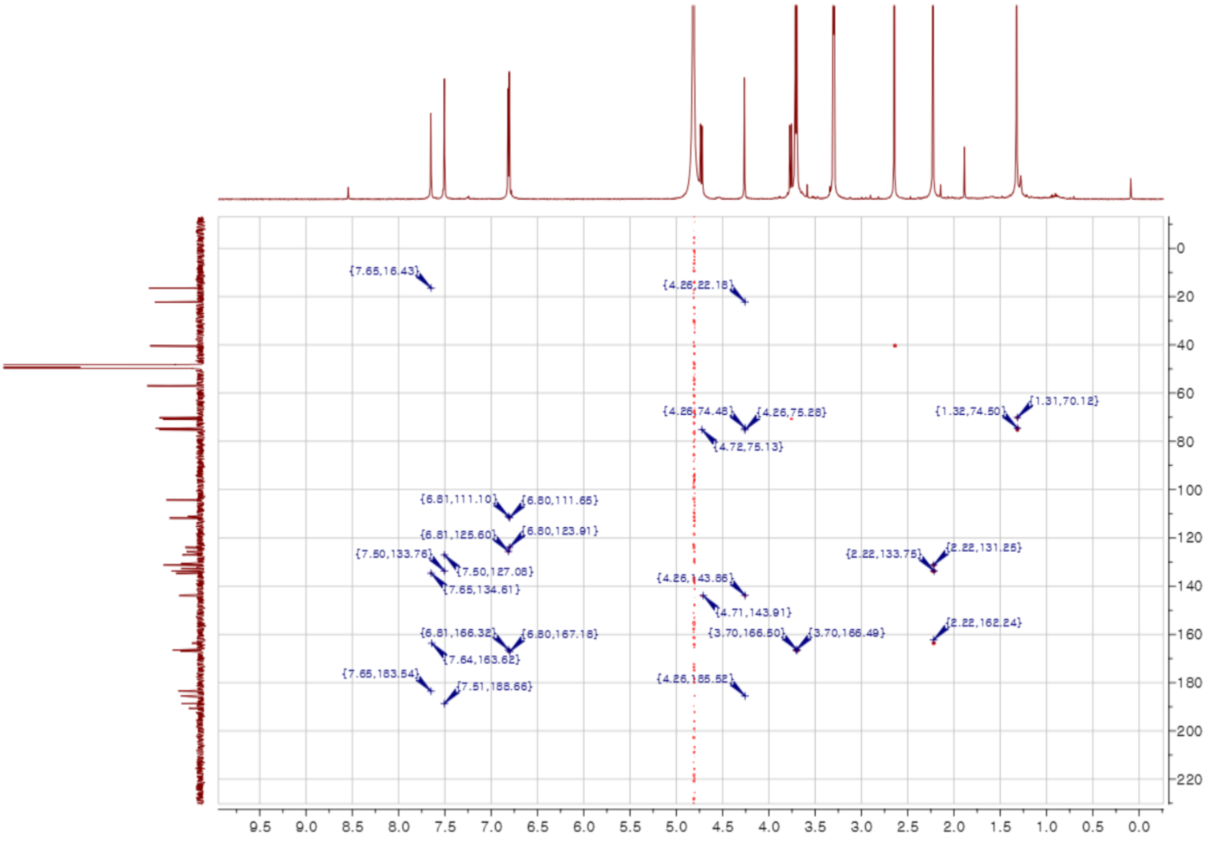

Figure S15. The HMBC spectrum of Alterporriol Z2 (**2**) (400MHz, CD_3OD)

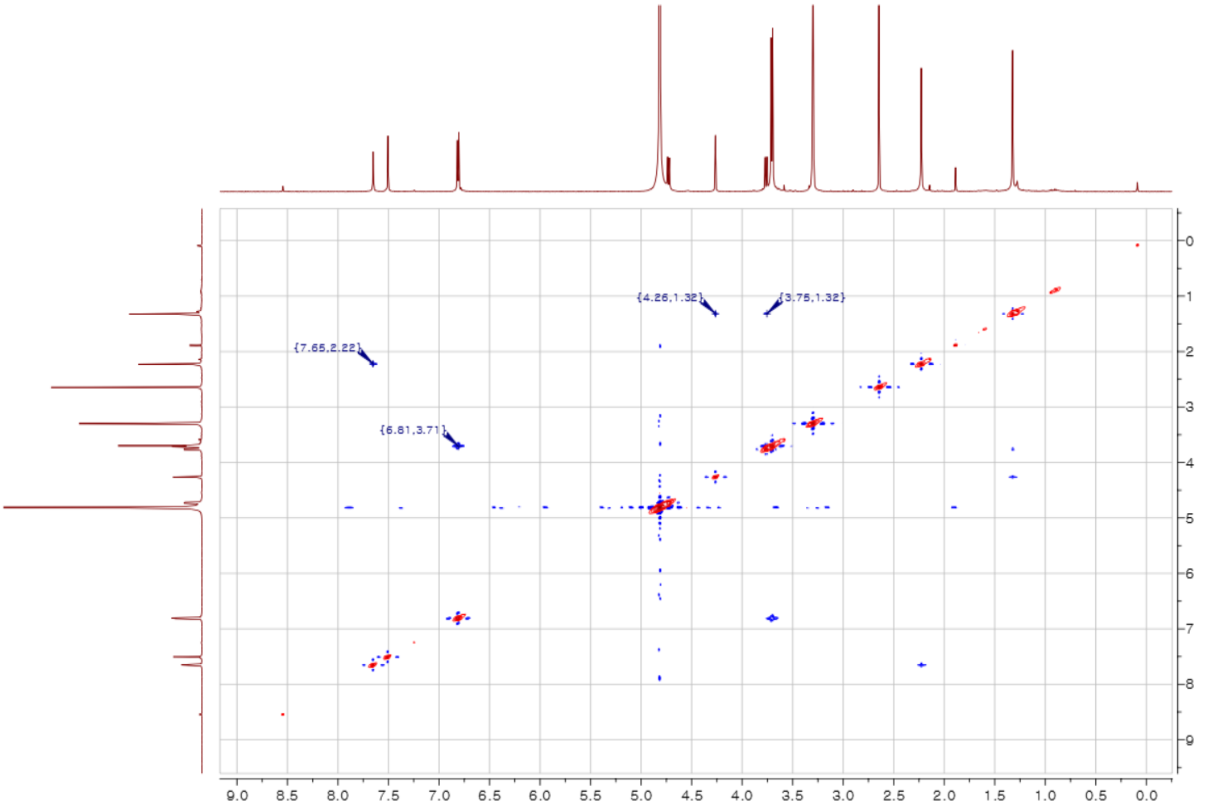

Figure S16. The NOESY spectrum of Alterporriol Z2 (**2**) (400MHz, CD_3OD)

[Elemental Composition]

Page: 1

Data : HJH-2-C32H26O13

Date : 24-Oct-2016 13:52

Sample: -

Note : -

Inlet : Direct

Ion Mode : FAB+

RT : 0.18 min

Scan#: (4,5)

Elements : C 32/0, H 27/0, O 13/0, Na 1/0

Mass Tolerance : 1000ppm, 1mmu if m/z < 1, 2mmu if m/z > 2

Unsaturation (U.S.) : -0.5 - 100.0

Observed m/z	Int%	Err[ppm / mmu]	U.S.	Composition
581.3678	100.0			
618.1376	11.4	+0.4 / +0.2	20.0	C 32 H 26 O 13
619.1454	30.8	+0.4 / +0.2	19.5	C 32 H 27 O 13
620.1486	16.6			
625.3922	27.7			
641.1267	20.8	-0.6 / -0.4	19.5	C 32 H 26 O 13 Na

[Theoretical Ion Distribution]

Page: 1

Molecular Formula : C32 H27 O13

(m/z 619.1452, MW 619.5586, U.S. 19.5)

Base Peak : 619.1452, Averaged MW : 619.5554(a), 619.5562(w)

m/z	INT.	
619.1452	100.0000	**
620.1485	36.0867	**********************
621.1512	8.9194	*****
622.1539	1.6527	*
623.1564	0.2540	
624.1589	0.0335	
625.1614	0.0039	
626.1639	0.0004	

Figure S17. The HRFABMS data of Alterporriol Z2 (**2**)

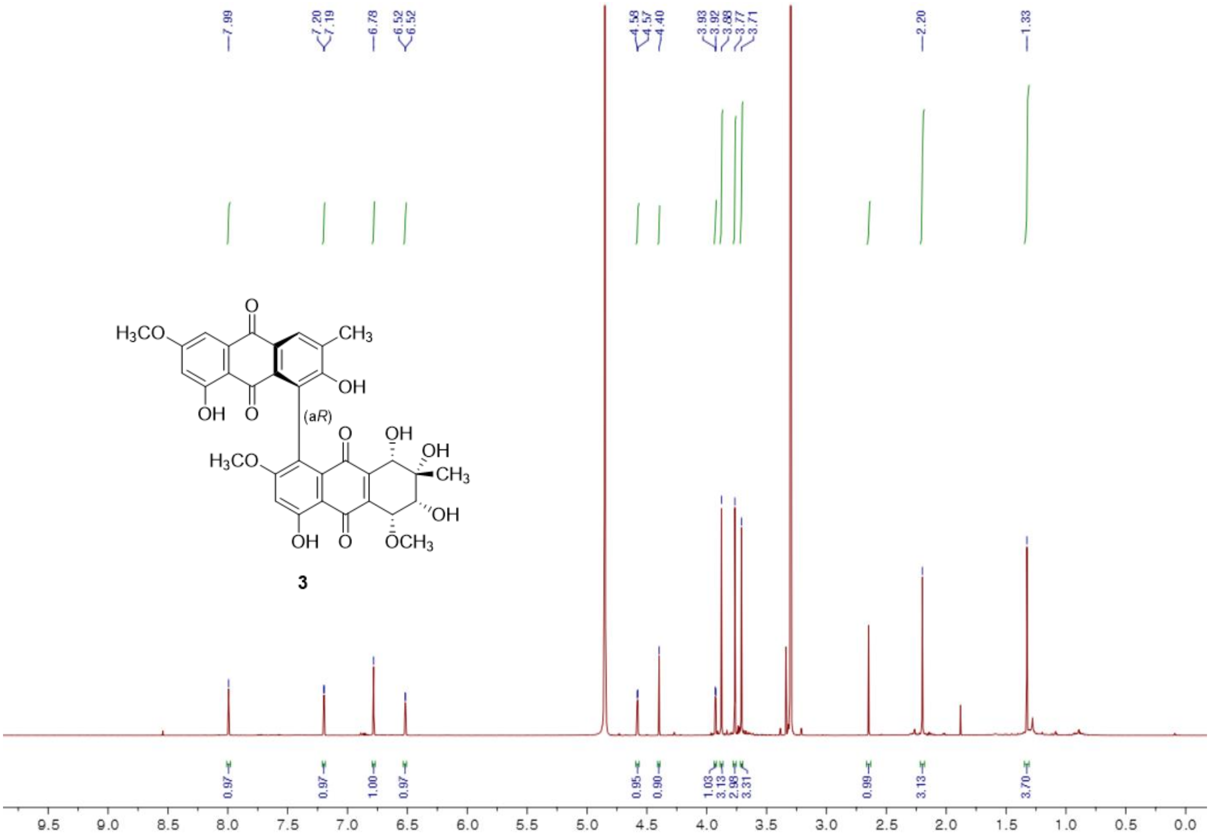

Figure S18. The ^{1}H NMR spectrum of Alterporriol Z3 (**3**) (800MHz, CD_3OD)

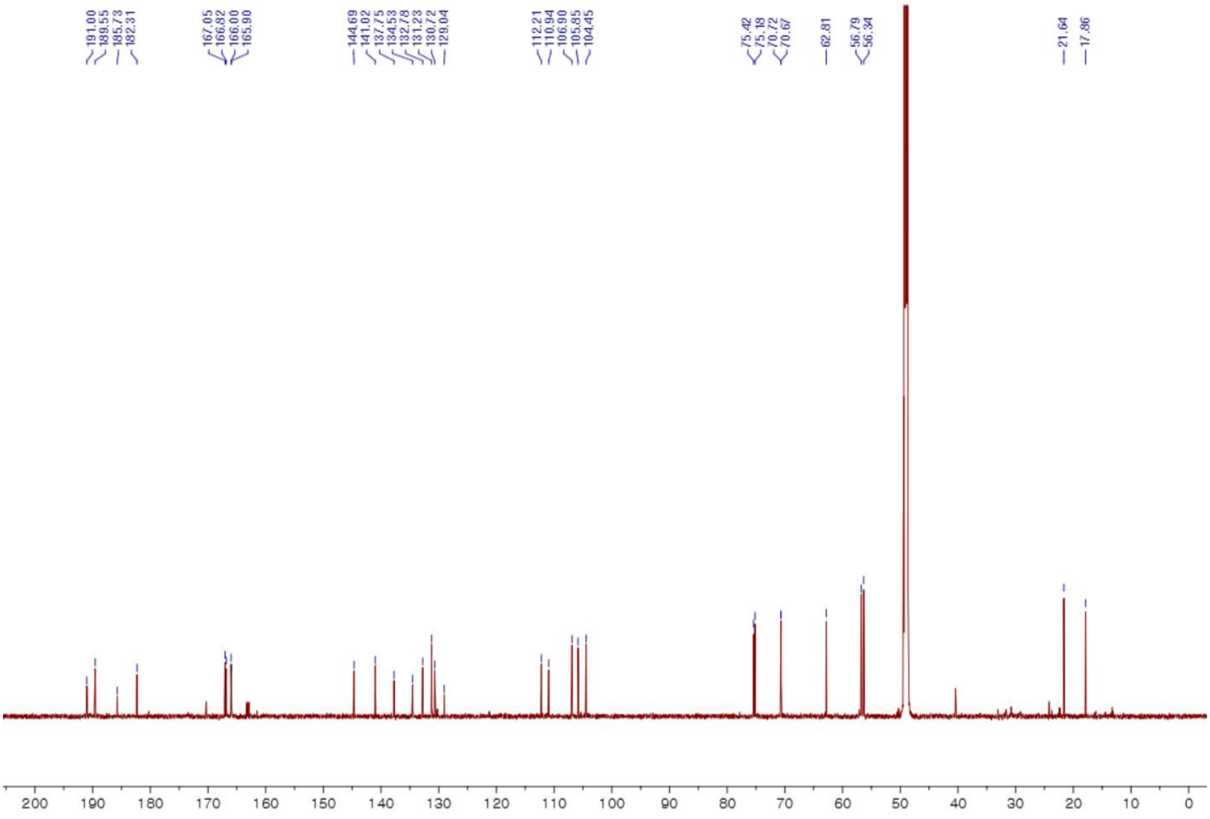

Figure S19. The ^{13}C NMR spectrum of Alterporriol Z3 (**3**) (200MHz, CD_3OD)

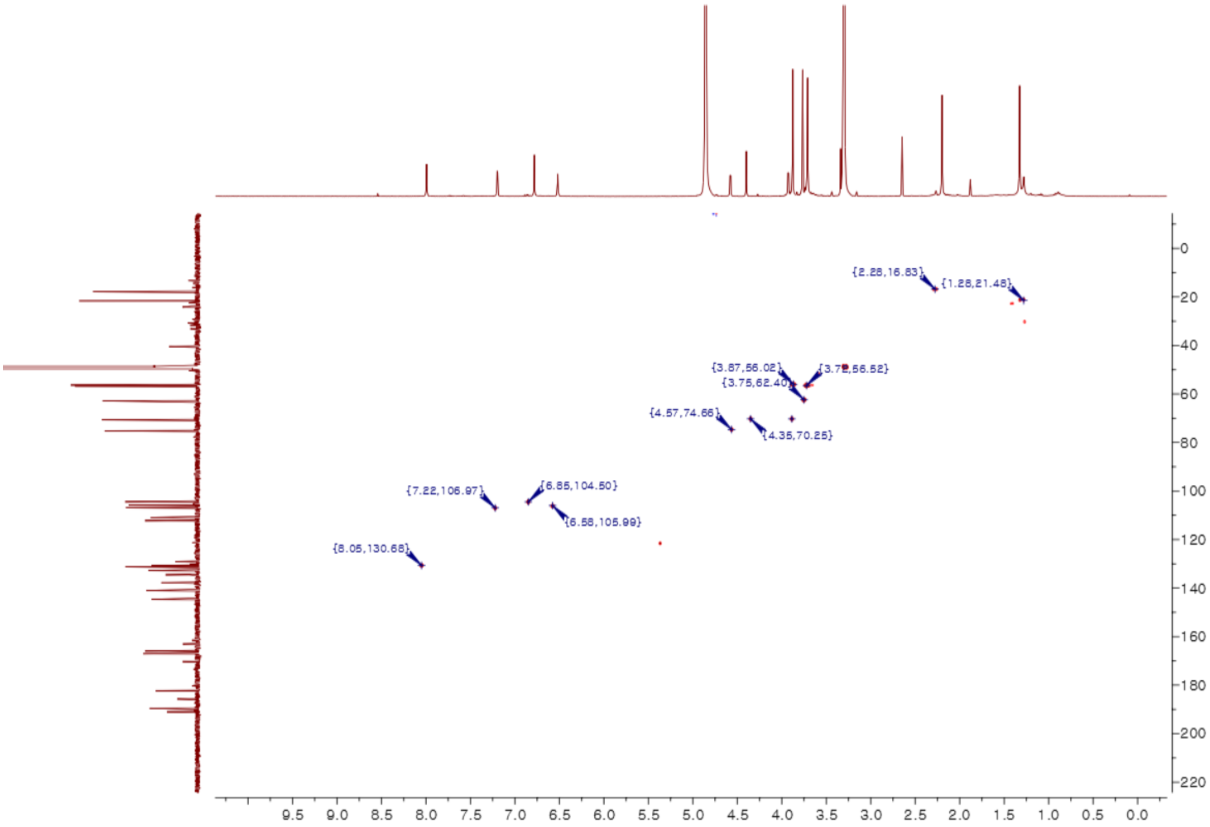

Figure S20. The HSQC spectrum of Alterporriol Z3 (**3**) (500MHz, CD_3OD)

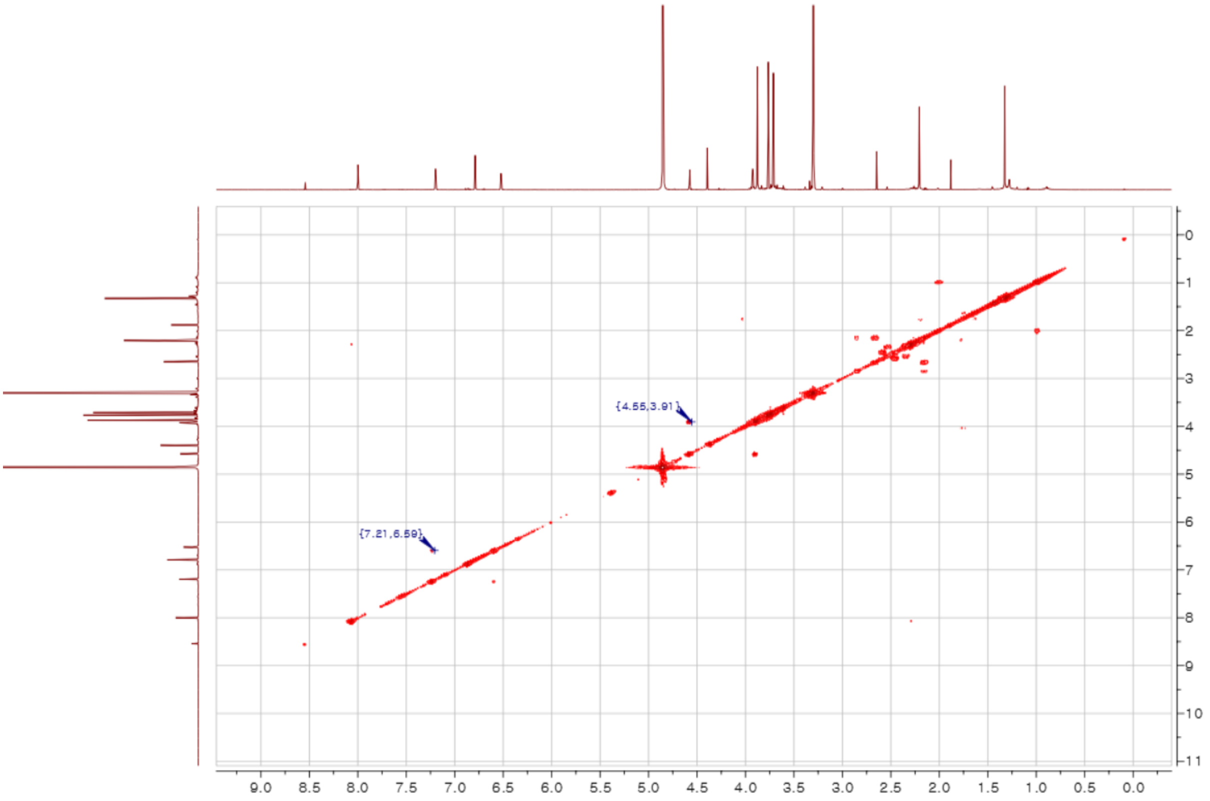

Figure S21. The COSY spectrum of Alterporriol Z3 (**3**) (500MHz, CD_3OD)

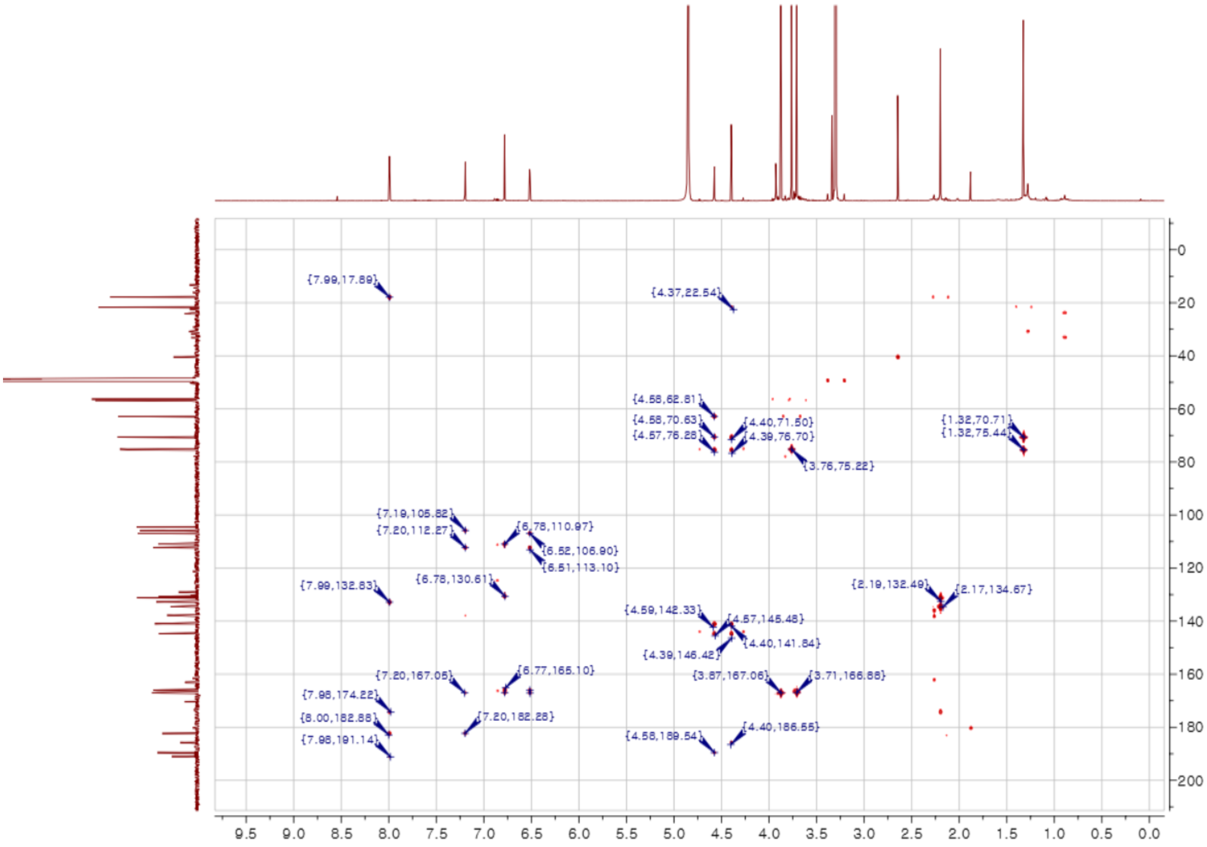

Figure S22. The HMBC spectrum of Alterporriol Z3 (**3**) (800MHz, CD_3OD)

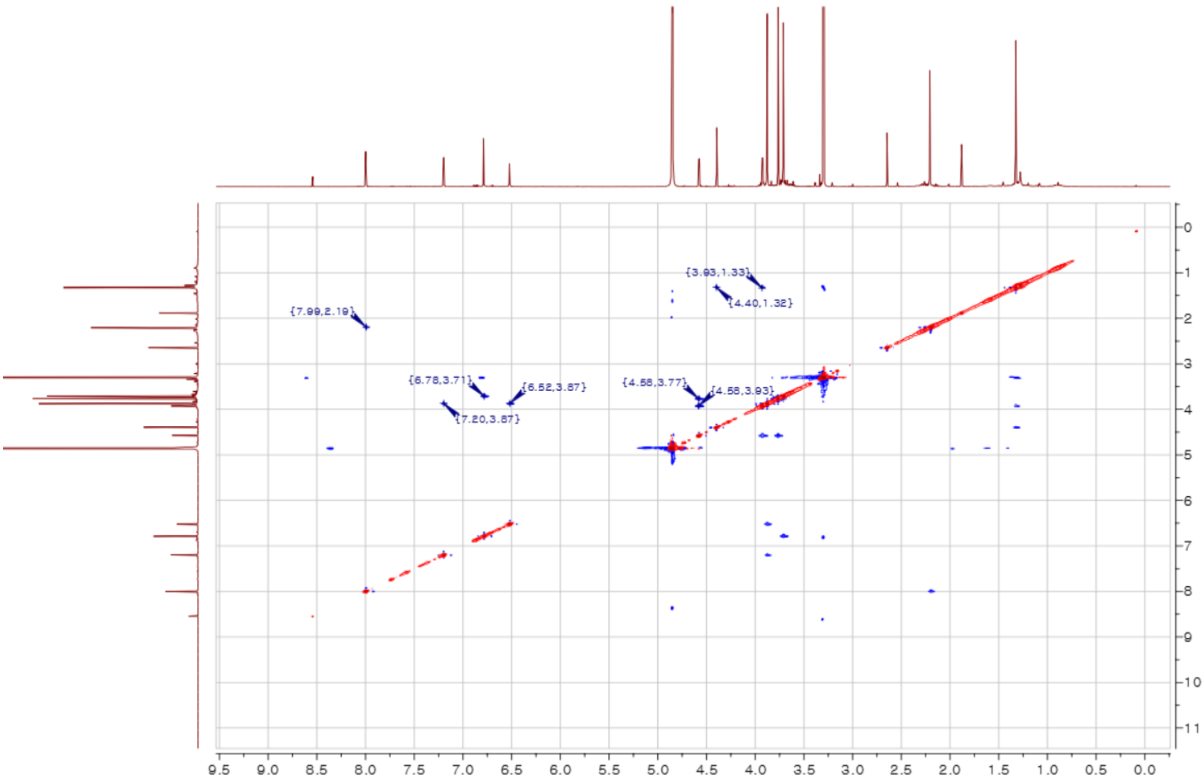

Figure S23. The NOESY spectrum of Alterporriol Z3 (**3**) (500MHz, CD_3OD)

```
[ Elemental Composition ]                                          Page: 1
Data  : HJH-3-C33H28O13              Date : 24-Oct-2016 14:10
Sample: -
Note : -
Inlet : Direct                       Ion Mode : FAB+
RT : 0.68 min                        Scan#: (14,15)
Elements : C 33/0, H 28/0, O 13/0, Na 1/0
Mass Tolerance       : 1000ppm, 1mmu if m/z < 1, 3mmu if m/z > 3
Unsaturation (U.S.)  : -0.5 - 100.0

Observed m/z Int%   Err[ppm / mmu]    U.S.  Composition
  654.1349    29.7     -0.1 /  +0.0   20.0  C 33 H 27 O 13 Na

  655.1430   100.0     +0.4 /  +0.2   19.5  C 33 H 28 O 13 Na

  656.1479    46.1

  657.1496    13.4

  677.1232    17.0

  678.1359    11.8
```

```
[ Theoretical Ion Distribution ]                                   Page: 1
Molecular Formula : C33 H28 O13 Na
                       (m/z   655.1428, MW   655.5673, U.S. 19.5)
Base Peak :   655.1428, Averaged MW :   655.5641(a),   655.5648(w)

    m/z       INT.
  655.1428 100.0000 **************************************************************
  656.1461  37.1989 ***********************
  657.1488   9.3208 *****
  658.1515   1.7519 *
  659.1541   0.2724
  660.1566   0.0363
  661.1591   0.0043
  662.1616   0.0005
```

Figure S24. The HRFABMS data of Alterporriol Z3 (**3**)

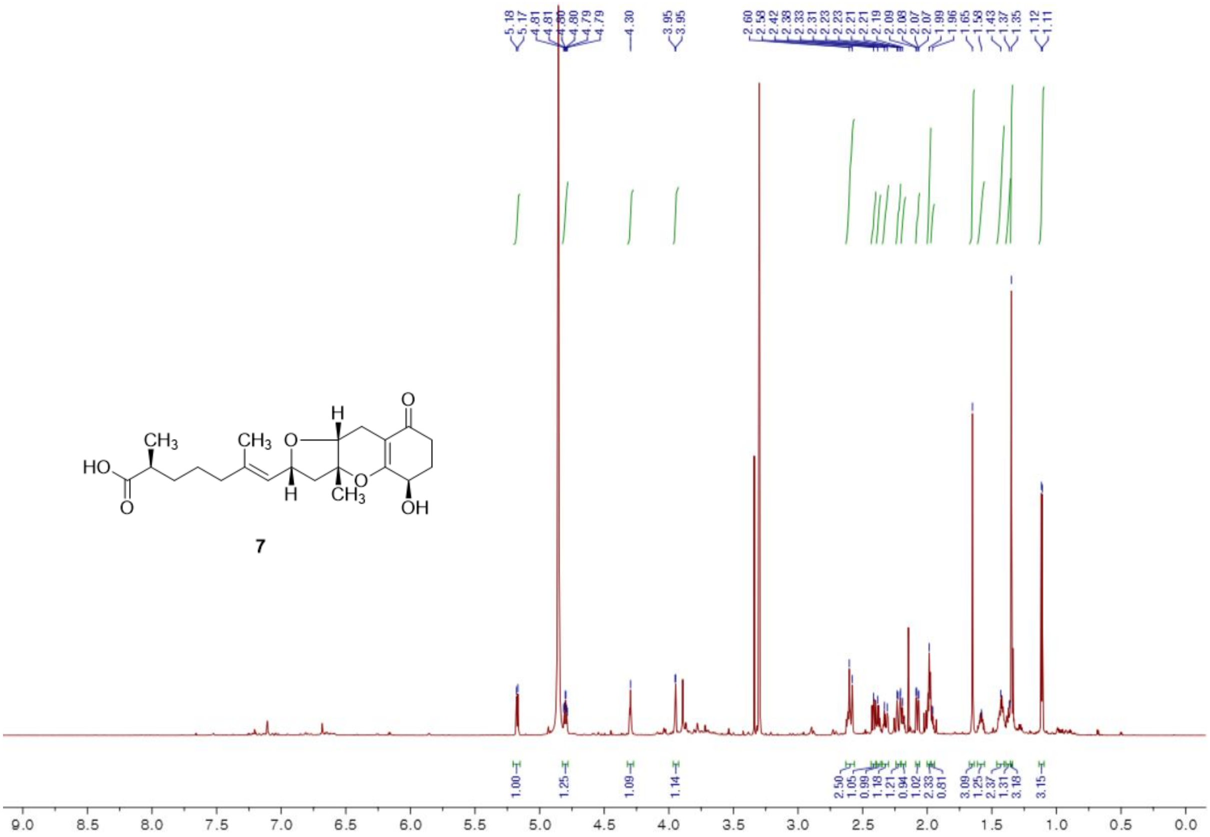

Figure S25. The ^{1}H NMR spectrum of Tricycloalterfurene E (**7**) (800MHz, CD_3OD)

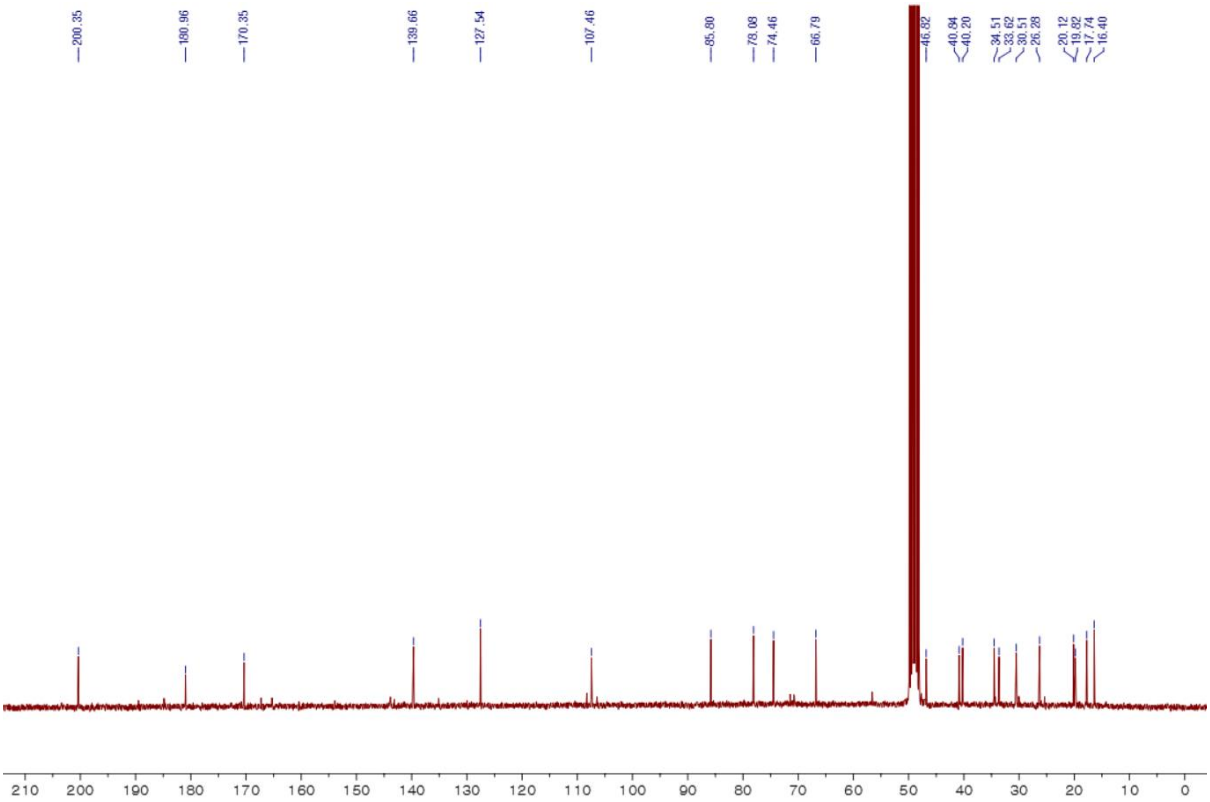

Figure S26. The ^{13}C NMR spectrum of Tricycloalterfurene E (**7**) (100MHz, CD_3OD)

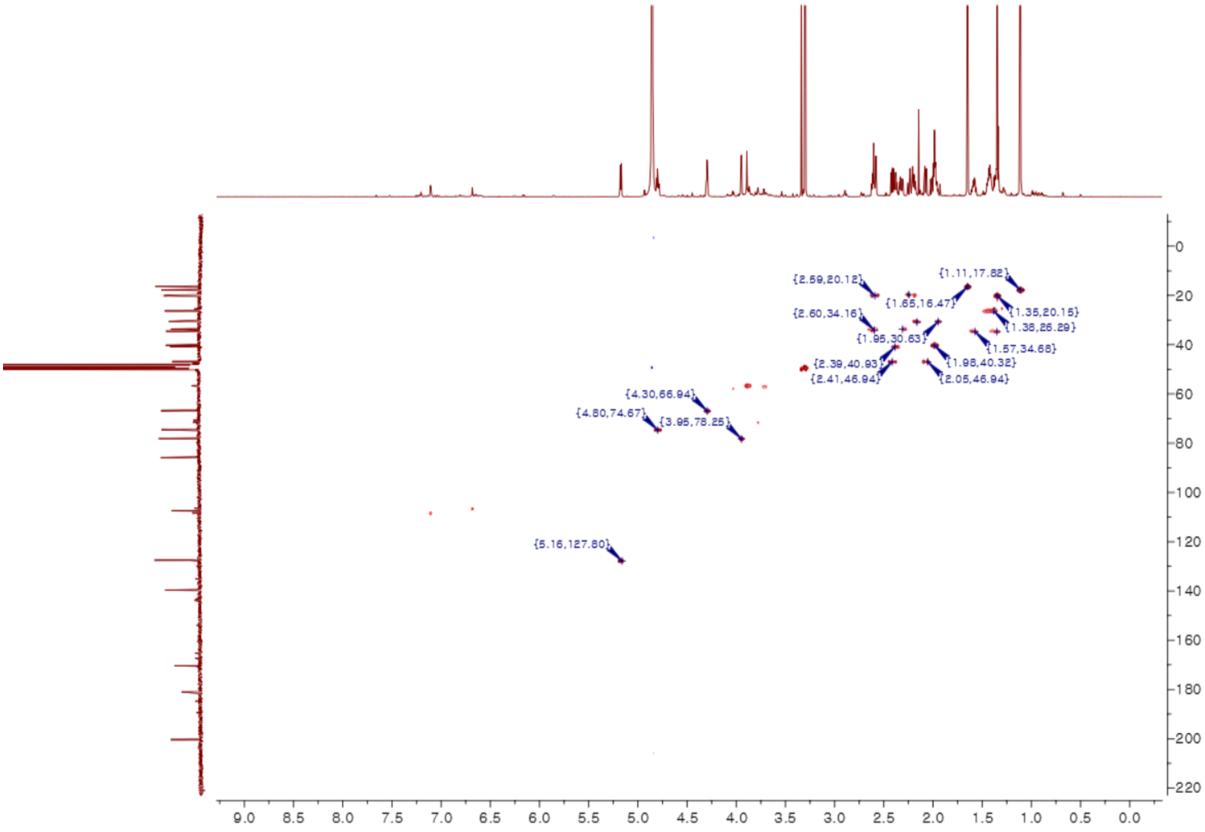

Figure S27. The HSQC spectrum of Tricycloalterfurene E (**7**) (400MHz, CD_3OD)

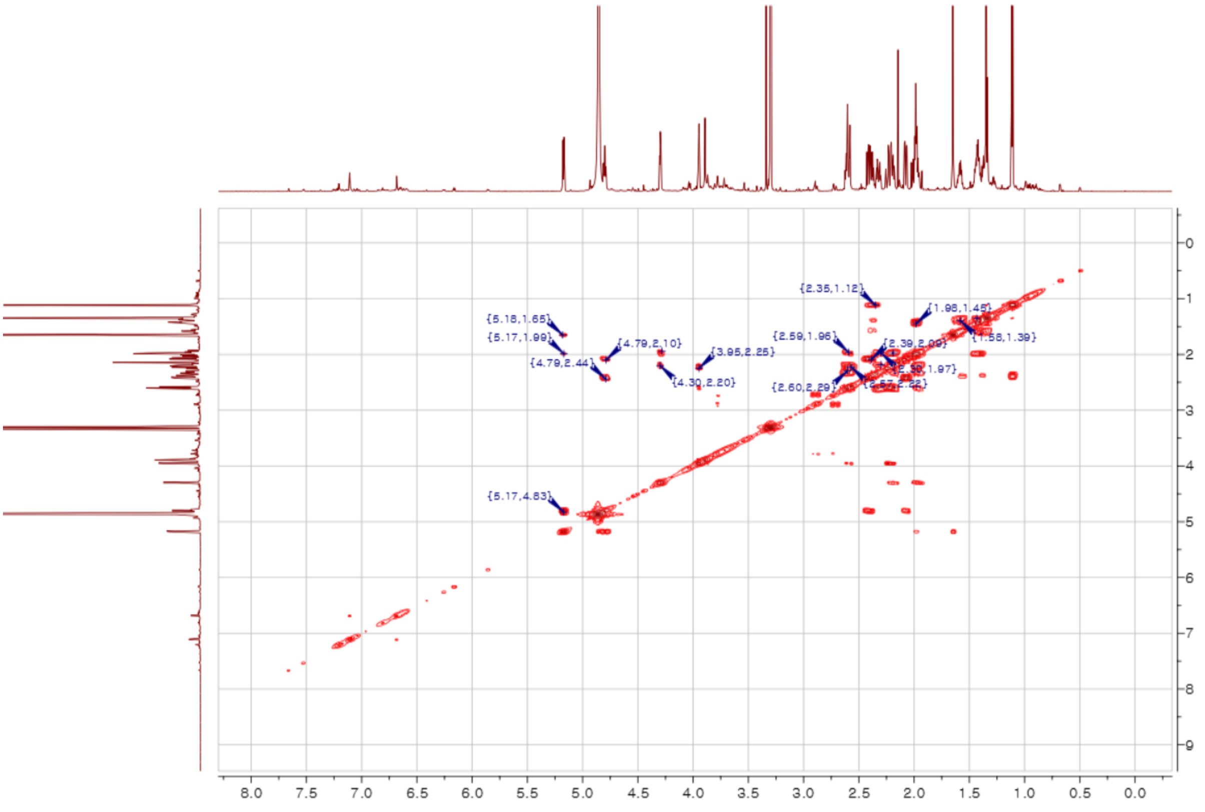

Figure S28. The COSY spectrum of Tricycloalterfurene E (**7**) (400MHz, CD_3OD)

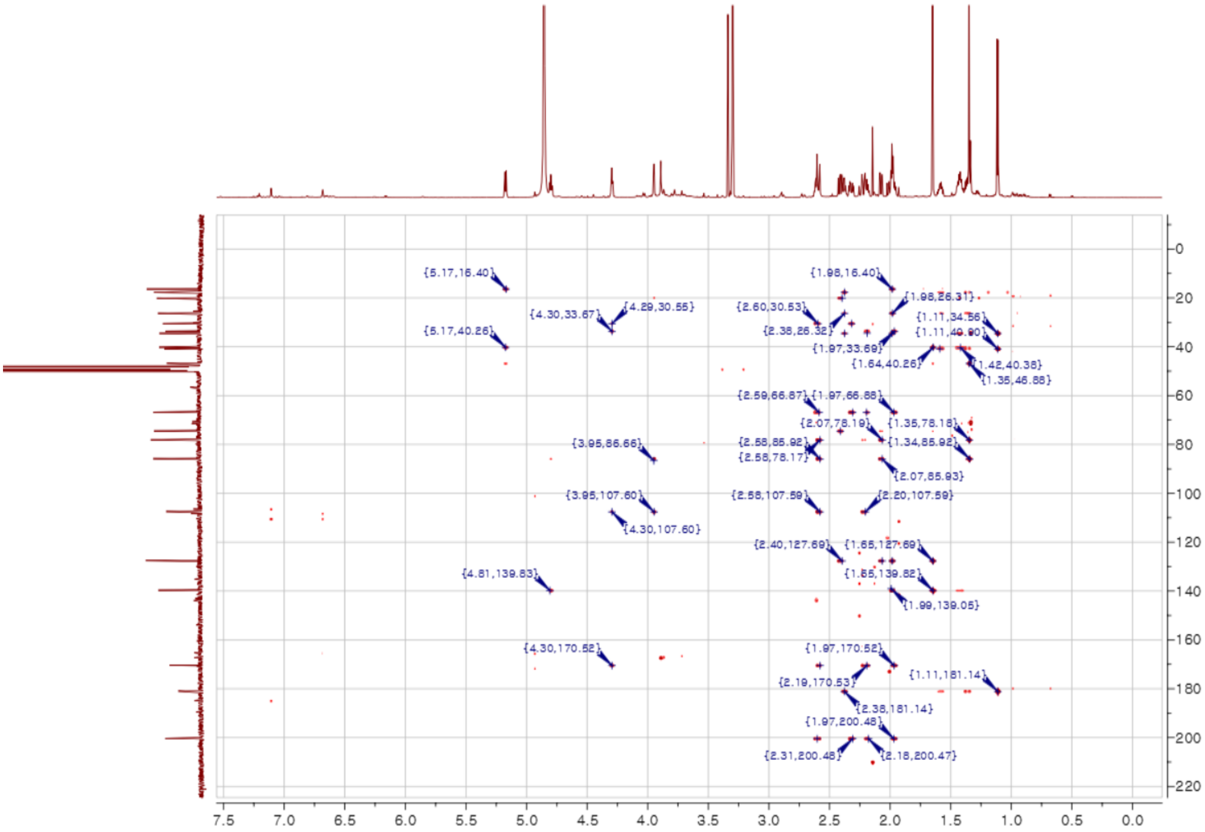

Figure S29. The HMBC spectrum of Tricycloalterfurene E (**7**) (800MHz, CD_3OD)

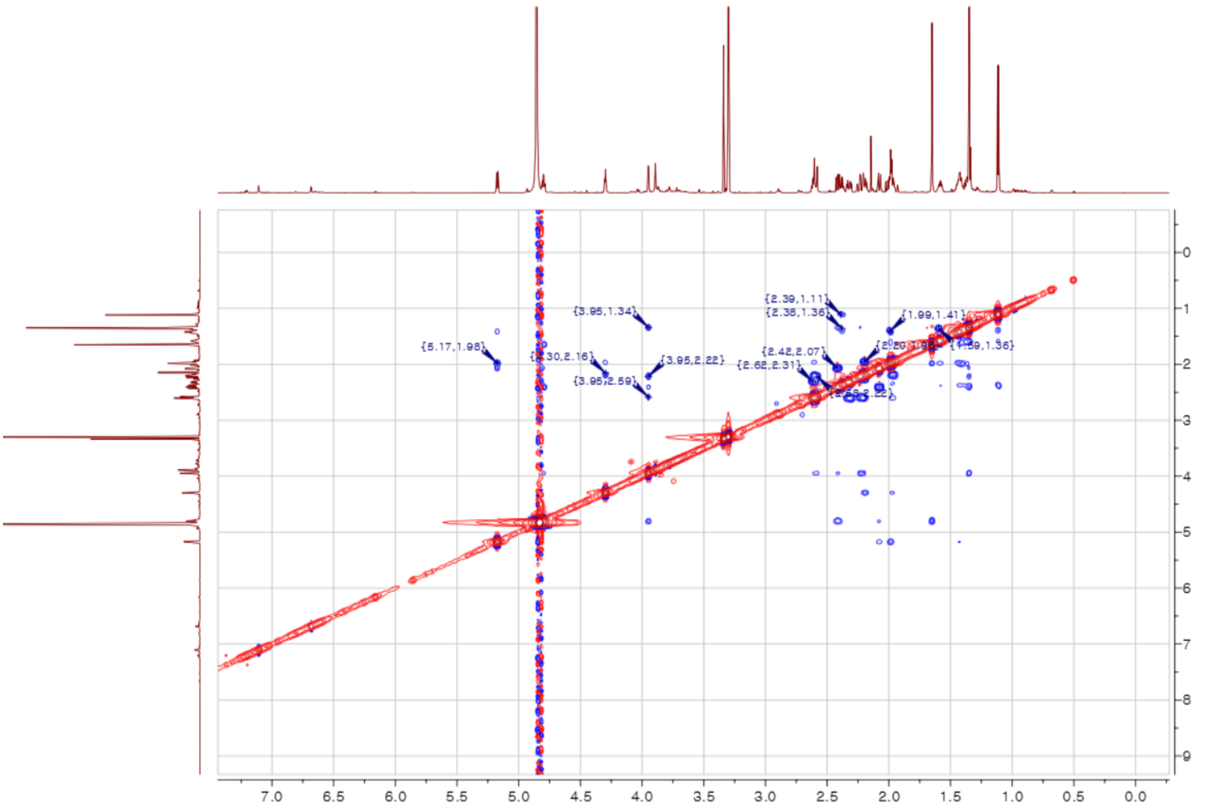

Figure S30. The NOESY spectrum of Tricycloalterfurene E (**7**) (600MHz, CD_3OD)

```
[ Elemental Composition ]                                          Page: 1
Data   : HJH-4-C21H30O6                  Date : 24-Oct-2016 14:17
Sample: -
Note : -
Inlet : Direct                           Ion Mode : FAB+
RT : 0.48 min                            Scan#: (10,11)
Elements : C 21/0, H 31/0, O 6/0
Mass Tolerance      : 1000ppm, 1mmu if m/z < 1, 3mmu if m/z > 3
Unsaturation (U.S.) : -0.5 - 100.0

Observed m/z Int%   Err[ppm / mmu]    U.S.  Composition
  378.2040    15.5     -0.6 /  -0.2    7.0  C 21 H 30 O 6

  379.2118   100.0     -0.7 /  -0.3    6.5  C 21 H 31 O 6

  380.2150    26.8

  401.1942    20.2
```

```
[ Theoretical Ion Distribution ]                                   Page: 1
Molecular Formula : C21 H31 O6
                      (m/z   379.2121, MW   379.4735, U.S.  6.5)
Base Peak :   379.2121, Averaged MW :   379.4702(a),   379.4709(w)

   m/z       INT.
  379.2121 100.0000 ***********************************************************
  380.2154  23.5855 **************
  381.2180   3.8543 **
  382.2207   0.4722
  383.2232   0.0474
  384.2258   0.0040
  385.2283   0.0003
```

Figure S31. The HRFABMS data of Tricycloalterfurene E (**7**)

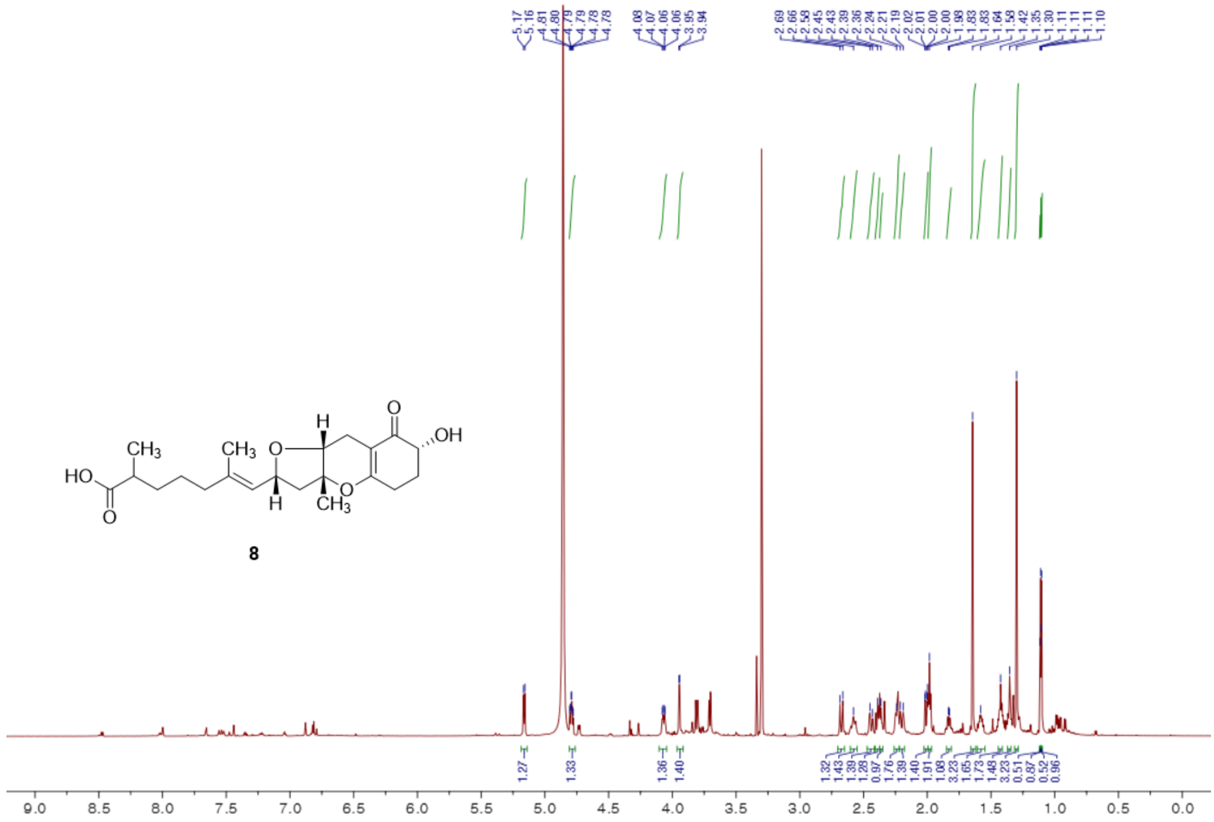

Figure S32. The ^{1}H NMR spectrum of Tricycloalterfurene F (**8**) (800MHz, CD_3OD)

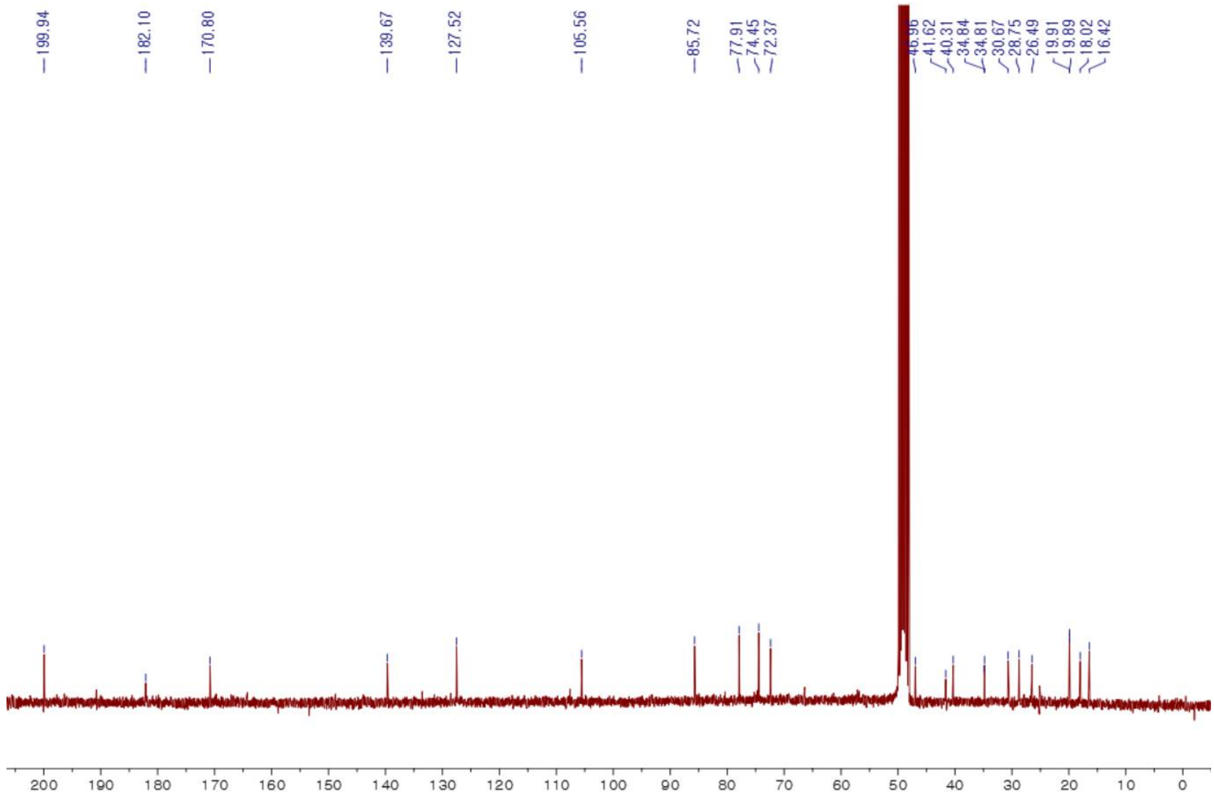

Figure S33. The ^{13}C NMR spectrum of Tricycloalterfurene F (**8**) (100MHz, CD_3OD)

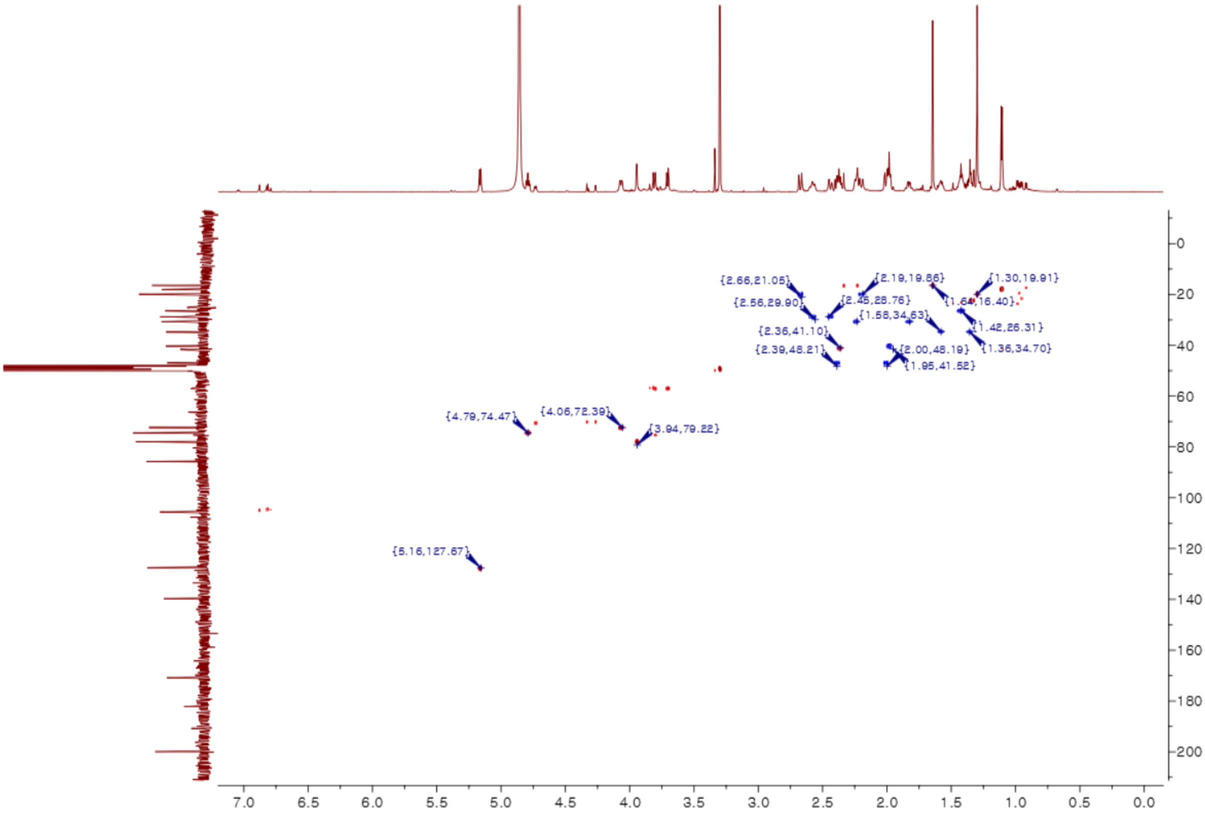

Figure S34. The HSQC spectrum of Tricycloalterfurene F (**8**) (800MHz, CD_3OD)

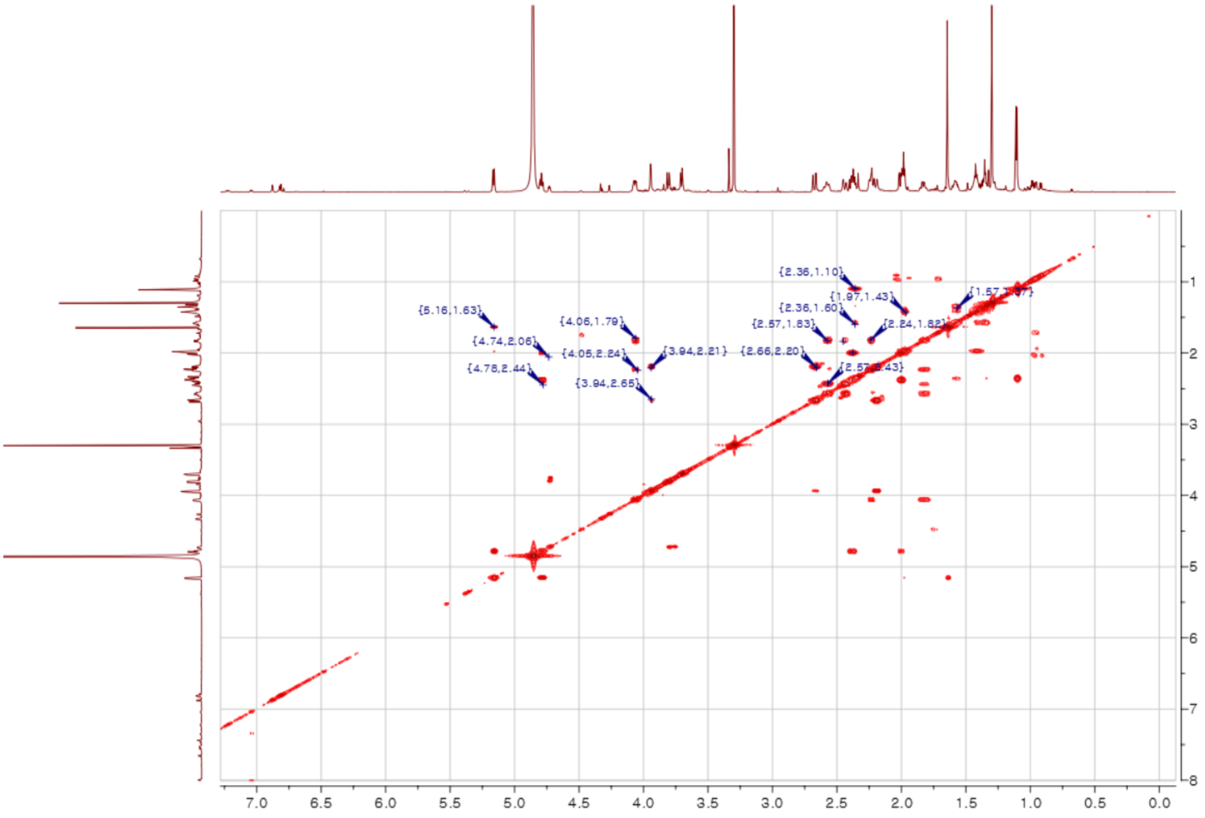

Figure S35. The COSY spectrum of Tricycloalterfurene F (**8**) (800MHz, CD_3OD)

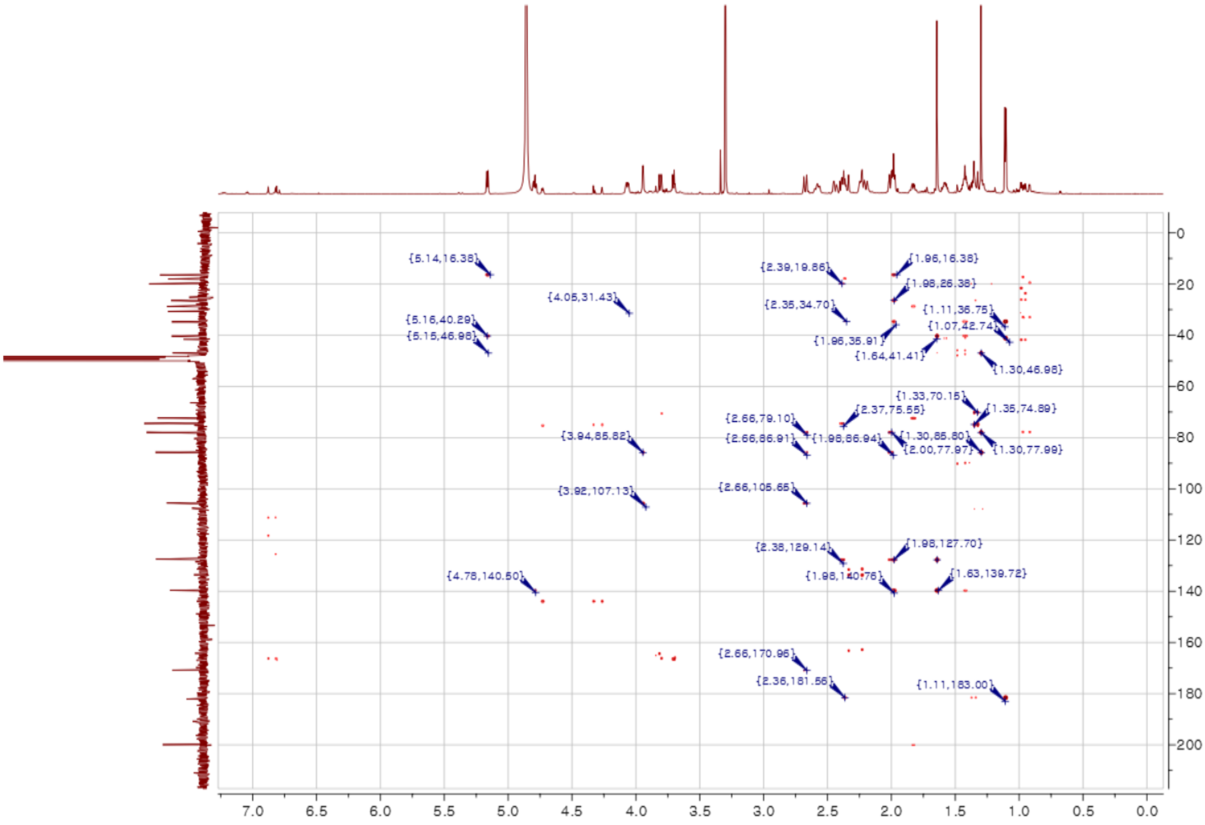

Figure S36. The HMBC spectrum of Tricycloalterfurene F (**8**) (800MHz, CD_3OD)

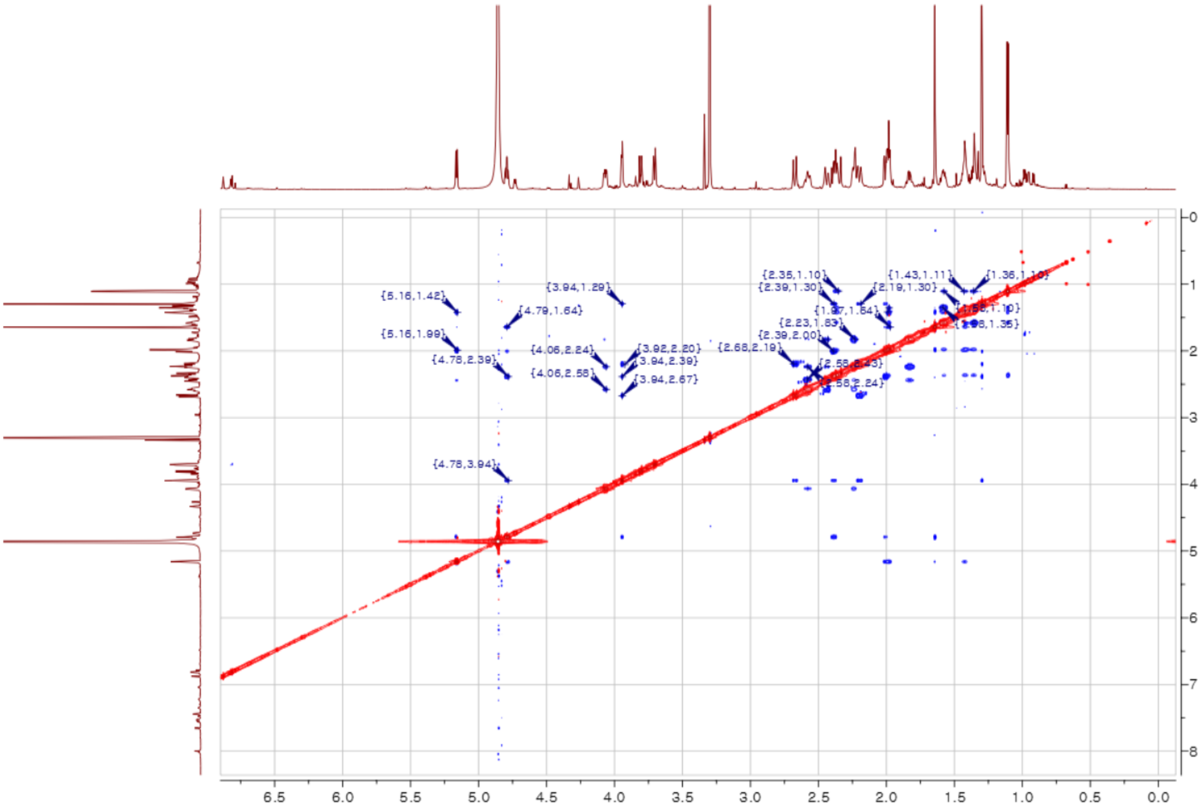

Figure S37. The NOESY spectrum of Tricycloalterfurene F (**8**) (800MHz, CD_3OD)

```
[ Elemental Composition ]                                         Page: 1
Data   : HJH-5-C21H30O6             Date : 24-Oct-2016 14:35
Sample: -
Note : -
Inlet : Direct                      Ion Mode : FAB+
RT : 0.53 min                       Scan#: (11,12)
Elements : C 21/0, H 31/0, O 6/0, Na 1/0
Mass Tolerance      : 1000ppm, 1mmu if m/z < 1, 3mmu if m/z > 3
Unsaturation (U.S.) : -0.5 - 100.0

Observed m/z Int%   Err[ppm / mmu]    U.S.  Composition
  373.1743    17.6

  379.2120   100.0     -0.1 /  +0.0    6.5  C 21 H 31 O 6

  380.2148    24.1

  401.1942    91.9     +0.4 /  +0.2    6.5  C 21 H 30 O 6 Na

  402.1957    22.1
```

```
[ Theoretical Ion Distribution ]                                  Page: 1
Molecular Formula : C21 H31 O6
                      (m/z   379.2121, MW   379.4735, U.S.  6.5)
Base Peak :   379.2121, Averaged MW :   379.4702(a),   379.4709(w)

   m/z        INT.
  379.2121 100.0000 **********************************************************
  380.2154  23.5855 **************
  381.2180   3.8543 **
  382.2207   0.4722
  383.2232   0.0474
  384.2258   0.0040
  385.2283   0.0003
```

Figure S38. The HRFABMS data of Tricycloalterfurene F (**8**)

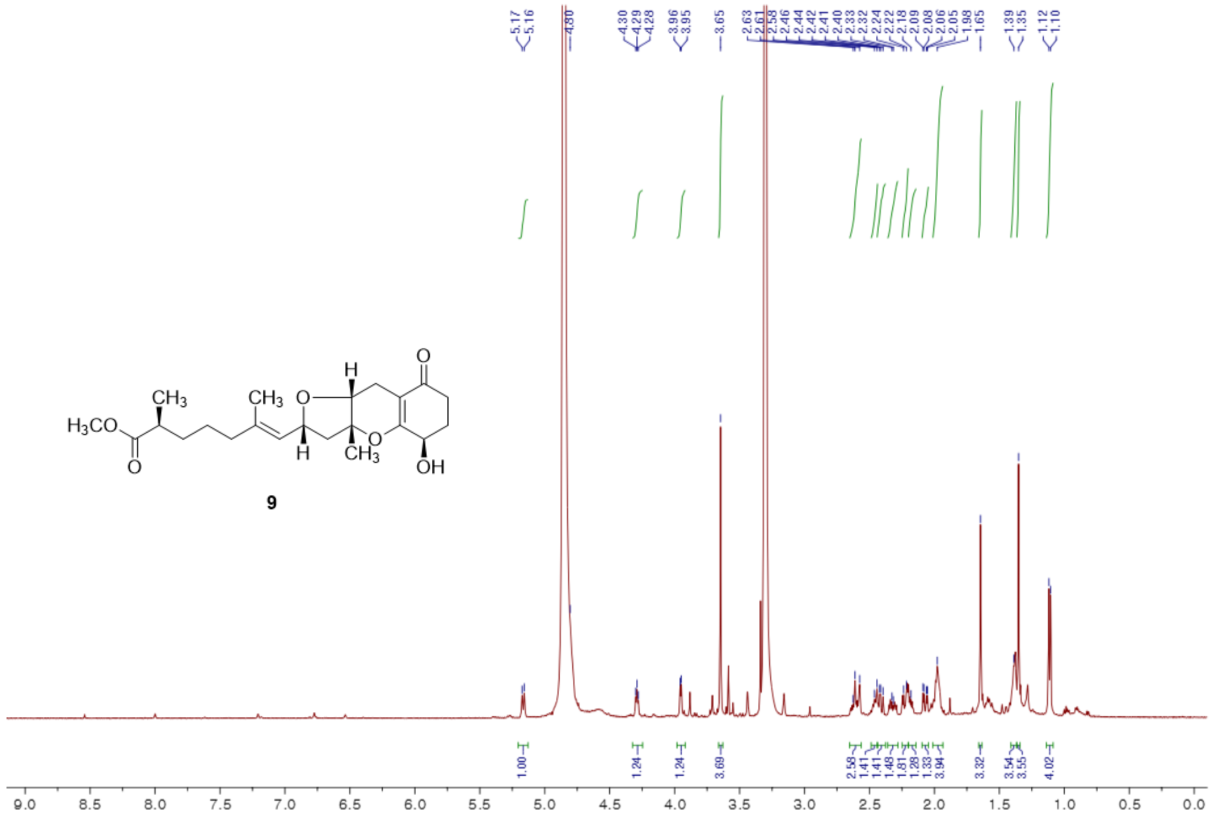

Figure S39. The ^{1}H NMR spectrum of Tricycloalterfurene G (**9**) (500MHz, CD_3OD)

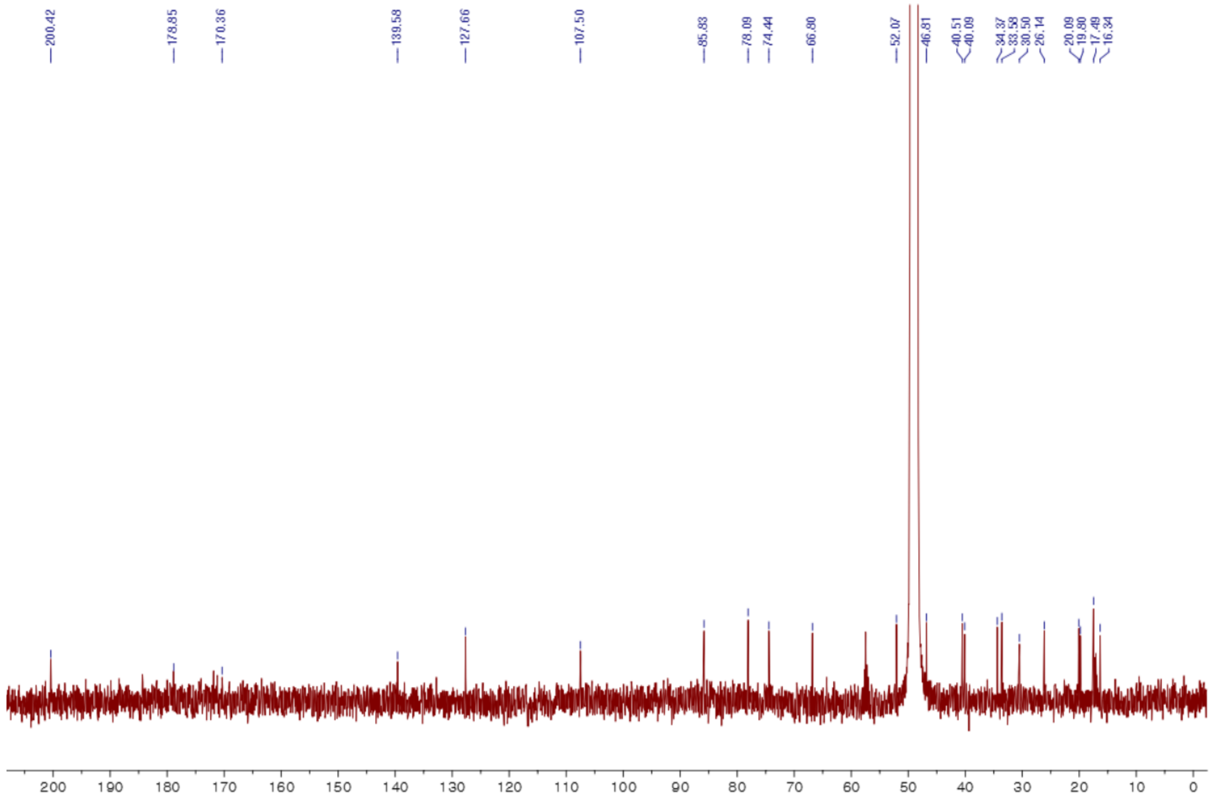

Figure S40. The ^{13}C NMR spectrum of Tricycloalterfurene G (**9**) (100MHz, CD_3OD)

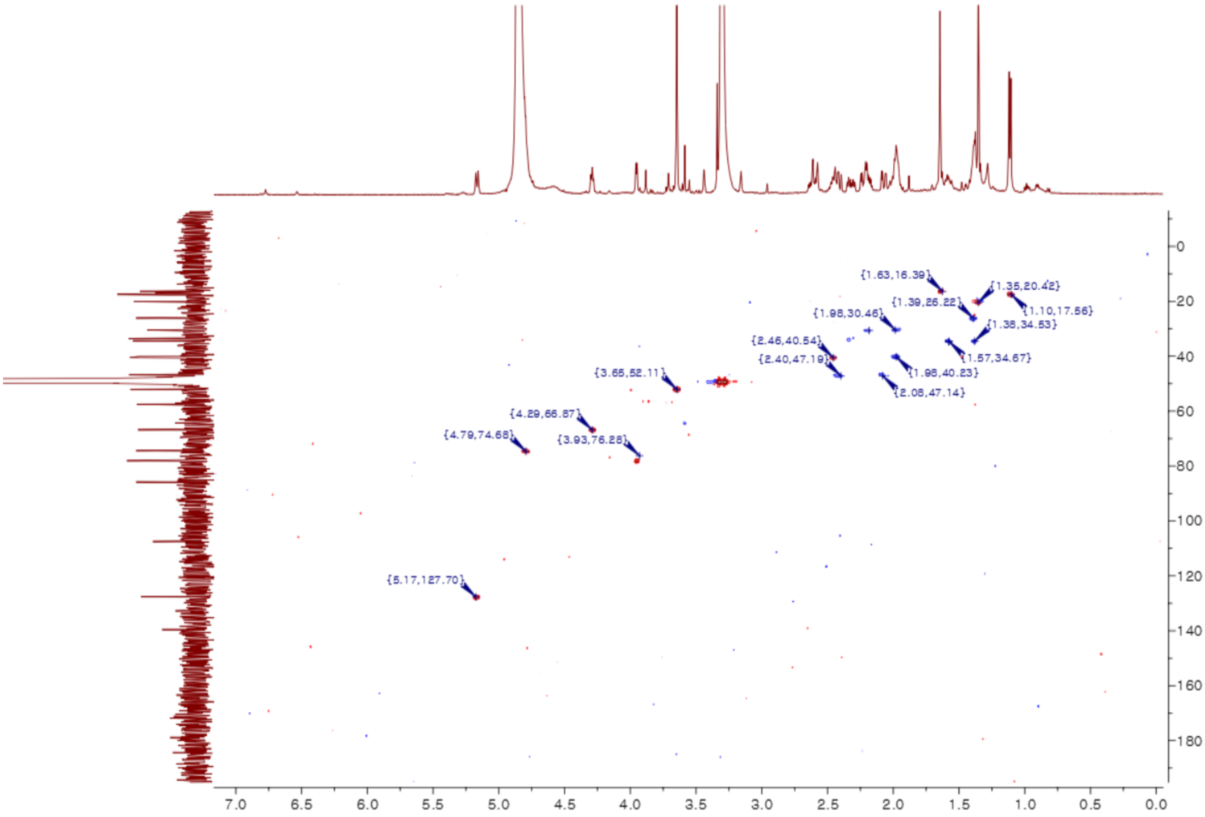

Figure S41. The HSQC spectrum of Tricycloalterfurene G (**9**) (500MHz, CD_3OD)

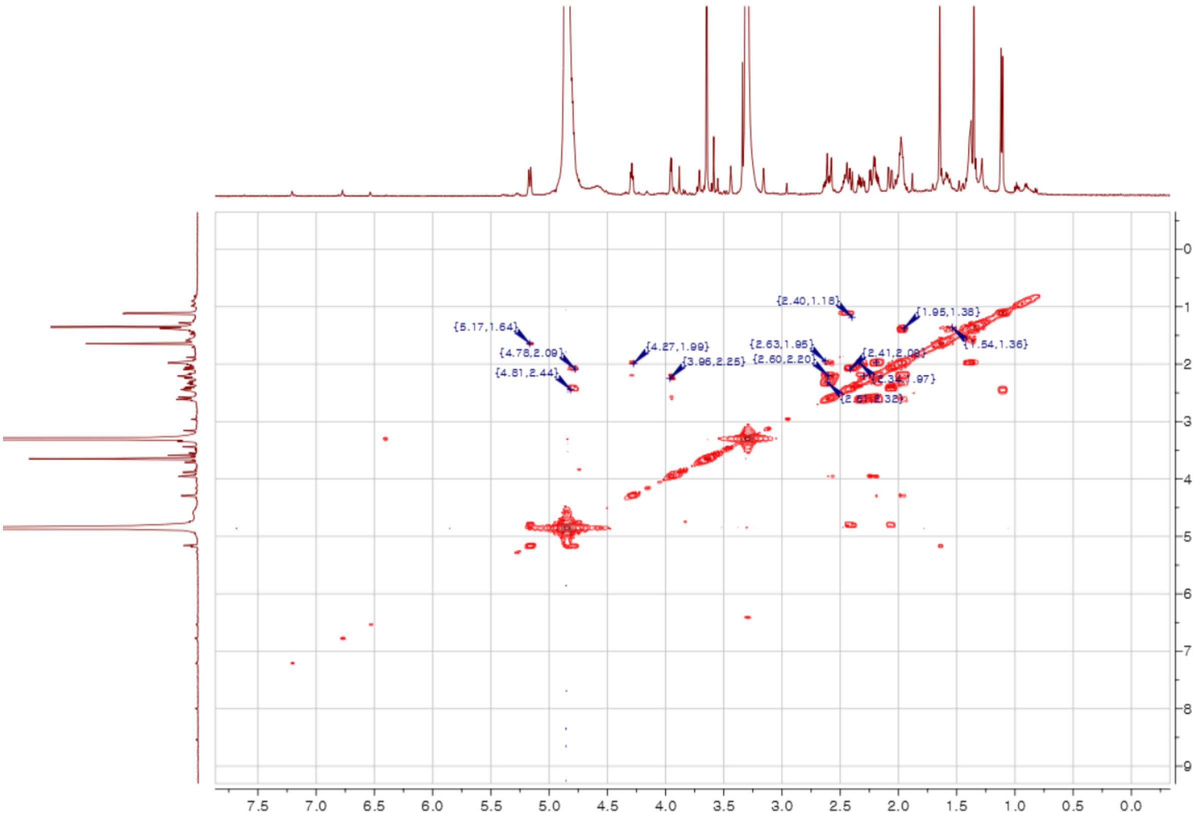

Figure S42. The COSY spectrum of Tricycloalterfurene G (**9**) (400MHz, CD_3OD)

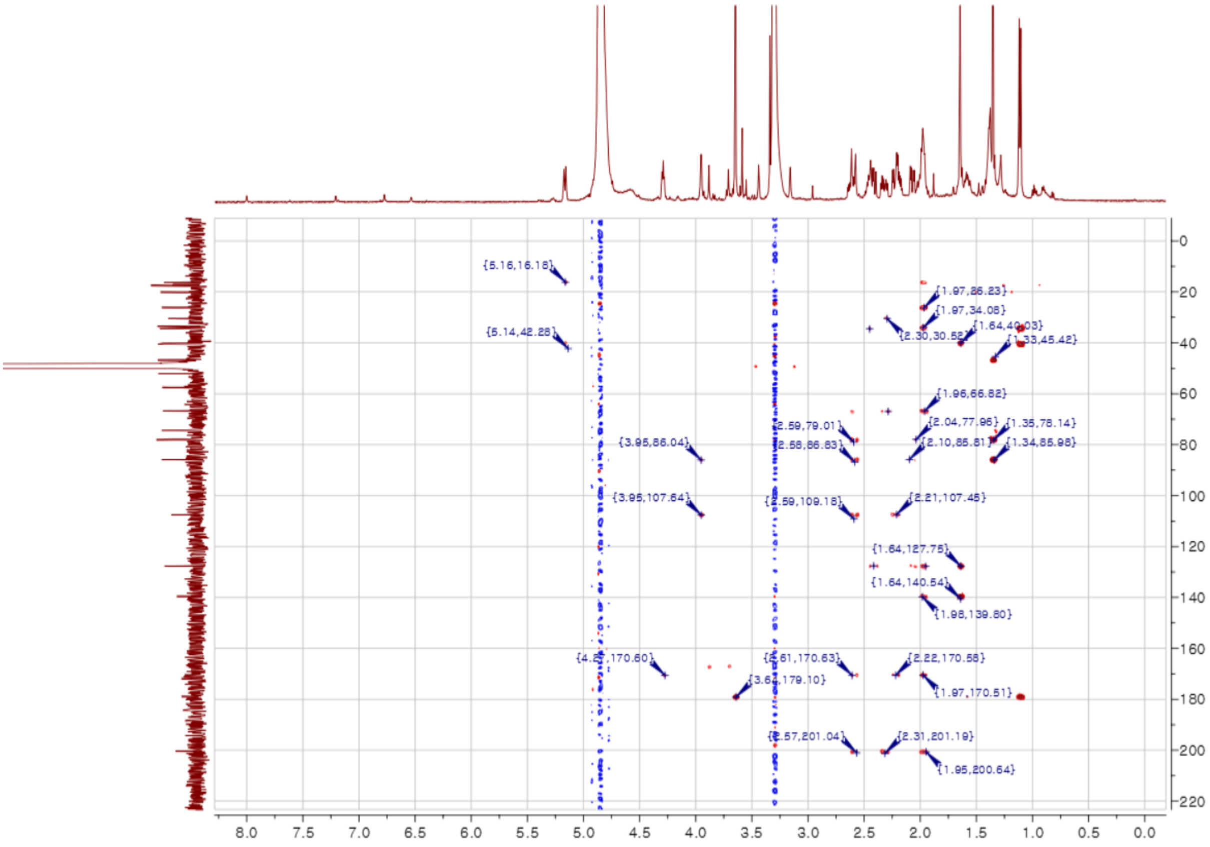

Figure S43. The HMBC spectrum of Tricycloalterfurene G (**9**) (400MHz, CD_3OD)

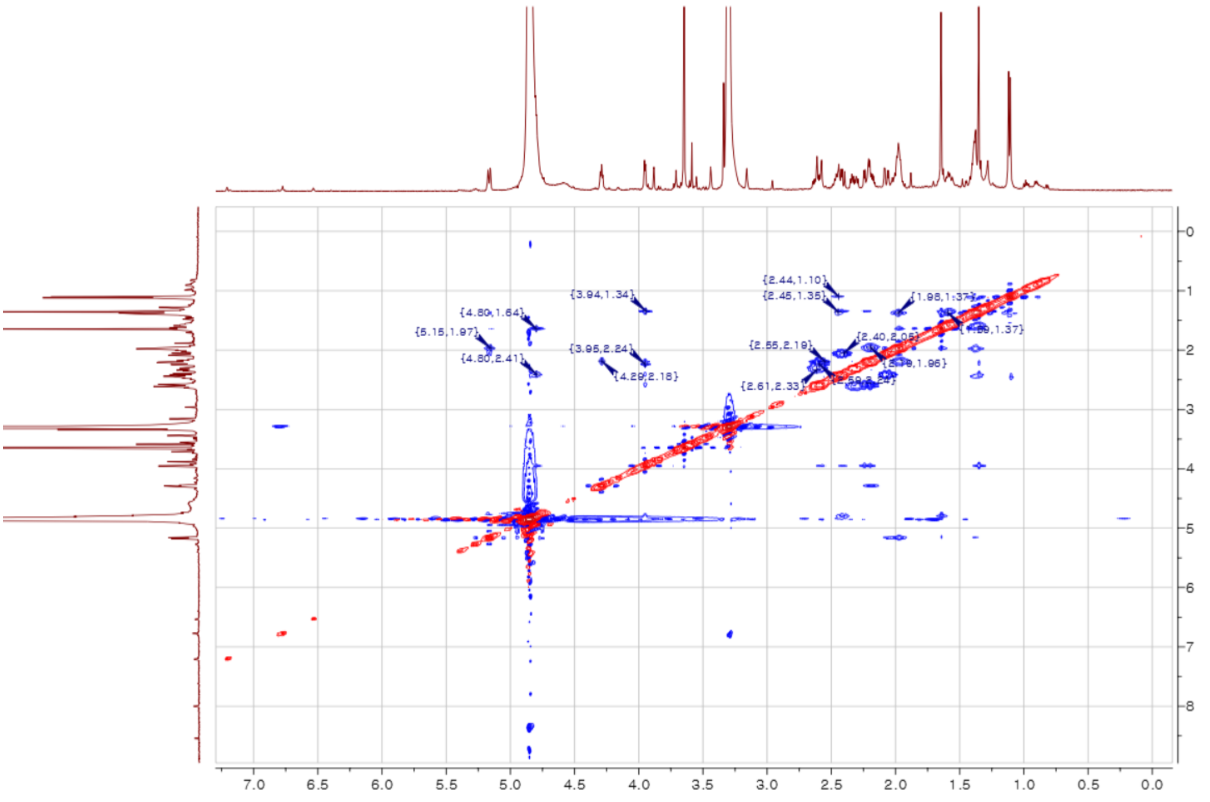

Figure S44. The NOESY spectrum of Tricycloalterfurene G (**9**) (400MHz, CD_3OD)

```
[ Elemental Composition ]                                                Page: 1
Data   : FAB-S652                    Date : 07-Jun-2019 14:21
Sample: 3[006(4)-RF3RP21]
Note : m-NBA
Inlet : Direct                       Ion Mode : FAB+
RT : 4.03 min                        Scan#: (160,164)
Elements : C 100/0, H 100/0, O 10/0, Na 1/0
Mass Tolerance      : 20ppm, 5mmu if m/z < 250, 10mmu if m/z > 500
Unsaturation (U.S.) : -0.5 - 50.0

Observed m/z  Int%    Err[ppm / mmu]    U.S.  Composition
  415.2104   100.0     +10.1 /  +4.2    18.5  C 31 H 27 O
                        -4.0 /  -1.7     9.5  C 24 H 31 O 6
                       +15.9 /  +6.6    15.5  C 29 H 28 O Na
                        +1.8 /  +0.7     6.5  C 22 H 32 O 6 Na


[ Theoretical Ion Distribution ]                                         Page: 1
Molecular Formula : C22 H32 O6 Na
                        (m/z   415.2097, MW   415.4822, U.S.  6.5)
Base Peak :    415.2097, Averaged MW :    415.4788(a),    415.4795(w)

   m/z        INT.
 415.2097 100.0000 **********************************************************
 416.2130  24.6977 **************
 417.2157   4.1166 **
 418.2183   0.5151
 419.2209   0.0526
 420.2234   0.0046
 421.2260   0.0003
```

Figure S45. The HRFABMS data of Tricycloalterfurene G (**9**)

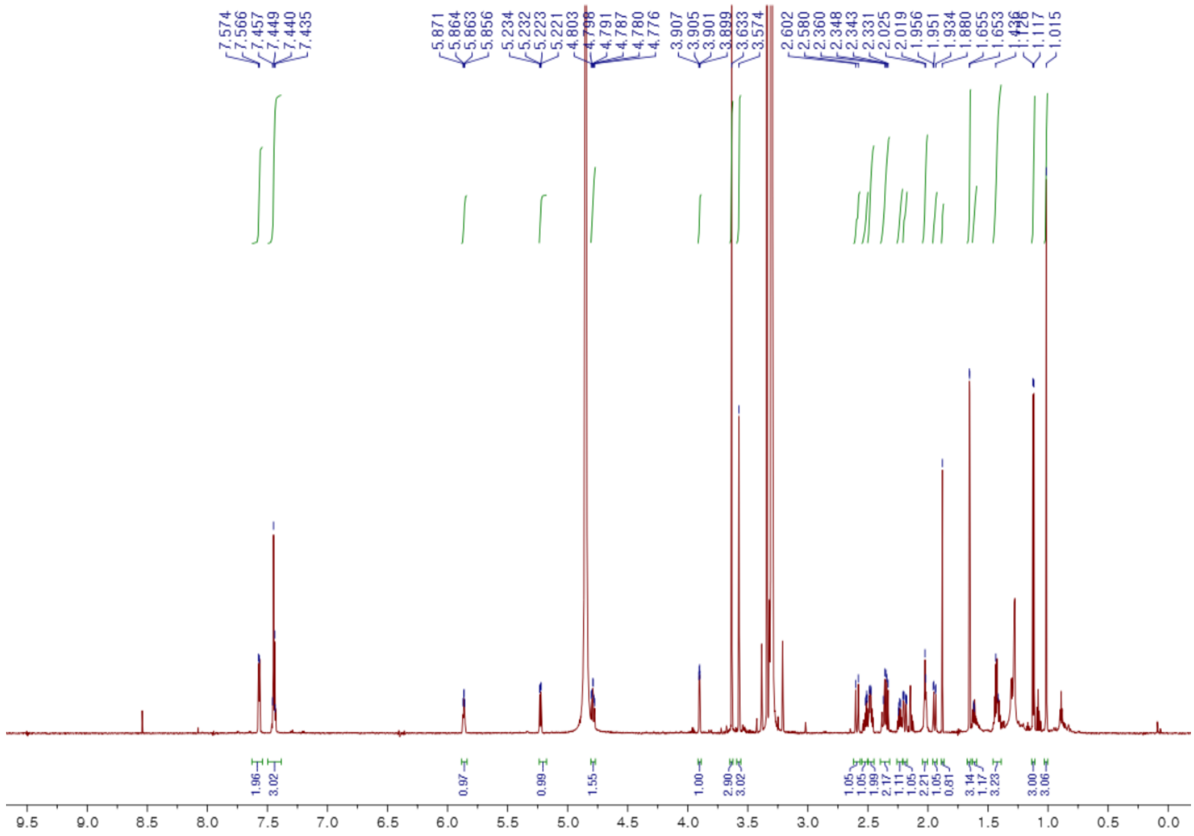

Figure S46. The ^{1}H NMR spectrum of (*S*)-MTPA Ester of **7** (800MHz, CD_3OD)

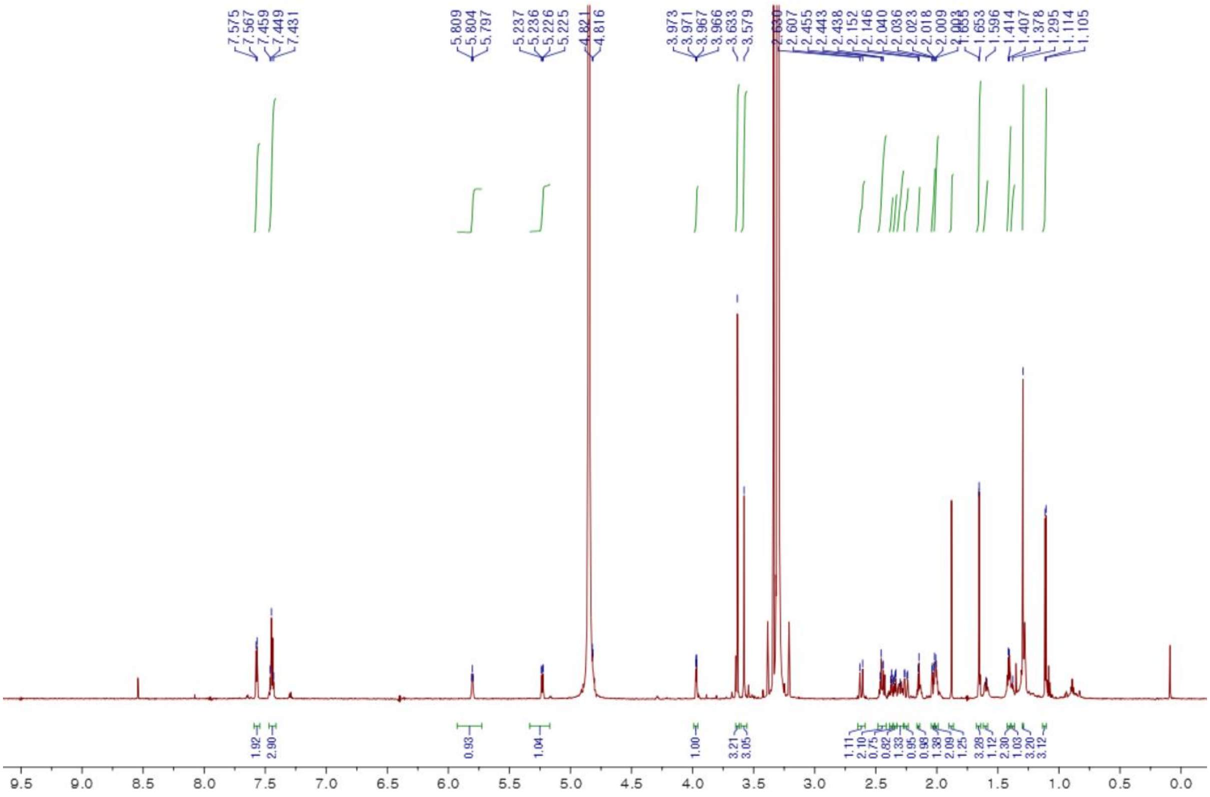

Figure S47. The ^{1}H NMR spectrum of (*R*)-MTPA Ester of **7** (800MHz, CD_3OD)

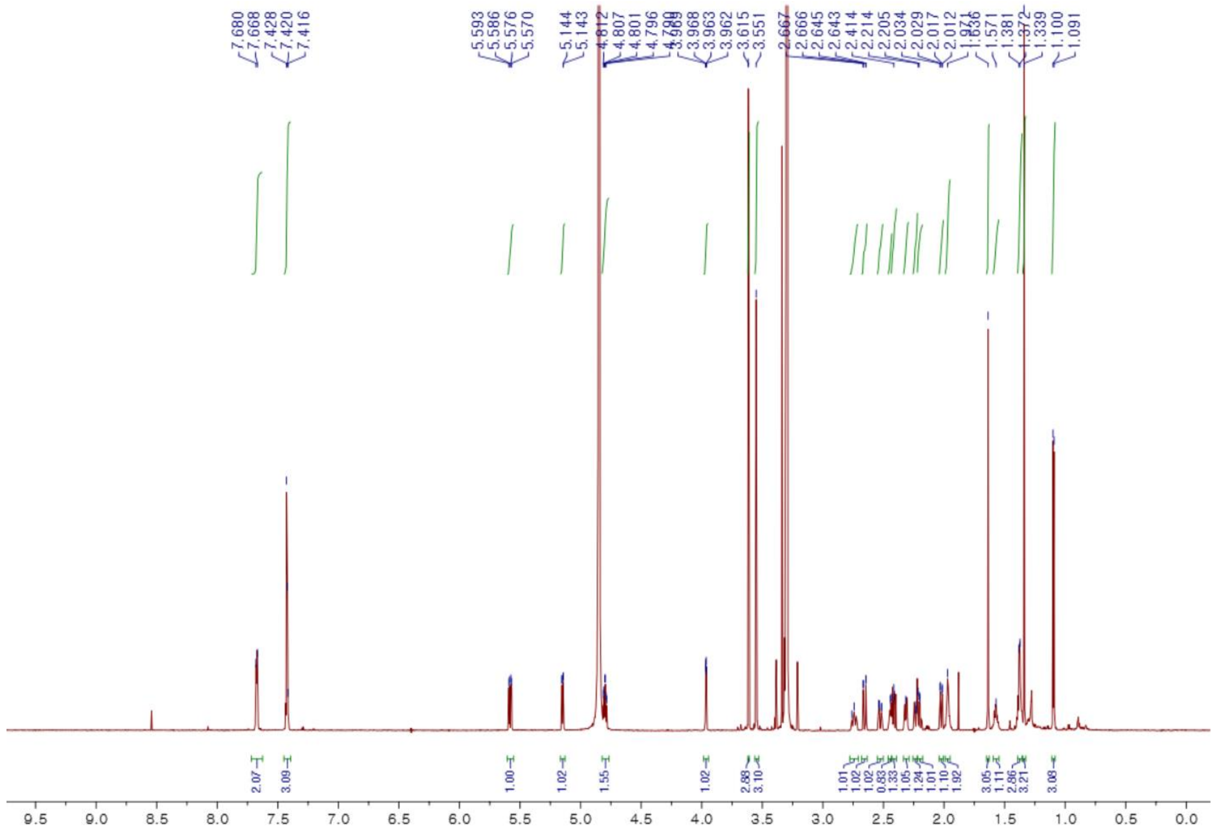

Figure S48. The ^{1}H NMR spectrum of (*S*)-MTPA Ester of **8** (800MHz, CD_3OD)

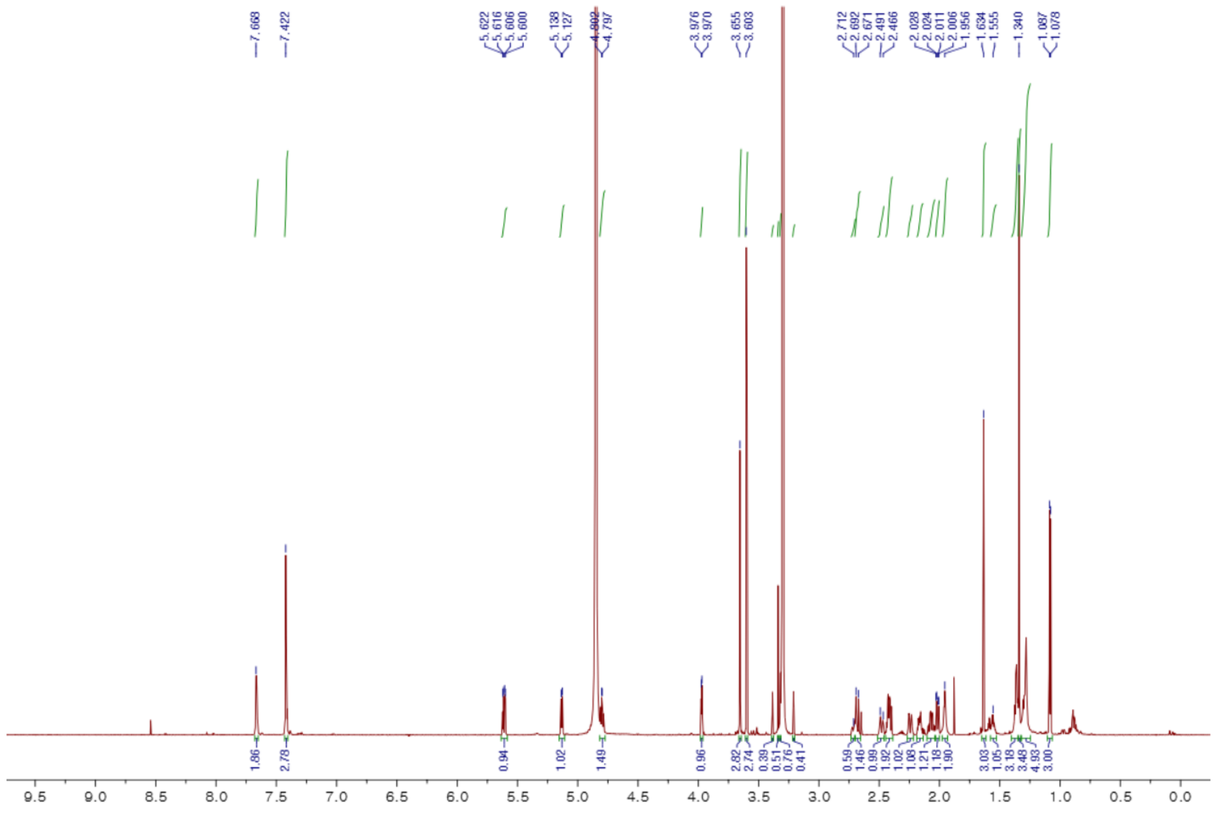

Figure S49. The ^{1}H NMR spectrum of (*R*)-MTPA Ester of **8** (800MHz, CD_3OD)

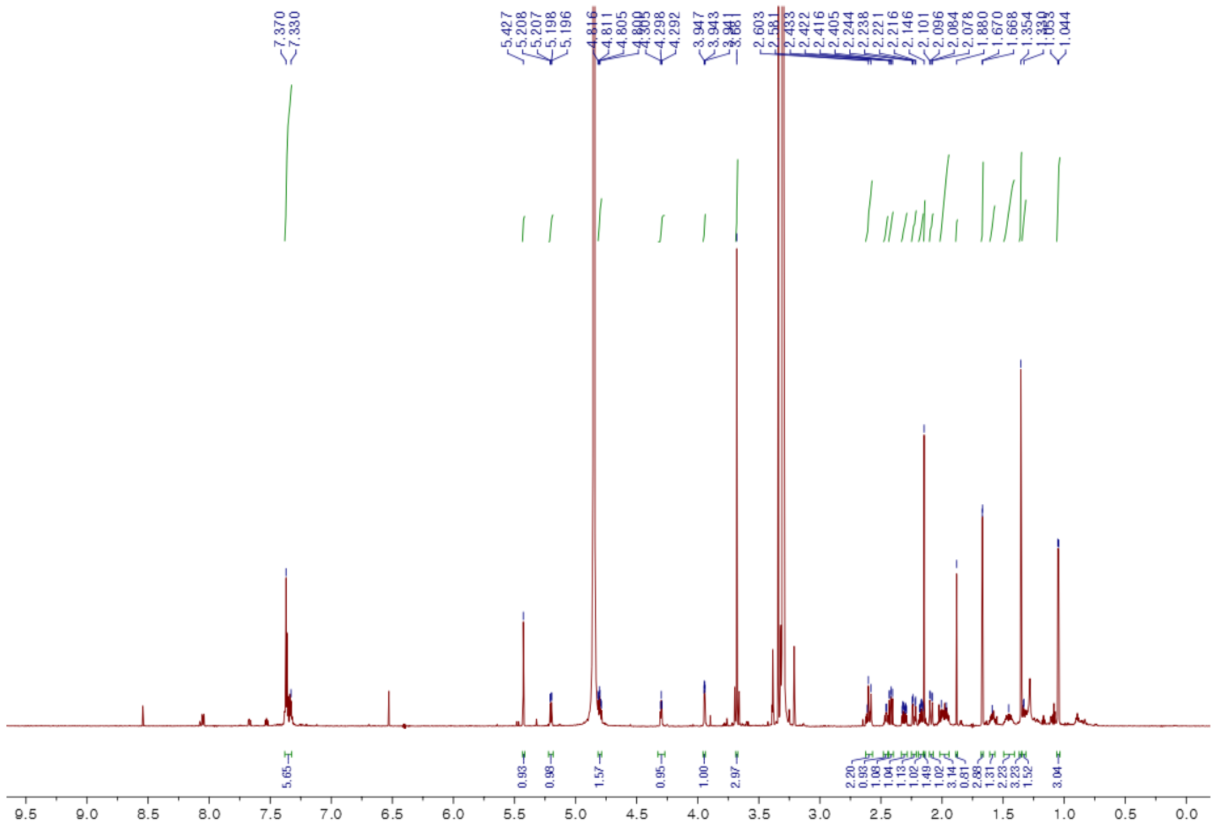

Figure S50. The ^{1}H NMR spectrum of (*S*)-PGME Amide of **7** (800MHz, CD_3OD)

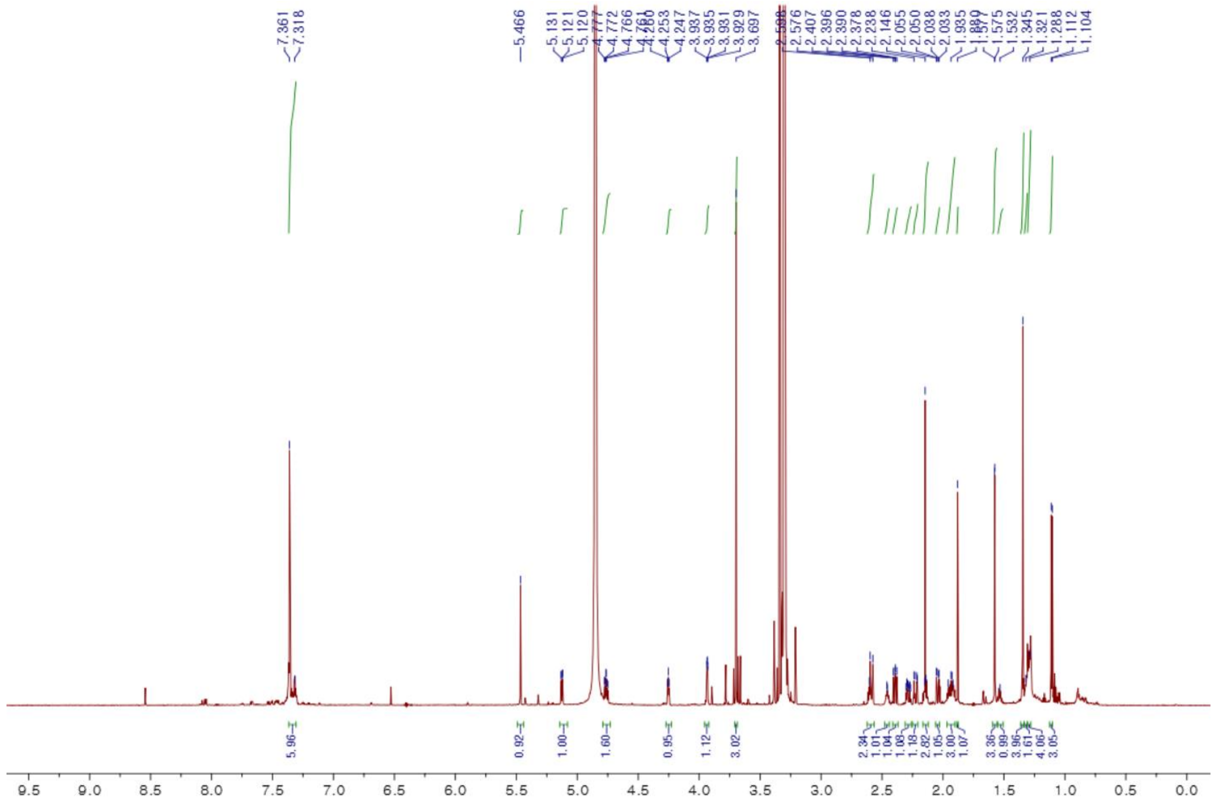

Figure S51. The ^{1}H NMR spectrum of (*R*)-PGME Amide of **7** (800MHz, CD_3OD)

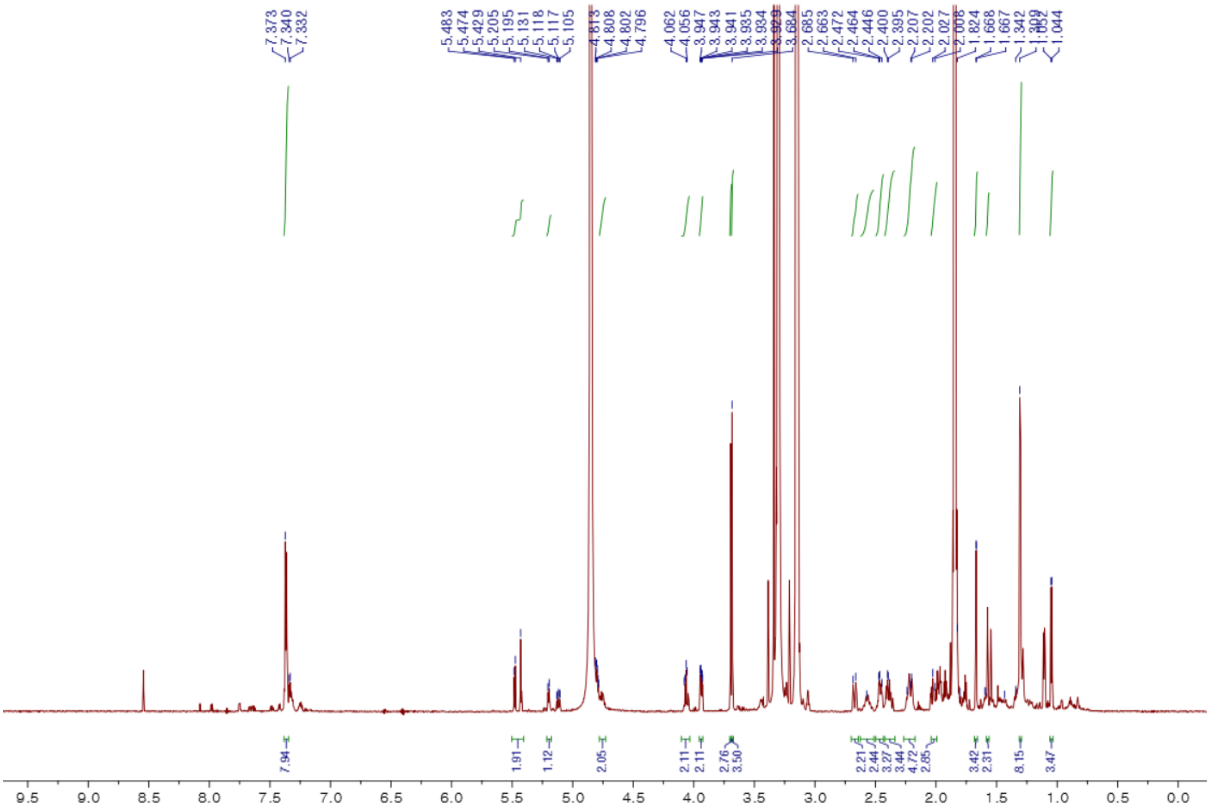

Figure S52. The ^{1}H NMR spectrum of (*S*)-PGME Amide of **8** (800MHz, CD_3OD)

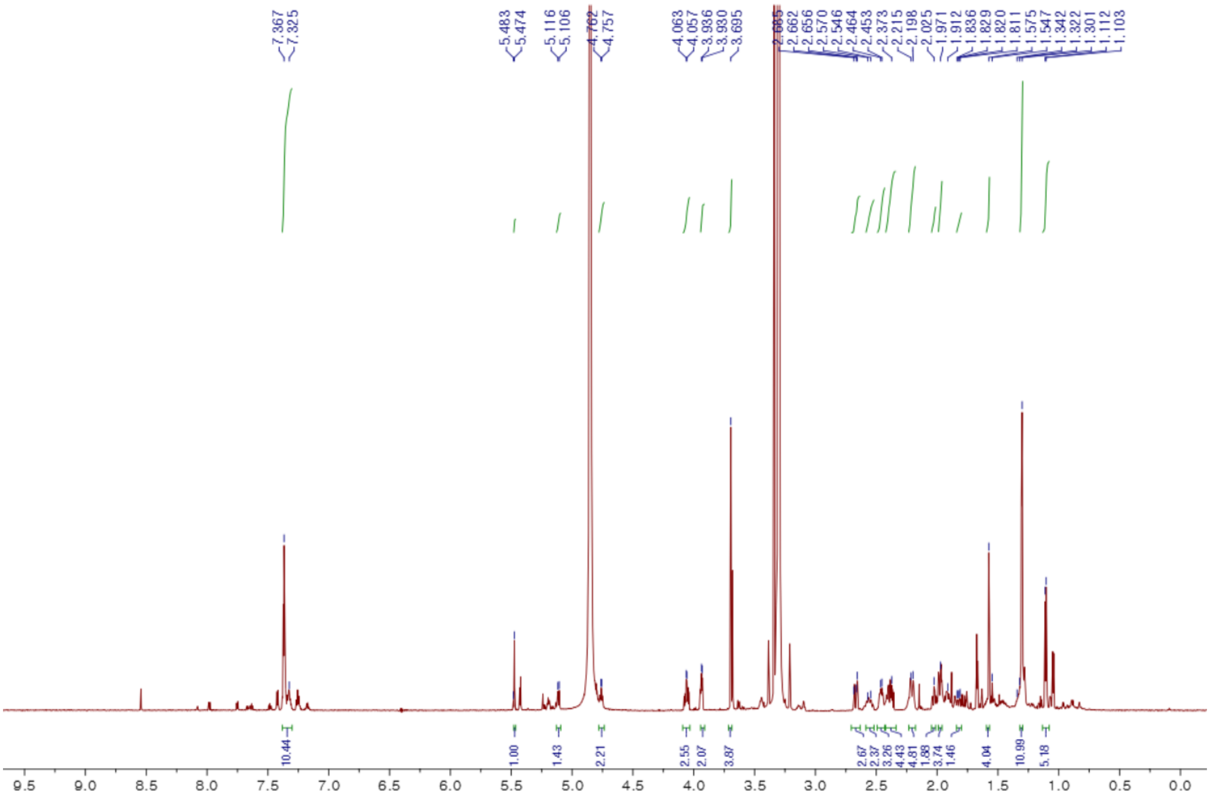

Figure S53. The ^{1}H NMR spectrum of (*R*)-PGME Amide of **8** (800MHz, CD_3OD)

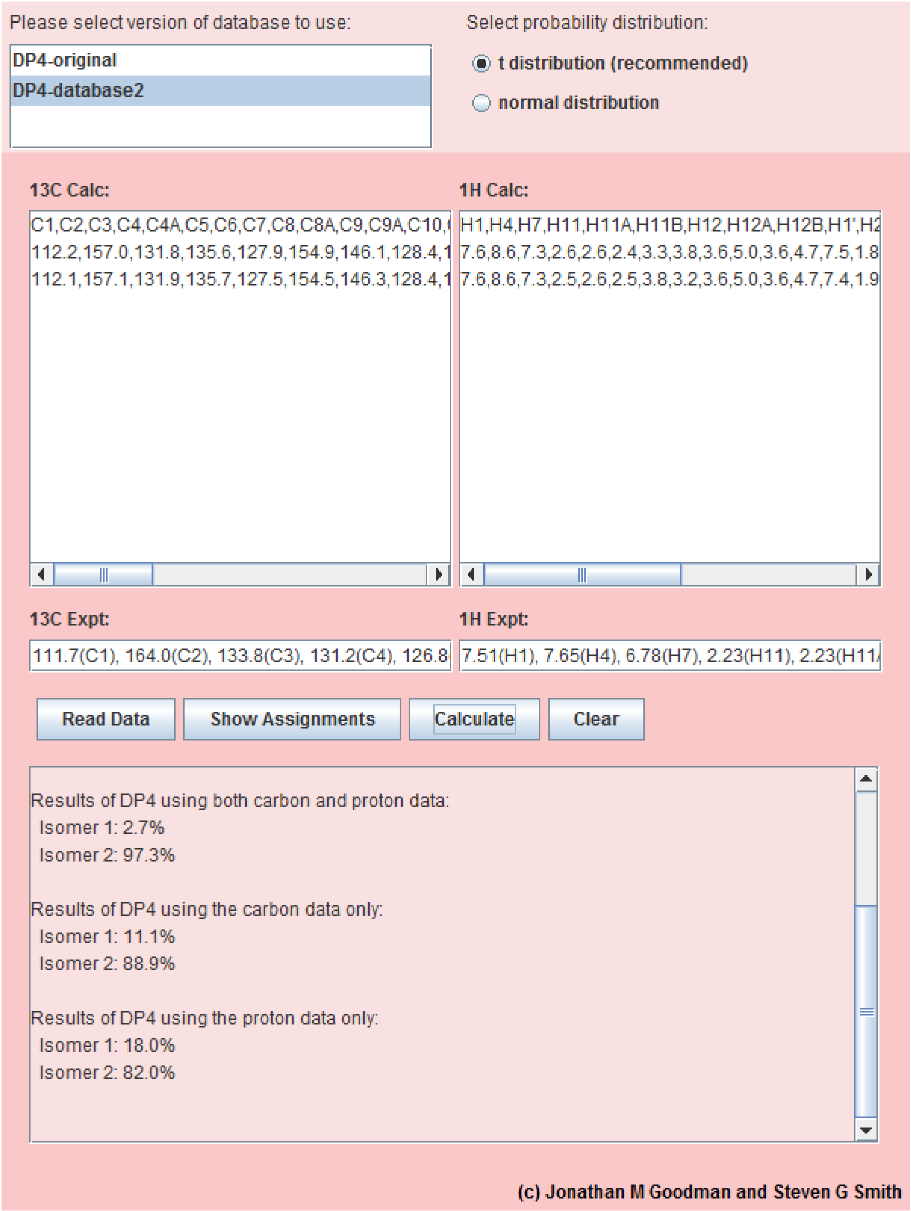

Figure S54. The results of DP4 analyses of Alterporriol Z1 (**1**)

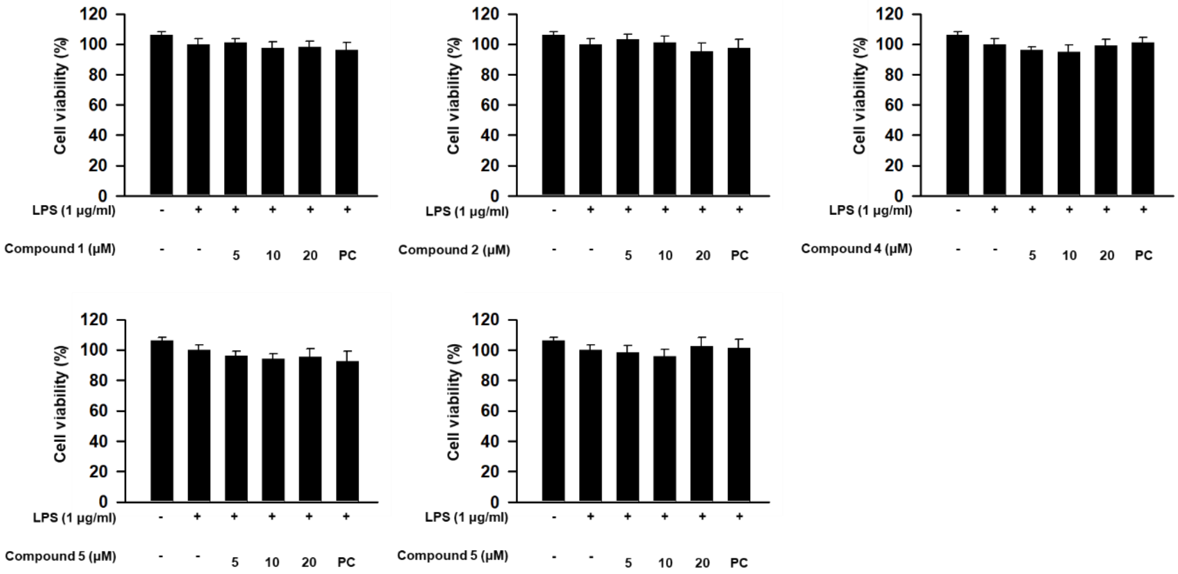

Figure S55. The viability of RAW 264.7 cells was measured using the MTT assay

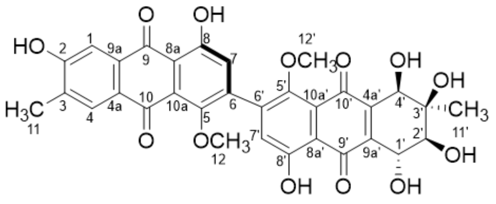

a*R*,1'*R*,2'*S*,3'*R*,4'*R*

[Isomer 1]

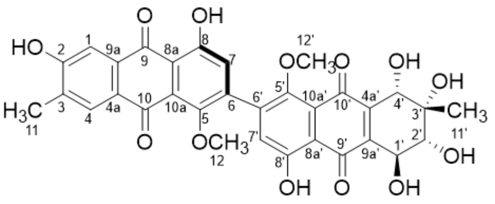

a*R*,1'*S*,2'*R*,3'*S*,4'*S*

[Isomer 2]

No.	Aterporriol Z1	
**1**	111.7, CH	7.51, d (0.5)
**2**	164.0, C	
**3**	133.8, C	
**4**	131.2, CH	7.65, d (0.5)
**4a**	126.8, C	
**5**	165.8, C	
**6**	125.3, C	
**7**	104.6, CH	6.78, s
**8**	166.9, C	
**8a**	111.9, C	
**9**	188.7, C	
**9a**	134.7, C	
**10**	183.5, C	
**10a**	133.4, C	
**11**	16.6, CH_3	2.23, s
**12**	56.9, CH_3	3.69, s
**1′**	70.6, CH	4.73, d (7.5)
**2′**	75.2, CH	3.79, d (7.5)
**3′**	74.6, C	
**4′**	70.1, CH	4.26, s
**4a′**	143.8, C	
**5′**	166.3, C	
**6′**	123.4, C	
**7′**	104.6, CH	6.81, s
**8′**	166.1, C	
**8a′**	111.0, C	
**9′**	190.5, C	
**9a′**	143.9, C	
**10′**	185.7, C	
**10a′**	130.8, C	
**11′**	22.3, CH_3	1.33, s
**12′**	57.0, CH_3	3.70, s

No.	Isomer 1	
**1**	112.2	7.6
**2**	157.0	
**3**	131.8	
**4**	135.6	8.6
**4a**	127.9	
**5**	154.9	
**6**	146.1	
**7**	128.4	7.3
**8**	157.7	
**8a**	117.4	
**9**	187.1	
**9a**	132.0	
**10**	182.7	
**10a**	124.9	
**11**	20.6	2.6,2.6,2.4
**12**	61.7	3.3,3.8,3.6
**1′**	75.0	5.0
**2′**	84.6	3.6
**3′**	77.6	
**4′**	76.0	4.7
**4a′**	141.2	
**5′**	156.4	
**6′**	146.1	
**7′**	130.0	7.5
**8′**	156.8	
**8a′**	116.3	
**9′**	188.6	
**9a′**	143.4	
**10′**	187.8	
**10a′**	124.0	
**11′**	25.9	1.8,0.9,1.9
**12′**	61.4	3.5,4.0,3.4

No.	Isomer 2	
**1**	112.1	7.6
**2**	157.1	
**3**	131.9	
**4**	135.7	8.6
**4a**	127.5	
**5**	154.5	
**6**	146.3	
**7**	128.4	7.3
**8**	158.1	
**8a**	116.9	
**9**	187.2	
**9a**	131.9	
**10**	182.7	
**10a**	124.1	
**11**	20.6	2.5,2.6,2.5
**12**	61.8	3.8,3.2,3.6
**1′**	75.1	5.0
**2′**	82.6	3.6
**3′**	77.6	
**4′**	76.1	4.7
**4a′**	141.1	
**5′**	156.9	
**6′**	146.6	
**7′**	129.5	7.4
**8′**	157.1	
**8a′**	116.2	
**9′**	189.4	
**9a′**	143.4	
**10′**	187.8	
**10a′**	123.5	
**11′**	26.7	1.9,0.9,1.7
**12′**	61.1	4.0,3.6,3.4

Table S1. Experimental (Exp.) and calculated (Cal.) chemical shift values of enantiomers A and B on aliphatic ring part of Alterporriol Z1 (**1**)

No.	**1**[a]
	δ_H (*J* in Hz)
1	7.52, s
2	
3	
4	7.70, s
4a	
5	
6	
7	6.78, s
8	
8a	
9	
9a	
10	
10a	
11	2.23, s
12	3.68, s
1′	4.64, d (7.0)
2′	3.70, d (7.0)
3′	
4′	4.12, d (6.0)
4a′	
5′	
6′	
7′	6.78, s
8′	
8a′	
9′	
9a′	
10′	
10a′	
11′	1.26, s
12′	3.69, s
13′	
1′ OH	4.29, br. s
2′ OH	4.22, br. s
3′ OH	3.78, s
4′ OH	4.70, d (6.0)

[a] Measured at 800 MHz for ^{1}H NMR

Table S2. The ^{1}H NMR Data of Alterporriol Z1 (**1**) in THF-d_8
